# Synthesis of 4-azido sialic acid for testing against Siglec-7 and in metabolic oligosaccharide engineering†

**DOI:** 10.1039/d5cb00030k

**Published:** 2025-04-17

**Authors:** Taylor E. Gray, Kristin B. Labasan, Gour C. Daskhan, Duong T. Bui, Maju Joe, Dhanraj Kumawat, Edward N. Schmidt, John S. Klassen, Matthew S. Macauley

**Affiliations:** a Department of Chemistry, University of Alberta Edmonton T6G 2G2 Canada; b Department of Medical Microbiology and Immunology, University of Alberta Edmonton T6G 2E1 Canada macauley@ualberta.ca

## Abstract

An important approach for tracking and visualizing sialic acid-containing glycans involves using sialic acid reporters functionalized with bioorthogonal handles. More specifically, metabolic oligosaccharide engineering (MOE) commonly employs monosaccharides with an alkyne or azide handle for incorporation into cellular glycans, followed by a subsequent click reaction to elaborate with a biotin or fluorophore handle. For sialic acid, this has been carried out extensively, with an azide or alkyne appended to the C5 *N*-acetamido group being the most common location for the handle. However, circumstances may require the handle to be at different positions and, to date, the C7 and C9 positions have been shown to work to varying degrees. Herein, we synthesized protected 4AzNeu5Ac that could be incorporated into cellular glycans nearly as efficiently as Neu5Az and targeted with DBCO-biotin through strain promoted azide–alkyne cycloaddition. Owing to the good incorporation of 4AzNeu5Ac into cellular glycans, we followed up this ability by first synthesizing the deprotected form of 4AzNeu5Ac, using a thioglycoside to lock the anomeric center during deprotection of the acetyl groups. Activation of 4AzNeu5Ac to CMP-4AzNeu5Ac then enabled the use of this donor by human sialyltransferase ST3GAL1 to transfer CMP-4AzNeu5Ac to β-Gal*p*-(1→3)-α-Gal*p*NAc. With purified α-4AzNeu*p*5Ac-(2→3)-β-Gal*p*-(1→3)-α-Gal*p*NAc in hand, we tested it as a ligand for Siglec-7 and found that the C4-Az modification is tolerated, opening future possibilities to exploit this position to generate high affinity and selective ligands. These findings expand the repertoire of metabolic oligosaccharide engineering agents and show that azide modifications are tolerated at the C4 position of sialic acid.

## Abstract 2

Peracetylated 4AzNeu5Ac is incorporated well into cell glycoconjugates. Deprotection *via* a thioglycoside facilitated chemoenzymatic synthesis of a C4-azido sialoside, which was studied for binding to Siglec-7 using native mass spectrometry.

## Graphical abstract

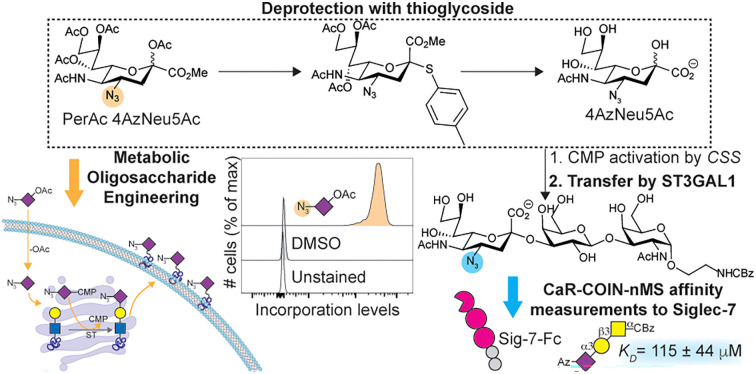

## Introduction

*N*-Acetylneuraminic acid (Neu*p*5Ac; 5-acetamido-3,5-dideoxy-d-*glycero*-d-galacto-non-2-ulosonic acid) is the most abundant of the nonulosonic sialic acids. In vertebrates, Neu5Ac terminates cell surface glycoprotein and glycolipid glycoconjugates and plays a myriad roles in both health and disease.^1^ While the diverse set of sialoglycans is a contact point for many cell–cell and cell–pathogen interactions that regulate biological roles, their involvement in many biological pathways is not fully mapped out.^2^ Elucidating the biological roles of sialic-acid containing ligands can involve genetic studies to perturb key enzymes of biosynthetic pathways,^3^ chemical inhibitors of glycosylation pathways to alter glycan processing,^4^ or *exo*-enzymatic glycan remodelling.^5,6^

Another important approach to re-engineer cellular glycans is to feed cells non-natural monosaccharides, which is referred to as metabolic oligosaccharide engineering (MOE).^7–9^ Incorporating azide-tagged sialic acids into cell surface glycans was one of the earliest forms of MOE.^9–11^ This can be achieved by treating cells with *N*-azidoacetylneuraminic acid (Neu*p*5Az, methyl-5-azidoacetamido-2,4,7,8,9-penta-*O*-acetyl-3,5-trideoxy-β-d-*erythro*-l-manno-2-nonulo-pyranosonate), or its precursor *N*-azidoacetylmannosamine (Man*p*NAz, *N*-azidoacetyl-3,5-dideoxy-d-*erythro*-α-l-manno-2-nonulopyranosonate) that enters the sialic acid biosynthetic pathway and is converted to Neu5Az through a series of three enzymatic steps. Cytidine monophosphate *N*-acetylneuraminic acid synthetase activates Neu5Az to cytidine monophosphate (CMP)-Neu5Az, which is then transported into the Golgi apparatus by a solute carrier transporter, transferred onto acceptor glycans by sialyltransferases (STs), and delivered to the cell surface. Once displayed on the cell surface, the azide tag can be leveraged in a bioorthogonal reaction to elaborate the azide with a reporter (*e.g.* biotin or fluorophore) *via* a copper-catalysed azide–alkyne cycloaddition (CuAAC) or a strain promoted azide–alkyne click chemistry (SPAAC) reaction using dibenzylcyclooctyne (DBCO), for visualization and quantification by microscopy or flow cytometry. An alternative application is to elaborate the azide with diverse functional groups appended to the triazole linker to engage glycan-binding proteins.^12–17^

While placing the azide group off the 5-acetamido group is logical given the compatibility of this location with the metabolic machinery, circumstances may arise where it is preferable to have the azide group at another position. In this respect, the use of C7-(7AzNeu*p*5Ac, 5-acetamido-7-azido-3,5,7-trideoxy-d-*glycero*-α-d-galacto-nonulopyranosic acid) and C9-(9AzNeu*p*5Ac, 5-acetamido-9-azido-3,5,9-trideoxy-d-*glycero*-α-d-galacto-nonulopyranosic acid) azido sialic acids in MOE have been described.^18,19^ Modifications at the C4 position of sialic acid have shown promise in the development of ligands for glycan binding proteins. Aromatic moieties at C4 (piperidine, piperazine) have previously been shown to bind and inhibit bacterial sialic acid uptake transporters, *Proteus mirabilis* SiaT, which provides bacteria uncapable of biosynthesizing sialic acid a mechanism to evade recognition by immune systems through molecular mimicry.^20^ It has also been shown that the affinity of sialosides for CD22 can be enhanced by C4 modifications.^21,22^ More recently, Siglec-7 was shown to interact with the Lipopolysaccharide (LPS) *O*-antigen from *F. nucleatum*,^23^ a bacteria associated with dental disease and is increasingly recognized to be associated with cancer.^24–26^ Notably, this *O*-antigen contains a repeating trisaccharide unit –[-4-β-Gal*p*-3-α-Fuc*p*NAc4N-4-α-Neu*p*NAc-]– that contains an α1→4 linked sialic acid.^27,28^ Therefore, the implication is that Siglec-7 may be able to tolerate C4 modifications, although this has never been formally tested. Given the potential applications of C4 modifications on sialic acid, we were motivated to test if a C4-azide on sialic acid could be used in MOE.

Preparation of Neu5Az,^12,29^ 7AzNeu5Ac,^19,30,31^ and 9AzNeu5Ac^17,32,33^ are well described and relatively facile given that the azide group can be installed on ManNAc, followed by enzymatic conversion to its corresponding Neu5Ac by a bacterial sialic acid aldolase. In many cases, the desired compound to be used in MOE is a fully peracetylated and methyl ester version of the compound, which greatly helps improve cellular uptake.^34,35^ Unlike Neu5Az, 7AzNeu5Ac, and 9AzNeu5Ac, where the azide group is installed on ManNAc and enzymatically converted to sialic acid, 4-azido-*N*-acetylneuraminic acid (4AzNeu*p*5Ac, 5-acetamido-4-azido-3,4,5-trideoxy-d-*glycero*-α-d-galacto-nonulopyranosic acid) cannot be prepared in this way. Instead, the azide group must be installed on the sialic acid. Indeed, an azide can be installed stereoselectively at C4 starting from an oxazoline derivative of *N*-acetyl-2,3-dehydro-2-deoxyneuraminic acid (Neu*p*5Ac2en; DANA) through a nucleophilic ring opening of the oxazoline between C4 and C5.^36^ With the 4-azido Neu5Ac2en on hand, selective debromination can be performed followed by acetylation of C2–OH to give fully protected 4AzNeu5Ac. However, deprotection of peracetylated 4AzNeu5Ac under basic conditions is a particular synthetic challenge, as sugars are prone to alkaline degradation in aqueous solutions.^37–41^ Alkaline degradation at the reducing end of carbohydrates results in the cleaving of the glycosidic linkage and conversion of the residue into hydroxy acid, hence described as a ‘peeling’ reaction.^37^ In the case of sialic acid in aqueous solutions, the free C2-hydroxyl group allows it to be in equilibrium with its open-chain form generating the alpha-keto acid. We hypothesize that when Neu5Ac, in its alpha-keto form, the C3 hydrogens become highly acidic (p*K*_a_ ∼ 16–20 for the Hα next to the ketone) promoting base-catalyzed elimination reactions to occur. Moreover, no detailed explanation is yet available for the alkali stability of C4-modified Neu5Ac, which may be why fully deprotected 4AzNeu5Ac has not been reported yet. We also hypothesize that the presence of an electron-withdrawing azide group at the C4 position further promotes the unwanted base-catalyzed elimination reactions. These peeling reactions generate decomposition by-products alongside the free sugar during the deprotection step resulting in extremely low yields.

Here, we present an efficient synthetic route to access 4AzNeu5Ac by installing a thioglycoside to protect the anomeric center, enabling deprotection of the acetyl protection groups without a destructive ‘peeling’ reaction. Solving this synthetic challenge enabled access to 4AzNeu5Ac, and conversion of 4AzNeu5Ac into CMP-4AzNeu5Ac facilitated its enzymatic transfer by human ST3GAL1 to make a C4Az-modified sialoside that we demonstrate binds Siglec-7 with similar affinity compared to the unmodified sialoside. We also show synthesis of protected 4AzNeu5Ac and demonstrate its use in MOE. These findings open the possibility of leveraging 4AzNeu5Ac more in chemical biology applications exploring the roles of sialic acid and sialic acid binding proteins.

## Experimental

### MOE experiments

U937 cells (20 000 cells per well) were seeded in a 96-well plate with azide-modified sugars or dimethyl sulfoxide (DMSO) vehicle control for 3 days. Overall DMSO percentage incubated with cells was 0.25%. Cells were washed with phosphate buffered saline (PBS) and resuspended in 100 μL 10 μM biotin-PEG4-DBCO from vector laboratories. Cells were left to react at room temperature for 1 hour. Cells were then washed twice with PBS and incubated on ice in 50 μL Streptavidin-PE (Biolegend; 0.2 μg mL^−1^) for 45 min. Cells were washed with PBS and resuspended in 200 μL flow buffer (Hanks’ balanced salt solution containing 1 mg mL^−1^ bovine serum albumin) for flow cytometry performed on a 5-laser Fortessa X-20 (BD Bioscience). All the resulting data were analyzed using FlowJo (10.5.3) software (BD Biosciences) and processed using GraphPad Prism version 10.2.3.

### Microscopy

U937 cells (60 000 cells per well) were seeded in a 12 well plate with 300 μM peracetylated azide sugars 6, 7, or DMSO control. After three days, cells were harvested and pelleted in a U-bottom plate at 330 rpm for 5 min. Cells were washed with PBS and resuspended in 100 μL 10 μM biotin-PEG4-DBCO from vector laboratories. Cells were left to react at room temperature for 1 hour. Cells were then washed twice with PBS and incubated on ice in 100 μL streptavidin-APC (Biolegend; 2.5 μg mL^−1^) for 45 min. Cells were again washed and fixed with 80 μL 2% paraformaldehyde for 10 min on ice. Cells were washed twice with PBS and resuspended in Hoescht stain 2 μg mL^−1^ for 15 min on ice. Cells were washed with PBS, resuspended in 80 μL PBS. 10 μL cells were dropped onto a glass slide and dried. Once dried on the slide, 10 μL mounting media (Fluoro-gel, Fisher Scientific) was added to each spot and a glass coverslip was placed overtop and secured. Slides were imaged on a ImageXpress Pico Cell imaging system (Molecular Devices).

### General chemicals

All reagents were purchased from Sigma-Aldrich, Canada. CDCl_3_ (chloroform D) CD_3_OD (methanol-D4), and D_2_O (deuterium oxide) were purchased from Deutero GmbH. Other solvents (analytical and HPLC grade) and reagents were purchased from Aldrich and were used as received. Reactions were monitored by analytical TLC on Silica Gel 60 F_254_ (0.25 mm, E. Merck). Reactions were carried out in oven-dried glassware. All reagents were purchased from commercial sources and were used without further purification unless noted. Reaction solvents were purified by successive passage through columns of alumina and copper under nitrogen. Unless stated otherwise, all reactions were carried out at room temperature under a positive pressure of argon and were monitored by TLC on Silica Gel 60 F_254_. Spots were detected under UV light (*λ*_max_ = 254 nm) or by charring with acidified *p*-anisaldehyde solution in EtOH. Unless otherwise indicated, all column chromatography was performed on Silica Gel (40–60 μM). NMR experiments were conducted on a Varian 500, 600 and 700 MHz instrument in the Chemistry NMR Facility, University of Alberta. For ^1^H and ^13^C spectra, chemical shifts are expressed as parts per million (ppm, *δ*) and are relative to the solvent used. ^1^H chemical shifts are reported relative to the deuterated solvent peak to either TMS (0.0, CDCl_3_) or CD_3_OD (3.30, CD_3_OD) or HOD (4.78, D_2_O). ^1^H data were reported as though they were first order. ^13^C NMR (APT) spectra were recorded at 176 MHz or 125 MHz, and ^13^C chemical shifts were referenced to internal CDCl_3_ (77.23, CDCl_3_), or CD_3_OD (48.90, CD_3_OD) or external acetone (31.07, D_2_O). Coupling constants (*J*) are reported in Hz and apparent multiplicities were described in standard abbreviations as singlet (s), doublet (d), doublet of doublets (dd), doublet of doublet of doublets (ddd), triplet (t), broad singlet (bs), or multiplet (m). Organic solutions were concentrated under vacuum at <40 °C.

*N*-Acetylneuraminic acid, Neu5Ac (5-acetamido-3,5-dideoxy-d-*glycero*-d-galacto-non-2-ulosonic acid) was purchased from Carbosynth UK. Methyl 5-acetamido-4,7,8,9-tetra-*O*-acetyl-2,6-anhydro-3,5-dideoxy-d-*glycero*-d-galacto-non-2-enonate (1), was synthesized according to literature procedure.^42^

### Methyl 7,8,9-tri-*O*-acetyl-2,3,4,5-tetradeoxy-2,3-didehydro-2,3-trideoxy-4′,5′-dihydro-2′-methyloxazolo[5,4-*d*]-d-*glycero*-d-talo-non-2-enonate (2)

Compound 1 was synthesized from commercially available *N*-acetyl neuraminic acid (Neu5Ac) as reported.^42^ Compound 1 (2.0 g, 3.7 mmol, 1 equiv.) was dissolved in anhydrous ethyl acetate (25 mL). The solution was warmed to 40 °C and TMSOTf (2.5 mL, 11.2 mmol, 3 equiv.) was added dropwise to the solution over 10–15 minutes. The resulting solution was stirred at 50 °C for 3 h under argon atmosphere. The progress of the reaction was monitored by TLC, and after completion, the solution was added to a vigorously stirred ice-cold solution of saturated aqueous sodium bicarbonate. The resulting solution was washed with EtOAc (200 mL × 2) and brine solution. The organic layer was collected, dried over anhydrous sodium sulfate, and concentrated under reduced pressure. The crude product was purified by chromatography on a silica gel (SiO_2_) column to provide the desired product 2 (1.2 g, 75%) as colorless solid. *R*_f_ = 0.41 (EtOAc/hexane = 1 : 1); ^1^H NMR (600 MHz, CDCl_3_) *δ* (ppm) 6.39 (d, *J* = 4.2 Hz, –C2–H, 1H), 5.64 (d, *J* = 6.0, 2.4 Hz, 1H), 5.44 (ddd, *J* = 9.0, 6.6, 3.0 Hz, 1H), 4.83 (dd, *J* = 8.4, 3.6 Hz, 1H), 4.61 (dd, *J* = 12.6, 2.4 Hz, 1H), 4.24 (dd, *J* = 12.6, 6.6 Hz, 1H), 3.96 (t, *J* = 9.0 Hz, 1H), 3.82 (s, –CO_2_Me, 3H), 3.44 (dd, *J* = 10.2, 3.0 Hz, 1H), 2.16 (s, 3H), 2.06 (s, 3H), 2.04 (s, 3H), 2.01 (s, 3H). ^13^C NMR (176 MHz, CDCl_3_) *δ* (ppm) 170.63, 169.79, 167.56, 161.87, 147.16, 107.55, 72.23, 70.29, 68.87, 62.07, 61.99, 52.52, 20.83, 20.76, 20.60, 14.16. HRMS (ESI-MS) calculated for *m*/*z* [M + H]^+^ calcd for C_18_H_24_NO_10_: 414.1395, found: 414.1403, [M + Na]^+^ calcd for C_18_H_23_NNaO_10_: 436.1214, found: 436.1213.

### Methyl-5-acetamido-4-azido-7,8,9-tri-*O*-acetyl-2,3,5-trideoxy-d-*glycero*-d-galacto-2-non-2-enopyranosate (3)

Oxazoline derivative 2 (1.2 g, 2.9 mmol) was dissolved in anhydrous *t*BuOH (20 mL) and to this solution was added TMSN_3_ (1.3 mL, 11.6 mmol, 4 equiv.) dropwise at room temperature under argon atmosphere. The solution was then warmed up and refluxed at 80 °C for overnight. After completion, the solvent was evaporated under reduced pressure, co-evaporated with toluene, and the crude product was purified by chromatography on a silica gel (SiO_2_) column to afford desired derivative 3 (1.1 g, 83%) as a white solid. *R*_f_ = 0.44 (EtOAc/hexane = 3 : 2); ^1^H NMR (500 MHz, CDCl_3_) *δ* (ppm) 6.00 (d, *J* = 3.0 Hz, –C2–H, 1H), 5.61 (d, *J* = 6.0 Hz, 1H), 5.47 (dd, *J* = 3.0, 2.4 Hz, 1H), 5.38–5.36 (m, 1H), 4.60 (dd, *J* = 12.0, 3.0 Hz, 1H), 4.58–4.55 (m, 1H), 4.23 (q, *J* = 6.0 Hz, 1H), 3.83 (s, –CO_2_Me, 3H), 2.16 (s, 3H), 2.09 (s, 3H), 2.07 (s, 3H), 2.03 (s, 3H). ^13^C NMR (125 MHz, CDCl_3_) *δ* (ppm) 170.60, 170.45, 170.04, 161.52, 145.19, 107.45, 75.56, 70.56, 67.76, 61.96, 57.38, 52.63, 48.95, 23.40, 20.98, 20.80, 20.75. HRMS (ESI-MS) calculated for *m*/*z* [M + Na]^+^ calcd for C_18_H_24_N_4_NaO_10_: 479.1385, found: 479.1384.

### Methyl-5-acetamido-4-azido-7,8,9-tri-*O*-acetyl-3_eq_-bromo-3,5-dideoxy-*β*-d-*erythro*-l-manno-2-nonulopyranosonate (4) and methyl-5-acetamido-4-azido-7,8,9-tri-*O*-acetyl-3_ax_-bromo-3,5-dideoxy-*β*-d-*erythro*-l-manno-2-nonulopyranosonate (5)

To a solution of compound 3 (1.1 g, 2.4 mmol, 1 equiv.) in a mixture of acetonitrile–water (9 : 1, v/v), *N*-bromo succinimide (NBS) (0.53 g, 3.0 mmol, 1.25 equiv.) was added at room temperature. The solution was then warmed up and refluxed with stirring at 80 °C for 1 h. The progress of the bromohydroxylation was monitored by TLC using ethyl acetate–hexane–CH_2_Cl_2_ (1 : 4 : 1, v/v/v), TLC confirmed formation of bromohydrins 4 and 5 after 1 h. After completion, solvents were removed under reduced pressure. The crude mixture was dissolved in CH_2_Cl_2_ (100 mL), co-evaporated with toluene, and the crude product was dry loaded on a silica gel (SiO_2_) column and purified by chromatography using hexane-ethyl acetate, gradient elution from 1 : 1 to 1 : 9 to afford stereo-isomeric mixture of bromohydrins 4 and 5 (1.2 g, 92% combined yield) in a ratio of 3 : 7 respectively, as an off-white solid. *R*_f_ = 0.44 (EtOAc/hexane = 3 : 2); compound 4: ^1^H NMR (700 MHz, CD_3_OD) *δ* (ppm) 5.35 (dd, *J* = 6.0, 2.4 Hz, 1H), 5.09 (td, *J* = 6.3, 2.6 Hz, 1H), 4.48–4.33 (m, 2H), 4.17 (d, *J* = 10.9 Hz, H-3_axial_, 1H), 4.01–3.99 (m, 2H), 3.85 (s, –CO_2_Me, 3H), 2.69 (s, 3H), 2.09 (s, 3H), 2.05 (s, 3H), 1.99 (s, 3H), 1.92 (s, 3H). ^13^C NMR (125 MHz, CDCl_3_) *δ* (ppm) 170.77, 170.65, 170.63, 170.50, 167.74, 95.18, 70.64, 69.98, 67.79, 64.84, 62.40, 60.46, 54.29, 51.23, 50.51, 23.41, 23.19, 21.06, 20, 20.80, 20.74. HRMS (ESI-MS) calculated for *m*/*z* [M + Na]^+^ calcd for C_18_H_25_BrN_4_NaO_11_: 575.0595, found: 575.0591. Compound 5: ^1^H NMR (700 MHz, CD_3_OD) *δ* (ppm) 5.38 (dd, *J* = 4.9, 2.1 Hz, 1H), 5.24 (ddd, *J* = 7.4, 4.9, 2.5 Hz, 1H), 4.69 (dd, *J* = 12.0, 2.5 Hz, 1H), 4.56 (d, *J* = 3.0 Hz, H-3_equatorial_, 1H), 4.40–4.32 (m, 2H), 4.19–4.15 (m, 1H), 3.78 (s, –CO_2_Me, 3H), 2.11 (s, 3H), 2.02 (s, 3H), 2.01 (s, 3H), 1.91 (s, 3H). ^13^C NMR (125 MHz, CD_3_OD) *δ* (ppm) 173.50, 172.50, 171.95, 171.85, 169.11, 96.89, 72.86, 72.28, 69.62, 63.78, 61.16, 54.78, 53.21, 22.77, 20.85, 20.82, 20.66, 14.17. HRMS (ESI-MS) calculated for *m*/*z* [M + Na]^+^ calcd for C_18_H_25_BrN_4_NaO_11_: 575.0595, found: 575.0593.

### Methyl (5-acetamido-4-azido-2,7,8,9-tetra-*O*-acetyl-3,4,5-trideoxy-d-*glycero*-α/β-d-galacto-2-nonulopyranosid)onate (6)

To a solution of mixed bromohydrins 4 and 5 (607 mg, 1.1 mmol, 1 equiv.) in anhydrous THF (12 mL), *n*Bu_3_SnH (1.2 mL, 4.4 mmol, 4 equiv.) was added followed by catalytic amount of AIBN (10% mmol) as a free-radical initiator at room temperature. The mixture was warmed up and refluxed at 70 °C for 1 h under argon atmosphere. Reaction times of ≤1 h ensured selective debromination, where prolonged reaction times resulted in the reduction of the azide group to amine. Progress of the reaction was monitored closely by TLC. After completion, the solvent was removed under reduced pressure, and the residue was dissolved in CH_3_CN (50 mL), and washed with hexane (30 mL × 3) repeatedly. The acetonitrile was then removed under reduced pressure. The crude residue was purified by chromatography on a silica gel (SiO_2_) column using hexane-ethyl acetate, gradient elution from 1 : 1 to 1 : 9 provided the C2-hydroxyl derivative (251 mg, 48%) as a white powder. This was followed by acetylation of the C2-hydroxyl derivative (251 mg, 0.53 mmol) in anhydrous pyridine (3 mL), and anhydrous acetic anhydride (3 mL) was added under ice-bath at 0 °C, warmed up to room temperature, and stirred overnight. The progress of the reaction was monitored by TLC. After completion, solvents were removed under reduced pressure, and the crude product was co-evaporated with toluene. The crude product was purified by chromatography on a silica gel (SiO_2_) column using hexane-ethyl acetate, gradient elution from 1 : 0 to 9 : 1 to afford desired per-*O*-acetylated compound 6 (270 mg, quantitative) as a colorless solid. *R*_f_ = 0.39 (EtOAc/hexane = 3 : 2); ^1^H NMR (700 MHz, CDCl_3_) *δ* (ppm) 5.68 (d, *J* = 8.4 Hz, 1H), 5.33 (dd, *J* = 6.0, 2.0 Hz, 1H), 5.10 (td, *J* = 6.0, 2.4 Hz, 1H), 4.44 (dd, *J* = 12.5, 2.5 Hz, 1H), 4.40–4.34 (m, 2H), 4.16 (dd, *J* = 12.5, 6.1 Hz, 1H), 3.77 (s, 3H), 3.35 (d, *J* = 10.6 Hz, 1H), 2.54 (dd, *J* = 13.7, 4.8 Hz, 1H), 2.14 (s, 6H), 2.04 (s, 3H), 2.02 (s, 3H), 2.00 (s, 3H), 1.83 (dd, *J* = 13.7, 12.0 Hz, 1H). ^13^C NMR (176 MHz, CDCl_3_) *δ* (ppm) 170.84, 170.83, 170.53, 169.93, 168.17, 166.43, 96.85, 70.62, 68.19, 61.98, 60.37, 55.90, 53.18, 36.29, 23.47, 20.82, 20.71, 20.67, 14.17. HRMS (ESI-MS) calculated for *m*/*z* [M + Na]^+^ calcd for C_20_H_28_N_4_NaO_12_: 539.1596, found: 539.1592.

### Methyl-5-azidoacetamido-2,4,7,8,9-penta-*O*-acetyl-3,5-trideoxy-*β*-d-*erythro*-l-manno-2-nonulopyranosonate (7)

Dowex H^+^ resin was washed with MeOH three times and dried before adding 100 mg resin to a solution of *N*-azidoacetyl-3,5-dideoxy-d-*erythro*-α-l-manno-2-nonulopyranosonate^43^ (200 mg, 0.57 mmol) in 10 mL MeOH. After stirring the reaction mixture overnight at room temperature, resin was filtered off by cotton filtration. The filtrate was concentrated *in vacuo* affording methyl ester intermediate as a white solid which was used for next reaction without further purification. A solution of the methyl ester intermediate in pyridine (3 mL) was taken in a round bottom flask, and Ac_2_O (3 mL) was slowly added at 0 °C. After stirring at RT for 12 h, the mixture was quenched with MeOH and concentrated *in vacuo* with toluene co-evaporation. The residue was purified by silica column chromatography using EtOAc : hexane (70 : 30, v/v) to afford 7 as a white solid (279 mg, 85%); ^1^H NMR (600 MHz, CDCl_3_) *δ* (ppm) 6.30 (d, *J* = 10.0 Hz, 1H), 5.39 (dd, *J* = 5.4, 2.4 Hz, 1H), 5.30 (ddd, *J* = 11.2, 10.8, 4.8 Hz, 1H), 5.10 (ddd, *J* = 8.4, 6.0, 2.4 Hz, 1H), 4.22 (dd, *J* = 10.6, 2.4 Hz, 1H), 3.91–3.83 (m, 2H), 3.81 (s, 3H), 2.59 (dd, *J* = 13.8, 4.8 Hz, 1H), 2.17 (s, 3H), 2.14 (s, 3H), 2.10–2.09 (m, 1H), 2.07 (s, 3H), 2.04 (s, 3H), 2.03 (s, 3H). ^13^C NMR (125 MHz, CDCl_3_) *δ* (ppm) 171.15, 170.70, 170.59, 170.34, 168.20, 167.28, 166.19 97.53, 72.46, 70.97, 68.08, 67.59, 61.92, 53.22, 52.61, 49.21, 35.90, 21.04, 20.86, 20.76, 20.75. HRMS (ESI-MS) calculated for *m*/*z* [M + Na]^+^ calcd for C_22_H_30_N_4_O_14_: 574.4922, found: 574.4920.

### Methyl (5-acetamido-4-azido-2-chloro-7,8,9-tri-*O*-acetyl-3,4,5-trideoxy-d-*glycero*-β-d-galacto-2-nonulopyranosid) onate (8)

Compound 6 (267 mg, 0.51 mmol) was dissolved in AcCl (10 mL) and was cooled with ice-bath to 0 °C. Freshly prepared HCl gas was passed through the solution and the reaction mixture was stirred under HCl gas overnight at room temperature by following a method reported by Daskhan and co-workers.^44^ After completion, the solvent was removed *in vacuo*, co-evaporated with toluene (50 mL × 2), and the acidic residue was purified by rapid silica gel (SiO_2_) column chromatography to minimize hydrolysis and afforded 8 (230 mg, 90%) as a white solid. *R*_f_ = 0.33 (EtOAc/hexane = 9 : 1); ^1^H NMR (500 MHz, CDCl_3_) *δ* (ppm) 5.61 (d, *J* = 9.0 Hz, 1H), 5.47 (dd, *J* = 7.0, 2.0 Hz, 1H), 5.22–5.19 (m, 1H), 4.54 (dd, *J* = 10.5, 1.5 Hz, 1H), 4.42 (dd, *J* = 12.5, 2.5 Hz, 1H), 4.27 (ddd, *J* = 17.0, 10.5, 7.0, Hz, 1H), 4.12 (q, *J* = 5.5 Hz, 1H), 3.90 (s, 3H), 3.74 (dd, *J* = 13.5, 4.7 Hz, 1H), 2.82 (q, *J* = 4.5 Hz, 1H), 2.15 (s, 3H), 2.12 (dd, *J* = 14.0, 12.6 Hz, 1H), 2.09 (s, 3H), 2.07 (s, 3H), 2.04 (s, 3H). HRMS (ESI-MS) calculated for *m*/*z* [M + H]^+^ calcd for C_18_H_26_ClN_4_O_10_: 493.1332, found: 493.1336, [M + Na]^+^ calcd for C_18_H_25_ClN_4_NaO_10_: 515.1151, found: 515.1150.

### Methyl (*p*-tolyl 5-acetamido-4-azido-7,8,9-tri-*O*-acetyl-3,4,5-trideoxy-2-thio-α-d-*glycero*-d-galacto-2-nonulopyranosid)onate (9)

To a solution of compound 8 (230 mg, 0.46 mmol, 1 equiv.) in anhydrous CH_2_Cl_2_ (4 mL), *p*-toluenethiol (113 mg, 0.58 mmol, 1.25 equiv.) was added at room temperature. *N*,*N*-Diisopropylethylamine, DIPEA, (70 mL, 1.5 equiv.) was added to the reaction mixture and the solution was then stirred for overnight under argon atmosphere. The reaction was monitored by TLC and after completion, the solvent was removed under reduced pressure. The crude product was diluted with CH_2_Cl_2_ (100 mL), washed with water (50 mL × 2) and brine solution, dried over sodium sulphate, and concentrated under reduced pressure. The product was purified by chromatography to give the desired product 9 (248 mg, 91%) as a white solid. ^1^H NMR (600 MHz, CDCl_3_) *δ* (ppm) 7.39 (d, *J* = 7.8 Hz, STol, 2H), 7.15 (d, *J* = 7.8 Hz, STol, 2H), 5.51 (d, *J* = 8.4 Hz, N*H*, 1H), 5.30–5.25 (m, 2H), 4.41 (dd, *J* = 12.6, 2.4 Hz, 1H), 4.29 (dd, *J* = 12.6, 4.8 Hz, 1H), 4.14 (d, *J* = 10.2 Hz, 1H), 3.62 (s, 3H), 2.86 (d, *J* = 13.2, 4.8 Hz, 1H), 2.38 (s, 3H), 2.18 (s, 3H), 2.07 (s, 3H), 2.06 (s, 3H), 1.99 (s, 3H), 1.77 (dd, *J* = 14.0, 12.61 Hz, 1H). ^13^C NMR (176 MHz, CDCl_3_) *δ* (ppm) 170.75, 170.83, 170.62, 170.57, 169.99, 167.91, 140.31, 136.52, 129.64, 124.99, 87.28, 73.25, 69.80, 68.20, 61.91, 57.80, 52.76, 51.86, 30.30, 23.51, 21.35, 20.96, 20.87, 20.78. HRMS (ESI-MS) calculated for *m*/*z* [M + Na]^+^ calcd for C_25_H_32_N_4_NaO_10_S: 603.1731, found: 603.1732.

### 5-Acetamido-4-azido-2-*p*-tolyl-3,4,5-trideoxy-2-thio-d-*glycero*-α-d-galacto-nonulopyranosic acid (10)

Compound 9 (248 mg, 0.42 mmol) was dissolved in NaOMe/MeOH (20 mL, pH 9) and the reaction mixture was stirred at room temperature under argon atmosphere overnight. The solution was concentrated under reduced pressure and the residue was taken to next step without any further purification. The crude product was dissolved in 1 M aqueous NaOH (1 mL) and the solution was then stirred at room temperature overnight. The reaction mixture was then neutralized with amberlite IR-120 resin (H^+^) and filtered, the aqueous solution was washed with Et_2_O (20 mL × 4 mL) and freeze dried by lyophilization to afford desired derivative 10 (175 mg, 93%) as a white solid. ^1^H NMR (600 MHz, D_2_O) *δ* (ppm) 7.43 (d, *J* = 7.8 Hz, STol, 2H), 7.21 (d, *J* = 7.8 Hz, STol, 2H), 3.79 (t, *J* = 12.0, 2.4 Hz, 1H), 3.77 (dd, *J* = 10.2, 2.4 Hz, 1H), 3.75–3.73 (m, 1H), 3.58–3.52 (m, 4H), 2.85 (dd, *J* = 13.2, 4.8 Hz, 1H), 2.31 (s, 3H, *Me*-STol), 1.98 (s, 3H, NHAc), 1.83 (t, *J* = 12.6 Hz, 1H). ^13^C NMR (176 MHz, D_2_O) *δ* (ppm) 177.18, 174.09, 169.99, 167.91, 140.31, 136.52, 129.64, 124.99, 96.93, 71.99, 71.95, 69.96, 64.82, 59.90, 51.73, 49.67, 38.79, 22.94. HRMS (ESI-MS) calculated for *m*/*z* [M − H]^−^ calcd for C_18_H_24_N_4_O_7_S: 439.1731, found: 439.1732.

### 5-Acetamido-4-azido-3,4,5-trideoxy-d-*glycero*-α-d-galacto-nonulopyranosic acid (11)

To a solution of thiosialoside compound 10 (150 mg, 0.34 mmol) in H_2_O (4 mL), iodine (354 mg, 1.36 mmol, 4 equiv.) was added at room temperature. The solution was stirred under dark. The progress of the reaction was monitored by TLC. After completion, the solution was filtered through Celite pad. Excess dissolved iodine was quenched by aqueous saturated solution of sodium thiosulfate, and the aqueous solution was freeze-dried and lyophilized. The crude product was purified by C-18 Sep-pak chromatography using gradient elution of H_2_O to MeOH/H_2_O (1 : 9 to 8 : 2 v/v) to provide compound 11 (100 mg, 84%) as a colorless powder after lyophilization. ^1^H NMR (500 MHz, CD_3_OD) *δ* (ppm) 4.09–4.02 (m, 2H), 3.95–3.90 (m, 1H), 3.78–3.74 (m, 1H), 3.67–3.59 (m, 2H), 3.51–3.49 (m, 1H), 2.13 (dd, *J* = 12.5, 4.5 Hz, 1H), 2.01 (s, 3H, NHAc), 1.85 (t, *J* = 12.5 Hz, 1H). ^13^C NMR (125 MHz, CD_3_OD) *δ* (ppm) 177.18, 174.09, 96.93, 71.99, 71.95, 69.96, 64.82, 59.90, 51.73, 49.67, 22.94. HRMS (ESI-MS) calculated for *m*/*z* [M − H]^−^ calcd for C_11_H_17_N_4_O_8_: 333.1052, found: 333.1051.

### Enzymatic synthesis of 2-(*N*-benzyloxycarbonylamino)ethyl 5-acetamido-4-azido-3,4,5 trideoxy-d-*glycero*-α-d-galacto-nonulopyranosyl-(2–3)-[β-d-galactopyranosyl]-(1–3)-2 acetamido-2-deoxy-α-d-galactopyranoside (13) and 2-(*N*-benzyloxycarbonylamino)ethyl 5 acetamido-3,5-dideoxy-d-*glycero*-α-d-galacto-nonulopyranosyl-(2–3)-[(β-d-galactopyranosyl)]-(1–3)-2-acetamido-2-deoxy-α-d-galactopyranoside (14)

Disaccharide β-Gal*p*-(1→3)-α-Gal*p*NAc acceptor with (α-*N*-9benzyloxycarbonyl) amino-ethyl linker 12 (10 mM), 4-azido Neu5Ac donor 11 or Neu5Ac donor (2.0 equiv.) and CTP (2.6 equiv.) were dissolved in HEPES buffer (100 mM, pH 8.5) with 2M MgSO_4_. *Neisseria meningitides* CMP-sialic acid synthetase NmCSS, (final conc. = 0.2 μg mol^−1^) and human ST3GAL1 (final conc. = 5 μg μmol^−1^) were added to the reaction mixture followed by incubation at 37 °C in an isotherm incubator at 120 rpm for 3 h. The progress of the reaction was monitored by TLC using iprOH/NH_4_OH/H_2_O/ethyl acetate = 9 : 2 : 1 : 0.5 (by volume) as the solvent system and stained with *p*-anisaldehyde stain solution. The reaction was quenched by the addition of equal volume of ice-cold EtOH followed by incubation at 4 °C for 20 min. The supernatant was collected after centrifugation and concentrated by rotary evaporation. Purification was done by loading the supernatant into a Bio-gel P-2 gel filtration column with water as the eluant and C-18 Sep-pak chromatography using gradient elution of H_2_O to MeOH/H_2_O (1 : 9 to 8 : 2 v/v) to provide sialosides α-4AzNeu*p*5Ac-(2→3)-β-Gal*p*-(1→3)-α-Gal*p*NAc 13 (1.0 mg, 40%) and α-Neu*p*5Ac-(2→3)-β-Gal*p*-(1→3)-α-Gal*p*NAc 14 (2.6 mg, 60%), from 11 and Neu5Ac respectively, as white powders after lyophilization. Compound 13: ^1^H NMR (500 MHz, D_2_O) *δ* (ppm) 7.52–7.48 (m, 5H), 5.24 (d, *J* = 12.2 Hz, 1H), 5.17 (d, *J* = 12.5 Hz, 1H), 4.93 (d, *J* = 3.2 Hz, 1H), 4.54 (d, *J* = 7.6 Hz, 1H), 4.39–4.36 (m, 1H), 4.23 (d, *J* = 2.3 Hz, 1H), 4.13 (dd, *J* = 10.0, 1.9 Hz, 1H), 4.06–3.89 (m, 6H), 3.83–3.57 (m, 12H), 3.50 (ddd, *J* = 14.6, 6.9, 3.6 Hz, 1H), 3.41–3.36 (m, 1H), 2.83 (dd, *J* = 12.8, 4.5 Hz, 1H), 2.10 (s, 3H), 2.03 (s, 3H), 1.83 (t, *J* = 12.7 Hz, 1H). ^13^C NMR (125 MHz, D_2_O) *δ* (ppm) 175.79, 175.48, 174.46, 159.45, 137.48, 129.79, 129.40, 128.59, 105.44, 100.54, 98.28, 78.38, 76.64, 75.69, 74.13, 72.77, 71.67, 69.97, 69.52, 68.90, 68.32, 67.82, 67.77, 63.44, 62.13, 61.86, 60.55, 50.77, 49.48, 41.19, 37.76, 23.01. HRMS (ESI-MS) calculated for *m*/*z* [M − H]^−^ calcd for C_35_H_51_N_6_O_20_: 875.3164, found: 875.3161. Compound 14: ^1^H NMR (500 MHz, D_2_O) *δ* (ppm) 7.47–7.42 (m, 5H), 5.18 (d, *J* = 12.3 Hz, 1H), 5.11 (d, *J* = 12.5 Hz, 1H), 4.87 (d, *J* = 3.2 Hz, 1H), 4.49 (d, *J* = 7.6 Hz, 1H), 4.32 (dd, *J* = 10.9, 3.5 Hz, 1H), 4.18 (d, *J* = 1.5 Hz, 1H), 4.07 (dd, *J* = 10.0, 1.9 Hz, 1H), 3.97 (dd, *J* = 11.5, 2.6 Hz, 1H), 3.94 (d, *J* = 2.6 Hz, 1H), 3.90–3.83 (m, 3H), 3.76–3.60 (m, 10H), 3.55–3.52 (m, 2H), 3.45 (ddd, *J* = 14.7, 7.0, 3.5 Hz, 1H), 3.36–3.30 (m, 1H), 2.77 (dd, *J* = 12.5, 4.6 Hz, 1H),2.04 (s, 3H), 1.97 (s, 3H), 1.79 (t, *J* = 12.7 Hz, 1H). ^13^C NMR (125 MHz, D_2_O) *δ* (ppm) 175.98, 175.48, 174.87, 159.45, 137.47, 129.79, 129.40, 128.59, 105.44, 100.66, 98.28, 78.38, 76.63, 75.71, 73.79, 72.80, 71.66, 70.00, 69.51, 69.36, 69.03, 68.29, 67.77, 63.47, 62.13, 61.87, 52.65, 49.49, 41.20, 40.75, 23.02. HRMS (ESI-MS) calculated for *m*/*z* [M − H]^−^ calcd for C_35_H_52_N_3_O_21_: 850.3099, found: 850.3097.

## Results

### Synthesis of peracetylated 4AzNeu5Ac (6)

**Scheme 1:**
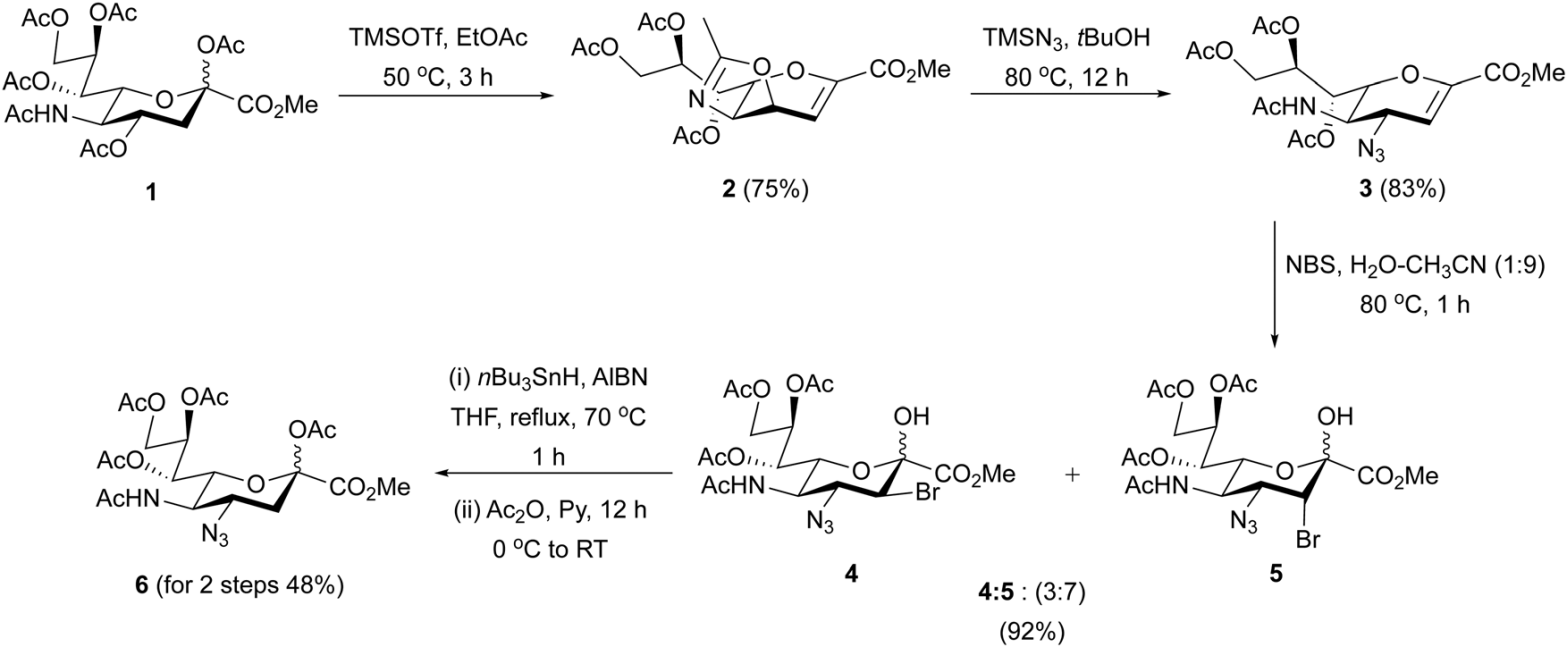
Synthesis of Peracetylated 4AzNeu5Ac 6.

Recently, it was reported that an azide can be directly installed onto Neu5Ac in a 45% yield after extended reaction times.^45^ However, attempts to reproduce this route using the published conditions were unsuccessful due to low yields (≤10%) over two-steps, resulting from several spots as observed on the TLC plate. Therefore, we synthesized peracetylated 4AzNeu5Ac 6 through a previously established route to generate bromohydrins 4 and 5 as a stereo-isomeric mixture in a ratio of 3 : 7 with a combined yield of 92%, followed by radical debromination (Scheme 1). Treatment of the mixture of bromohydrins with tri-*n*-butyltin hydride (Bu_3_SnH) in the presence of catalytic amount of azobisisobutyronitrile (AIBN) as a radical initiator under refluxing conditions in anhydrous THF under N_2_ atmosphere over 1 h gave the C2-hydroxyl intermediate. Short reaction time (≤1 h) at 70 °C selectively gave the C4-azide product with 48% yield. Increasing the temperature to 80 °C with prolonged reaction times of up to 3 hours resulted in the formation of C4-amine as reported by Boons and co-workers.^46^ This was followed by protection of the C2–OH group as an *O*-acetyl using Ac_2_O in pyridine overnight to afford peracetylated 4AzNeu5Ac 6 in quantitative yield.

### Evaluating peracetylated 4AzNeu5Ac (6) in MOE

**Fig. 1:**
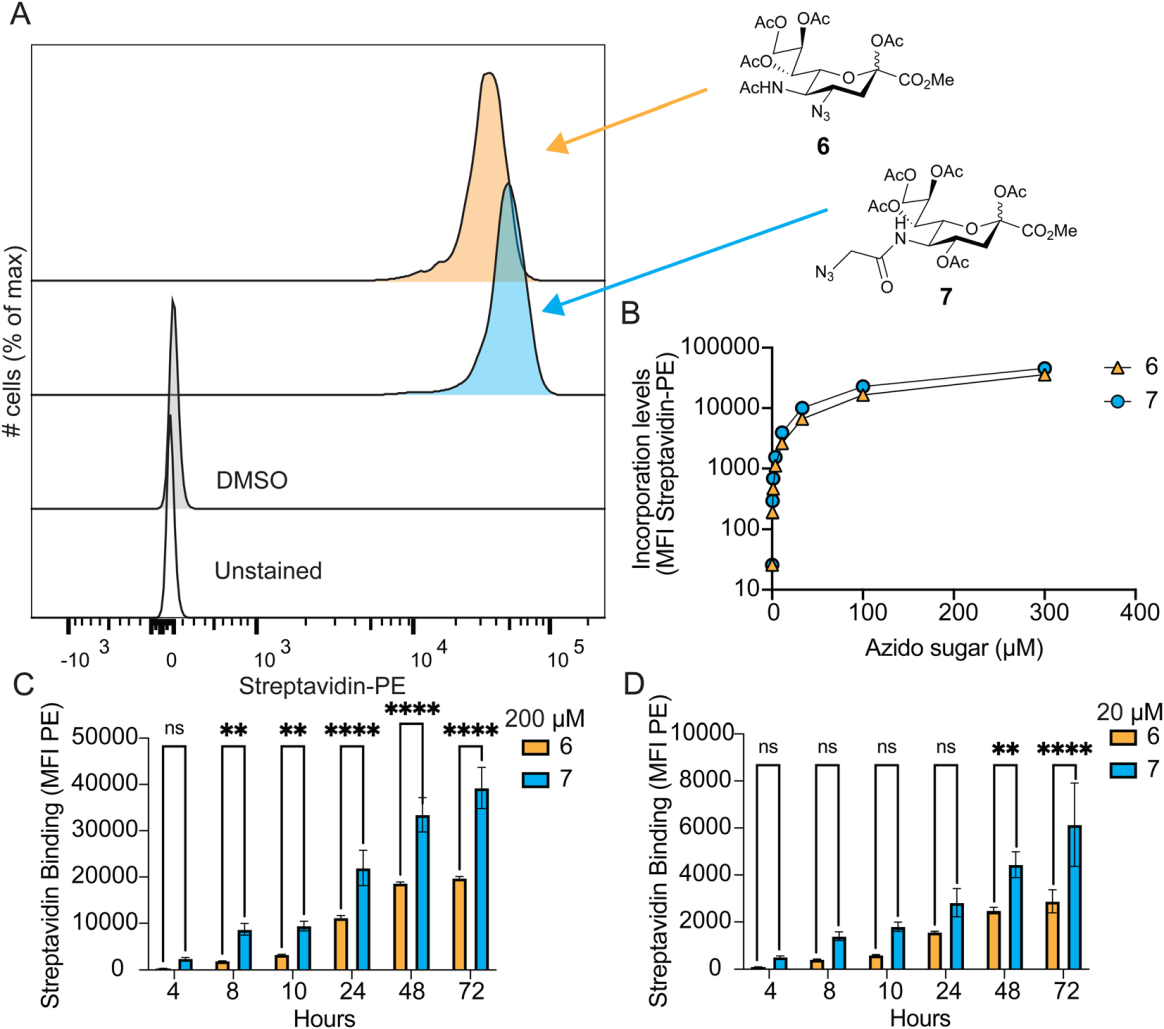
Incorporation of peracetylated 4AzNeu5Ac 6 & Neu5Az 7 into cellular glycans. (A) and (B) 4AzNeu5Ac 6 and Neu5Az 7 were fed to U937 cells for 3 days and detected through SPAAC-mediated attachment of biotin, recognition with fluorescent streptavidin, and detection by flow cytometry. (A) Representative flow cytometry histograms and (B) quantification of the flow cytometry data (*n* = 3 independent replicates). Error bars are not shown from small size. (C) and (D) 4AzNeu5Ac 6 and Neu5Az 7 were fed to U937 WT cells at 4, 8, 10, 34, 48, and 72 hour prior to SPAAC-mediated biotin attachment and fluorescent labelling for flow cytometry analysis at (C) 200 and (D) 20 μM sugars to determine maximum signal from time. Statistical analysis 2way Anova was performed. Not significant (ns), *P* > 0.05; **, *P* < 0.0068; ****, *P* < 0.0001.

**Fig. 2:**
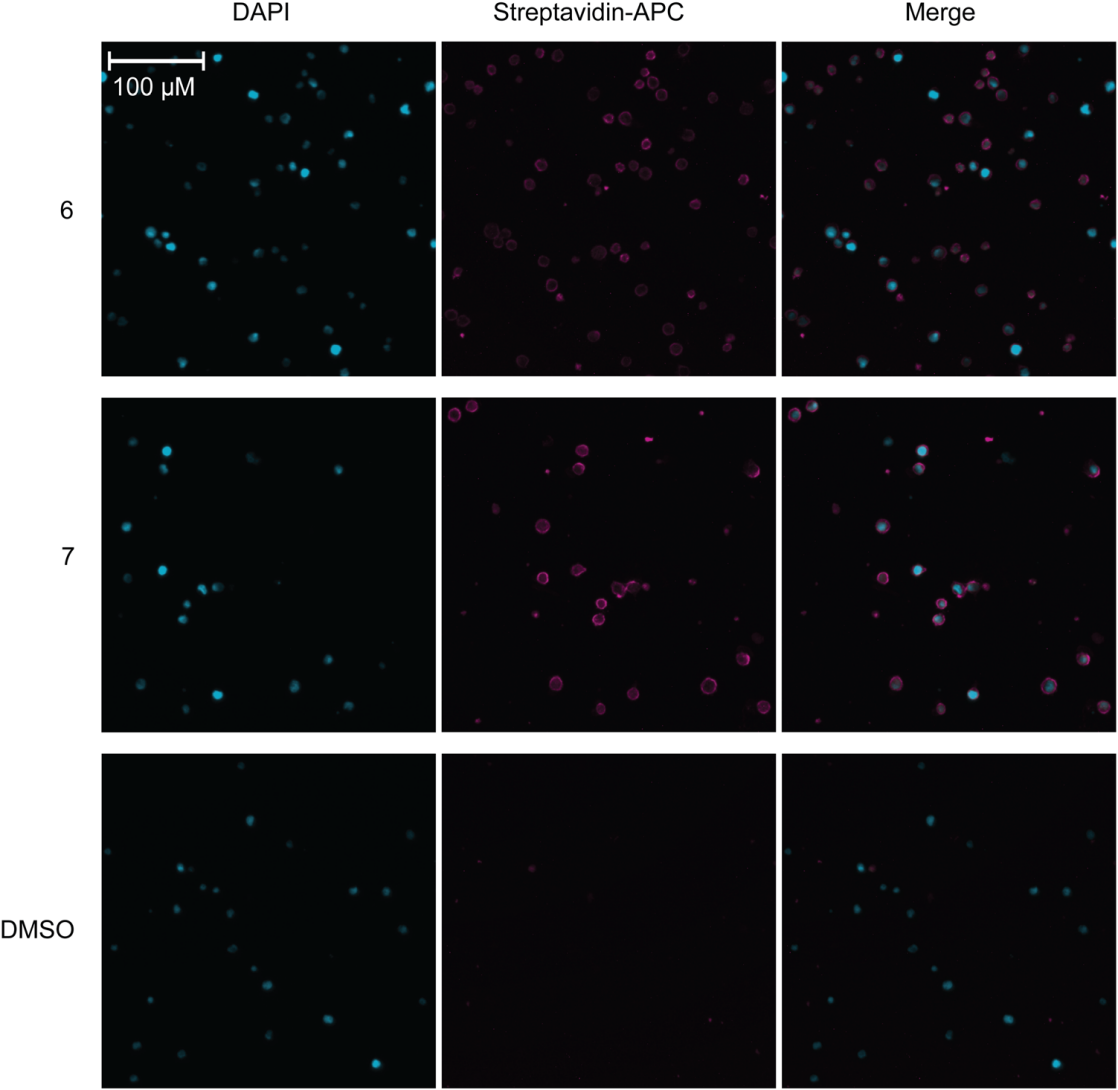
Visualizing 6 and 7 on the cell surface with cell imaging microscopy. Microscopy of U937 cells showing DAPI (blue, left) staining, streptavidin-APC (pink, middle) and merged (right). Cells were either fed 6 (top), 7 (middle), or DMSO control (bottom).

Access to peracetylated 4AzNeu5Ac 6 enabled it's testing in MOE, and we directly compared its efficacy to peracetylated Neu5Az 7. We performed a dose–response with U937 cells from 0.45–300 μM. After three days, SPAAC was performed with biotin–polyethylene glycol 4-dibenzylcyclooctyne (biotin–PEG4-DBCO), followed by fluorescent streptavidin labelling and detection by flow cytometry (Fig. 1A and B). The signal with peracetylated 4AzNeu5Ac 6 was 21% lower than peracetylated Neu5Az 7 at the highest dose (Fig. 1A) and 6 only incorporated moderately less across all concentrations tested when the mean fluorescence intensities were compared (Fig. 1B). U937 *CMAS*^−/−^ cells unable to make CMP-Neu5Ac were treated with 6 and 7 and showed only an extremely small signal with 6 and no signal above baseline for 7 (Fig. S1, ESI†), indicating that the large majority of signal from the WT cells is mostly attributed to metabolically-incorporated sialic acid on the cell surface. The very small amount of signal for 6 in *CMAS*^−/−^ cells could reflect non-specific labelling of cysteines.^47^ A time course of both 6 and 7 was performed at 20 and 200 μM, revealing that a maximum signal in flow cytometry was reached after 48 hours for both concentrations of 6 and 20 μM 7 as there was no statistically significant increase in signal at 72 h. A maximum signal for 200 μM 7 was achieved after 72 hours (Fig. 1C and D). Microscopy revealed that both compounds were observed to decorate the cell surface to similar extents (Fig. 2). These results demonstrate for the first time that peracetylated 4AzNeu5Ac 6 can be used in MOE.

### Synthesis of deprotected 4AzNeu5Ac (11)

**Scheme 2:**
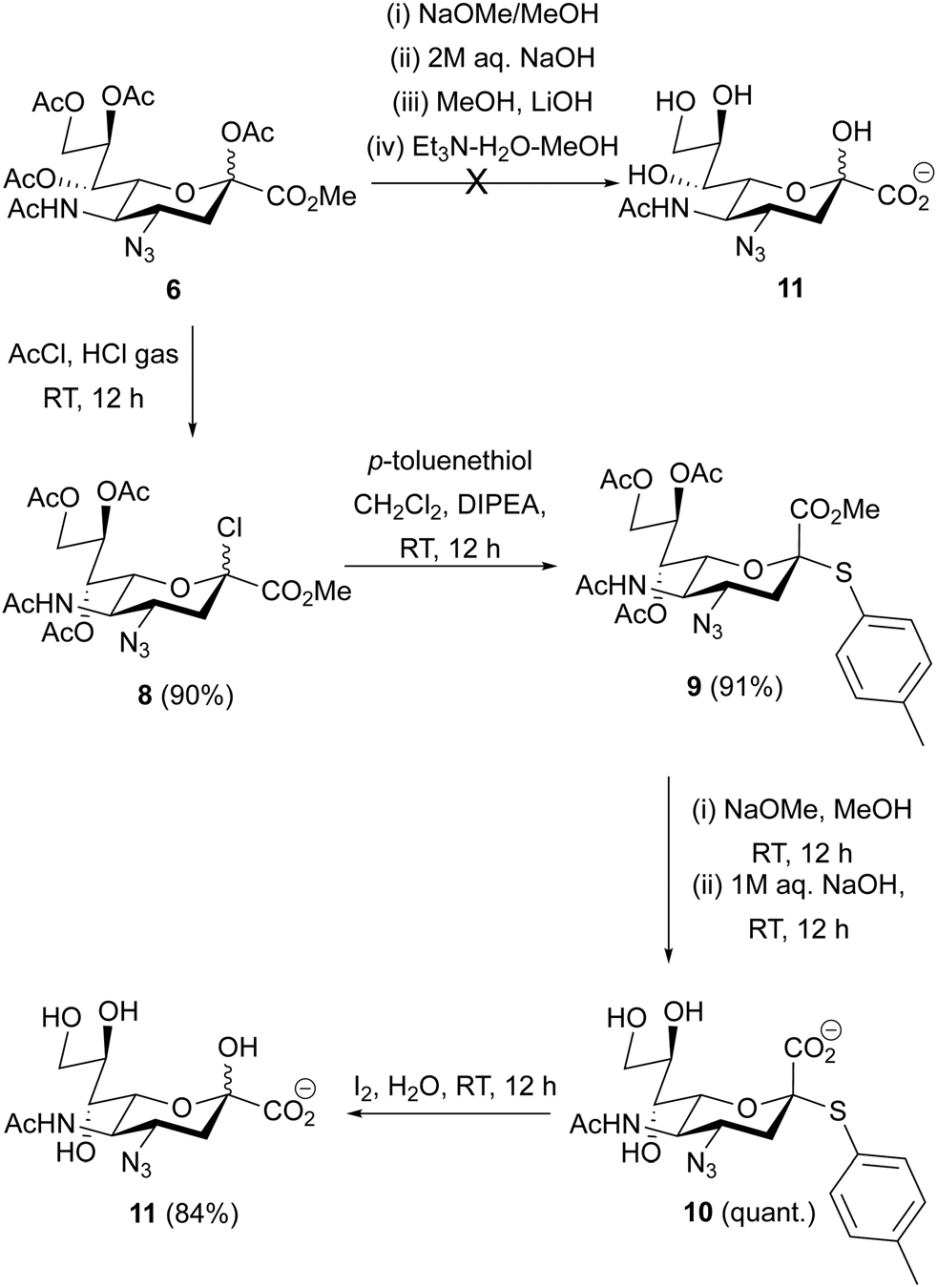
Deprotection of peracetylated 4AzNeu5Ac 6 to afford 11.

Given the ability of cells to incorporate 4AzNeu5Ac into cellular glycans, this suggests that mammalian STs can accommodate CMP-4AzNeu5Ac as their donor. Therefore, we aimed to make a trisaccharide with 4AzNeu5Ac using chemoenzymatic synthesis. For this, it required the deprotected form of 4AzNeu5Ac (11). Starting from 6, standard Zemplen deacetylation conditions with varying pH (8–11) were used but these only resulted in the formation of complex inseparable byproducts and failed to produce the desired product 11 (Scheme 2). Evidence suggested that alkaline degradation may have occurred, which is in line with the azide group proximal to the anomeric center facilitating a ‘peeling’ reaction.^37^ Similarly, heparan sulfate containing a 3-*O*-sulfate glucosamine on the reducing end has been discovered to undergo peeling reactions under mildly basic conditions.^48^ Milder basic conditions were also attempted but could not overcome this issue.

Therefore, we modified our synthetic route to have the anomeric hydroxyl group protected as a thioglycoside (Scheme 2). This was accomplished by treating 6 with acetyl chloride by bubbling in HCl gas at RT to afford the glycosyl chloride 8 in 90% yield.^44^8 was reacted with *p*-toluenethiol in DCM in the presence of DIPEA at RT overnight to afford the thioglycoside 9 in 91% yield after silica gel chromatography. De-*O*-acetylation of 9, using a catalytic amount of NaOMe in methanol at ambient temperature, followed by hydrolysis of the methyl ester with 1M aqueous NaOH afforded compound 10 in quantitative yield over two-steps. Iodine-mediated hydrolysis of the thiotoluene in water at RT afforded 4AzNeu5Ac 11 in 84% yield.

### Chemoenzymatic synthesis of a 4AzNeu5Ac sialoside

**Scheme 3:**
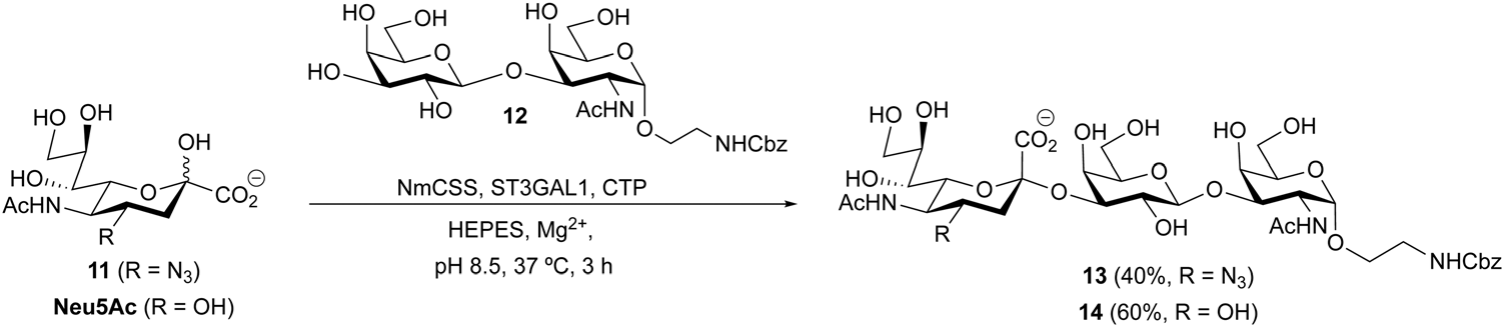
Chemoenzymatic synthesis of T-antigen sialoside 14 and its 4Az analog 13.

Siglec-7 is considered a glyco-immune checkpoint that plays an important role in cancer.^49^ Recent findings have converged on Siglec-7 ligands being on sialylated core-1 mucin-type *O*-glycans.^50^ ST3GAL1/2 has been shown to be essential in creating *O*-glycans that are ligands of Siglec-7 but, interestingly, the affinity of sialyl T for Siglec-7 has never been measured. Motivated to test if Siglec-7 can recognize a 4-modified sialoside, we aimed to use deprotected 4AzNeu5Ac 11 in a one-pot two-enzyme chemoenzymatic reaction to install 11 onto the T-antigen (β-Gal*p*-(1→3)-α-Gal*p*NAc) containing an α-(*N*-benzyloxycarbonyl) amino-ethyl aglycone. (Scheme 3). Accordingly, 4AzNeu5Ac 11 was combined with CTP and *Neisseria meningitides* (CMP-sialic acid synthetase, NmCSS) for conversion to CMP-4AzNeu5Ac, and this donor was transferred to the β-Gal*p*-(1→3)-α-Gal*p*NAc acceptor 12 using recombinant human ST3GAL1 to obtain the desired trisaccharide 13. For comparison, we also synthesized the corresponding trisaccharide from Neu5Ac without the 4Az group 14 using similar reaction conditions.

### Assessing the affinity of sialosides 13 and 14 with Siglec-7

**Fig. 3:**
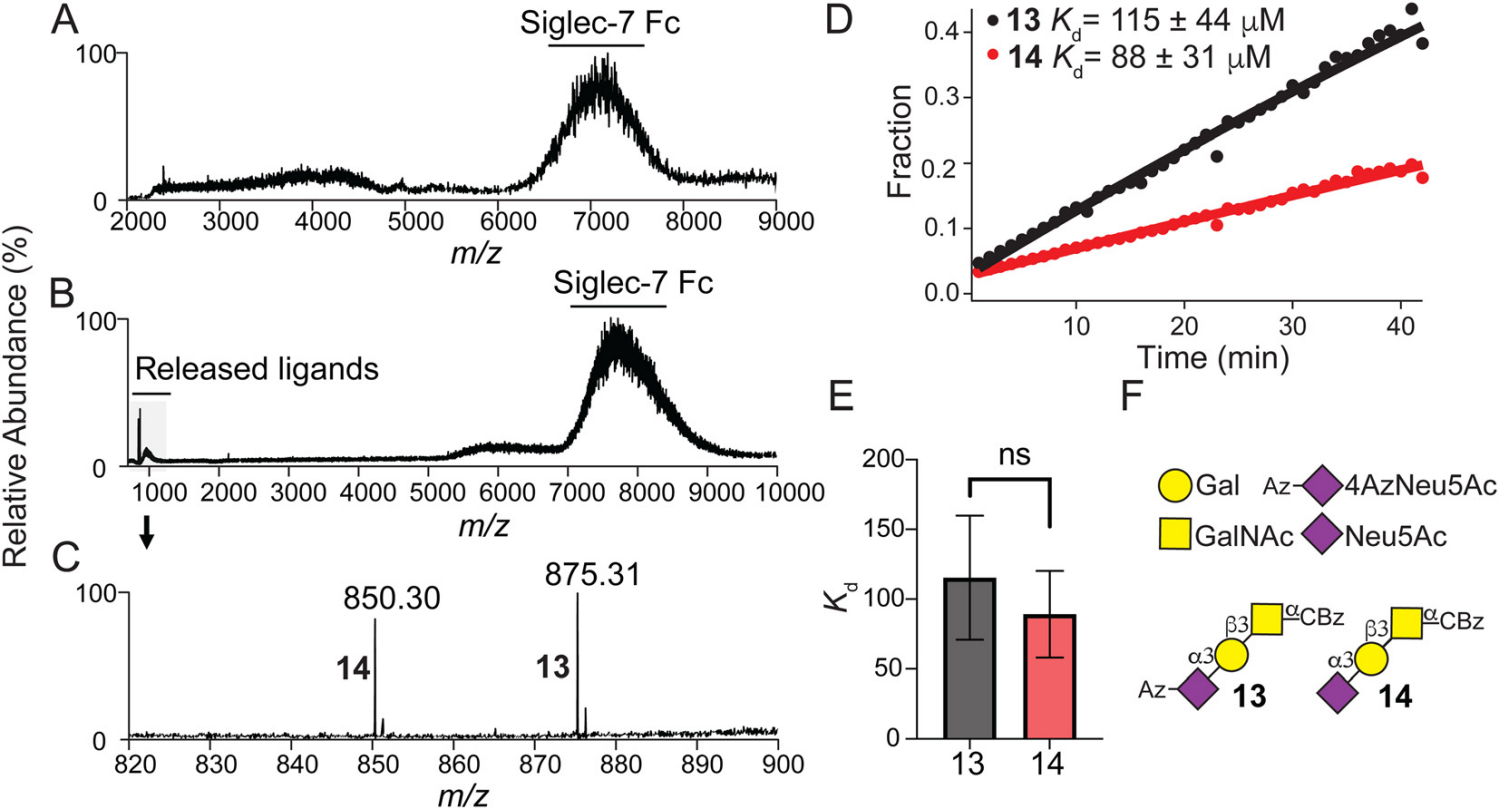
Siglec-7 Fc affinity measurements with 13 and 14. (A) Representative electrospray ionization (ESI) mass spectra acquired in negative ion mode for ammonium acetate solutions (200 mM, pH 7.4) of Siglec-7 Fc (0.5 μM). (B) Representative higher energy collision-induced dissociation (HCD) mass spectrum for ammonium acetate solutions (200 mM, pH 7.4) of Siglec-7 Fc (0.5 μM) with 13 and 14 (500 nM each), *m*/*z* range of 6000–9000 was isolated and a collision energy of 120 V was applied to release the ligands from the complexes with Siglec-7 Fc. (C) Deprotonated ions of ligands 13 and 14 produced in (B) with *m*/*z* 820–900. (D) Representative time-resolved relative abundance of released 13 and 14 ions normalized to total Siglec-7 Fc signal for COIN-CaR-ESI-MS experiments performed in ammonium acetate solutions (200 mM, pH 7.4) of Siglec-7 Fc (0.5 μM) and 13 and 14 (500 nM each) (Solution 1) and Siglec-7 Fc (0.5 μM) and 13 and 14 (40 μM each) (Solution 2). Solid curves represent the best fit of eq. to the experimental data. (E) Measured *K*_d_ values for 13 (*n* = 9) and 14 (*n* = 6) determined. Two-tailed Student's paired *t*-test was used for statistical analysis. Not significant (NS), *P* > 0.05. (F) SNFG nomenclature and symbols for sialosides 13 and 14.

Using recombinant Siglec-7-Fc soluble protein^51–53^ and a recently introduced concentration independent-native mass spectrometry (COIN-nMS) based method to measure the affinity of glycan ligands for glycan-binding proteins^54,55^ (see ESI,† for detailed method), we assessed the affinity of α-Neu5Ac-(2→3)-β-Gal*p*-(1→3)-α-Gal*p*NAc with (α-*N*-benzyloxycarbonyl) amino-ethyl linker 14 and it's corresponding 4Az derivative 13 for Siglec-7 (Fig. 3). The mass spectrum for Siglec-7 (Fig. 3A) shows affinity to both 13 and 14 as evidenced by the shift in *m*/*z* (Fig. 3B) corresponding to the *m*/*z* of the ligands (Fig. 3C). Time-resolved relative abundance of released ligands ions normalized to total Siglec-7 Fc signal (Fig. 3D, see ESI,† for Equations) determined average *K*_d_ values for 13 and 14 (Fig. 3E) for Siglec-7 *K*_d_ = 115 ± 44 μM and *K*_d_ = 88 ± 31 μM, respectively, indicating that a C4-Azido modified Neu5Ac does not significantly perturb binding.

## Discussion

A general approach to synthesize C4-modified Neu5Ac derivatives is through the introduction of nitrogen into an oxazoline derivative of Neu5Ac2en *via* stereoselective nucleophilic attack by azide.^56,57^ The key intermediate, peracetylated 4-azido Neu5Ac2en is then bromohydroxylated with treatment of NBS to form a bromoazide Neu5Ac derivative. Simultaneous debromination and reduction of the bromoazide using *n*Bu_3_SnH and AIBN will give 4-amino Neu5Ac used for subsequent *N*-acylation reactions to generate *N*-acetamido derivatives off the C-4 position of Neu5Ac.^21,58,59^ Another strategy uses direct 4-amination using cyclic secondary amines from peracetylated Neu5Ac in one step.^60^ These strategies allowed the derivatizations of the C4 position of Neu5Ac directly through the 4-amino group without accessing peracetylated 4AzNeu5Ac. To date, peracetylated 4-azido Neu5Ac has only been synthesized using a direct sialic acid 4-OAc substitution from the peracetylated Neu5Ac. In 2022, it was reported that sialic acid 4-OAc can be directly substituted by an azide group in three steps from Neu5Ac. This route required extended reaction times and a free C2-hydroxyl group on the sialic acid to afford the peracetylated 4AzNeu5Ac 6 in 45% yield.^45^ However, after several attempts to perform this transformation using the published conditions, we found that this route is inefficient giving only trace amounts of the product even at prolonged reaction times. We therefore synthesized peracetylated 4AzNeu5Ac 6 through a previously established route to generate mixtures of bromohydrins 4 and 5 followed by selective radical debromination using *n*Bu_3_SnH and catalytic amount of AIBN to give C2-hydroxyl intermediate of compound 6.^59,61^ It is important to note that in order to keep the C4-azide group intact, shorter reaction times at 70 °C were employed, which allowed selective debromination to give the desired product. On the other hand, longer reaction times and higher temperature can lead to the formation of C4-amine as reported by Boons and co-workers.^46^

A number of Neu5Ac and ManNAc derivatives have been developed for MOE applications, including those with Azides at C5, C7, and C9. Structural studies with mammalian studies^62^ and previously enzymatic assays^43,63^ have universally established that functional groups appended of the 5-acetamido of Neu5Ac are well tolerated by mammalian STs. Therefore, it is somewhat unsurprising that Neu5Az incorporates very well in MOE studies. However, it is noteworthy that the derivative needs to be tolerated by CMP-sialic acid synthetase and the Golgi CMP-Neu5Ac transporter. Our findings reveal that 4AzNeu5Ac worked nearly as well as Neu5Az in MOE, indicating that it is well tolerated by the biosynthetic machinery and STs. The small but significant decrease in incorporation of 4AzNeu5Ac could be attributed to small differences in usage by biosynthetic machinery or STs. Porcine ST3GAL1 (PDB 2WNB^64^) does not have an available co-crystal structure with CMP-Neu5Az but we have shown that human ST3GAL1 was able to transfer CMP-4AzNeu5Ac indicating the modification is tolerated by this ST. In human ST8SIA3 (PDB 5BO9^65^) and ST6GAL1 (PDB 6QVT^66^) co-crystal structures with CMP-3F_ax_-Neu5Ac and CMP-Neu5Ac respectively show the C4–OH positioned towards a pocket, suggesting the ability to accommodate C4 modifications. One previous study used 4Az3F_ax_Neu5Ac as a ST inhibitor and found that it worked nearly as well as the parental 3F_ax_Neu5Ac.^67^ For the other two positions (C7 and C9) the difference in their ability to be used as MOE agents, compared to Neu5Az, has been less rigorously studied. Given the efficient incorporation of 4AzNeu5Ac in cellular glycans, it is reasonable to use this as an alternative in cell-based sialylation studies when there may be a need to have the C5 derivatives with a different functional group. For example, one important application would be if it were desirable to include a photocrosslinker or biotin at C5, given that this location tolerates large groups.^43,68^

The emergence of Siglec-7 binding to sialylated core-1 mucin *O*-glycans in cell-based studies motivated us to quantify the strength of these interactions.^3,69–71^ We previously established that the *K*_d_ for Siglec-7 for (α-Neu5Ac-(2→3)-β-Gal*p*-(1→4)-α-Gal*p*NAc) Neu5Ac-LacNAc is 267 ± 26 μM as determined by COIN-Car-nMS.^54^ In this study, we determine that sialyl-T binds with considerably better affinity. This finding is already an important contribution in solidifying Siglec-7 prefers to bind mucin-type *O*-glycans.^72^ Moreover, we demonstrate that the 4AzNeu5Ac-derivatized sialyl-T structure 13 was recognized by Siglec-7 with an affinity not significantly different to that of 14. The similar affinities of these ligands combined with the previous observation that Siglec-7 can recognize an α1→4 linked sialic acid in bacteria LPS^23^ strongly suggests that Siglec-7 can accommodate functional groups appended to C4. These results can be rationalized by the published crystal structures of Siglec-7 (PDB: 2DF3,^73^2HRL^74^), which show that the C4–OH is solvent facing and is positioned away from the interaction interface, further supporting that the C4-azido group can be tolerated by Siglec-7. In the future, this observation can be leveraged to design higher affinity and selective ligands for engaging Siglec-7 for immunomodulation applications. Moreover, it will be of great interest to determine the affinities of other core-1 structures reported to bind Siglec-7, including disialyl- and trisialyl-T structures.^69,70^

## Conclusions

Taken together, our results expand the repertoire of biorthogonal sialic acid MOE agents with 4AzNeu5Ac and directly compares its incorporation efficiency into cellular glycans to the ‘industry standard’, Neu5Az. Our data supports that sialic acid with an azide appended to C4 can be efficiently metabolized by cellular machinery and provides an alternative tool when it may not be desirable to modify the C5 of sialic acid. Importantly, we describe a route to access the deprotected form of 4AzNeu5Ac, necessary in the chemoenzymatic synthesis of 4Azido-sialoglycans. Previously, 4-azido sialoglycans have not been synthesized, as assessing deprotected 4AzNeu5Ac has not been previously possible due to either reduction of the azide to an amine or, worse, degradation of the compound in a peeling reaction. We provide a route to access 4-azido-sialosides that can be leveraged in the future for diversification with alkynes.

## Author contributions

Author contributions were determined using CRediT for standardised contribution descriptions. T. E. G., K. B. L., G. C. D., and M. S. M. conceptualized the research goals and aims and participated in the writing of the manuscript. K. B. L, G. C. D, M. J., and D. K., synthesized the compounds, analyzed spectroscopy data, and participated in designing the synthetic routes. T. E. G. performed biological experiments including cell-based MOE, flow cytometry, and microscopy, including analysis of the results. D. T. B. performed and analyzed COIN-CaR-nMS studies. E. D. N. designed the plasmid and produced Siglec-7 from transfected CHO Flp-in cells. T. E. G. created the images and illustrations, and K. B. L. and T. E. G. created the chemical schemes for visualization and presentation. Funding was acquired by M. S. M. and T. E. G. M. S. M. and J. S. K. performed supervision.

## Data availability

Data for this manuscript are available in the main text (DOI) and ESI† (DOI). Raw data underlying the manuscript include flow cytometry data, microscopy images, compound characterization and mass spectrometry raw chromatograms are securely archived at the University of Alberta and are available upon request. Siglec-7 plasmids and Siglec-7 expressing CHO Flp-in cells used herein are available upon request. Synthetic compounds are available upon request as long as stocks last.

## Conflicts of interest

The authors declare that there are no conflicts.

## Supplementary Material

CB-OLF-D5CB00030K-s001
