# Supplementary material for: Synthesis of 4-azido sialic acid for testing against Siglec-7 and in metabolic oligosaccharide engineering
Source: RSC Chem Biol. 2025 Apr 17;6(6):869–81. doi: 10.1039/d5cb00030k (PMC12038855; doi:10.1039/d5cb00030k)
Supplement: CB-006-D5CB00030K-s001 [file CB-006-D5CB00030K-s001.pdf]

Supplementary Information (SI) for RSC Chemical Biology.
This journal is © The Royal Society of Chemistry 2025

Synthesis of 4-azido sialic acid for testing against Siglec-7 and in metabolic oligosaccharide engineering

Taylor E. Gray[1*], Kristin B. Labasan[1*], Gour Daskhan[1], Duong T. Bui[1], Maju Joe[1], Dhanraj Kumawat[1], Edward N. Schmidt[1], John S. Klassen[1], Matthew S. Macauley[1,2**]

[1]Department of Chemistry, University of Alberta, Edmonton, Canada, T6G 2G2;

[2] Department of Medical Microbiology and Immunology, University of Alberta, Edmonton, Canada T6G 2E1 **macauley@ualberta.ca

* Indicates authors contributed equally

Table of Contents

Experimental Procedures ***3***

Synthesis of Disaccharide acceptor **3**

Siglec-7 Fc production **9**

Siglec-7 Fc binding COIN-CaR-nMS **9**

Supplemental Schemes ***11***

Scheme S1: Synthetic scheme of β-Gal*p*-(1→3)-α-Gal*p*NAc acceptor 12 ..... **11**

Supplemental Figures ***12***

Figure S1: 4Az CMAS flow cytometry. **12**

1H and 13C NMR Spectra ***13***

Compound 2 ..... **13**

Compound 3 .... **14**
Compound 4 .... **15**
Compound 5 .... **16**
Compound 6 .... **17**
Compound 7 .... **18**
Compound 8 .... **20**
Compound 9 .... **21**
Compound 10 .... **22**
Compound 11 .... **23**
Compound S2 .... **24**
Compound S3 .... **26**
Compound S4 .... **28**
Compound S7 .... **30**
Compound S8 .... **32**
Compound S9 .... **34**
Compound 12 .... **36**
Compound 13 .... **38**
Compound 14 .... **40**
References ***41***

Experimental Procedures

Synthesis of Disaccharide Acceptor

2-(Benzyloxycarbonylamino)ethyl3,4,6-tri-*O*-acetyl2-azido-2-deoxy-α-D-galactopyranoside (S2).

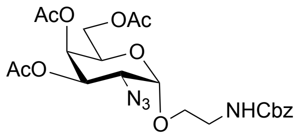

S2

A mixture of trichloroacetimidate **S1**[11]**(1)(1)**[11] (prepared from 371 mg, 1.12 mmol of the hemiacetal), *N*-Z-ethanolamine (262 mg, 1.34 mmol) and molecular sieves (4Å, 450 mg) in dry diethyl ether: dichloromethane (20:3, 23 mL) was stirred under argon for 30 min., cooled to -10 °C and TBSOTf (25 µL) was added dropwise and the stirring continued at -10 °C for about 10 min and then allowed to warm to -5 °C over the next 25 min before the acid was quenched by the addition of a few drops of triethylamine. The mixture was filtered through a pad of Celite and washed with CH_2Cl_2 (20 mL). The combined filtrate was concentrated, and the residue was purified by column chromatography (65:35, *n*-hexane–EtOAc) to afford the title compound **S2** as a thick syrup in 80% yield over two steps starting from the hemiacetal (α:β ratio 2.74:10. The α-product weighed 331 mg); R_f = 0.21 (65:35, *n*-hexane–EtOAc); Going forward only the α-product was used for further reactions.; ^{1}H NMR (700 MHz, $CDCl_3$) δ 7.34 – 7.26 (m, 5H), 5.40 (dd, *J* = 3.5, 1.4 Hz, 1H), 5.32 (dd, *J* = 11.2, 3.4 Hz, 2H), 5.08 (s, 2H), 4.97 (d, *J* = 3.6 Hz, 1H), 4.18 (td, *J* = 6.5, 1.4 Hz, 1H), 4.04 (d, *J* = 6.6 Hz, 2H), 3.81 – 3.76 (m, 1H), 3.65 – 3.58 (m, 2H), 3.50 – 3.43 (m, 1H), 3.40 – 3.35 (m, 1H), 2.11 (s, 3H), 2.02 (s, 3H), 1.99 (s, 3H).; ^{13}C NMR (176 MHz, $CDCl_3$) δ 170.43, 156.42, 136.45, 128.49, 128.09, 128.01, 98.40, 88.18, 68.26, 68.15, 67.51, 66.89, 66.74, 61.72, 57.53, 40.71, 20.60, 20.57, 20.55.; HRMS (ESI) calcd. for $(M+Na)^+$ $C_{22}H_{28}NaN_4O_{10}$ 508.1805, found 508.1698.

2-(Benzyloxycarbonylamino)ethyl 2-azido-2-deoxy-α-D-galactopyranoside (S3)

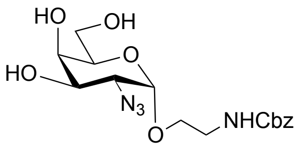

S3

To a solution of the compound **S2** in CH_2Cl_2–CH_3OH (7:2, 9 mL) was added catalytic sodium methoxide in CH_3OH to bring the pH of the reaction mixture to 8–9. After stirring for 24 h, the reaction mixture was neutralized by the addition of pre-washed Amberlite IR 120 H^+ resin. The solution was filtered and the filtrate was concentrated to a syrupy residue that was purified by column chromatography (9:1, DCM–CH_3OH) to give the title compound **S3** (201 mg, 81%) as a white foam; R_f = 0.20 (1:4, *n*-hexane–EtOAc); ^{1}H NMR (500 MHz, CD_3OD) δ 7.41 – 7.23 (m, 5H), 5.15 – 5.02 (m, 2H), 4.91 (d, *J* = 3.3 Hz, 1H), 3.97 (dd, *J* = 10.6, 2.8 Hz, 1H), 3.87 (d, *J* = 2.4 Hz, 1H), 3.83 (t, *J* = 5.9 Hz, 1H), 3.79 – 3.62 (m, 3H), 3.57 (dt, *J* = 10.6, 5.8 Hz, 1H), 3.44 (dd, *J* = 10.7, 3.6 Hz, 1H), 3.36 (t, *J* = 5.5 Hz, 2H).; ^{13}C NMR (176 MHz, $CDCl_3$ plus a few drops of CD_3OD) δ 160.91, 140.33, 132.36, 131.97, 131.79, 102.38, 74.36, 73.47, 72.06, 71.30, 70.62, 65.67, 64.21, 44.63.; HRMS (ESI) calcd. for $(M+Na)^+$ $C_{16}H_{22}NaN_4O_7$ 405.1381, found 405.1379.

2-(Benzyloxycarbonylamino)ethyl4,6-*O*-benzylidene-2-azido-2-deoxy-α-D-galactopyranoside (S4)

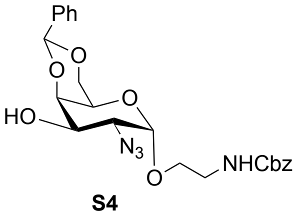

S4

To a solution of compound **S3** (200 mg, 0.520 mmol) in dry *N*, *N*-dimethylformamide (4 mL) was added benzaldehyde dimethylacetal (0.25 mL, 1.66 mmol) followed by camphorsulfonic acid in catalytic amounts (CSA, 12.0 mg). The reaction mixture was stirred overnight at 40-45 ºC under vacuum before DCM (3 mL), water (0.5 mL) and gl. acetic acid (0.5 mL) were added in succession.

After stirring for 30 min, the solution was diluted with DCM (40 mL), washed with water (3 X 15 mL), organic layer separated, dried (Na_2SO_4) and concentrated to a syrupy residue that was purified by column chromatography (1:1, *n*-hexane–EtOAc) to afford **S4** (222 mg, 90%) as a semi solid; R_f = 0.30 (1:1, *n*-hexane–EtOAc); ^{1}H NMR (700 MHz, $CDCl_3$) δ 7.50 – 7.46 (m, 2H), 7.41 – 7.29 (m, 8H), 5.55 (s, 1H), 5.22 (t, *J* = 6.0 Hz, 1H), 5.13 – 5.07 (m, 2H), 4.99 (d, *J* = 3.4 Hz, 1H), 4.27 – 4.21 (m, 2H), 4.14 (td, *J* = 10.6, 3.8 Hz, 1H), 4.01 (dd, *J* = 12.7, 1.8 Hz, 1H), 3.84 – 3.77 (m, 1H), 3.70 (s, 1H), 3.58 (dt, *J* = 10.7, 3.7 Hz, 2H), 3.52 – 3.44 (m, 1H), 3.42 – 3.36 (m, 1H), 2.49 (d, *J* = 10.8 Hz, 1H).; ^{13}C NMR (176 MHz, $CDCl_3$) δ 156.40, 137.23, 136.49, 129.39, 128.55, 128.34, 128.17, 128.10, 126.19, 101.27, 99.03, 75.36, 69.12, 67.92, 67.48, 66.77, 62.99, 60.81, 40.76.; HRMS (ESI) calcd. for $(M+Na)^+$ $C_{23}H_{26}NaN_4O_7$ 493.1694, found 493.1694.

2-(Benzyloxycarbonylamino)ethyl2-azido-3-*O*-[2,3,4,6-tetra-*O*-acetyl-β-D-galactopyranosyl]-4,6-*O*-benzylidene-2-deoxy-α-D-galactopyranoside (S6)

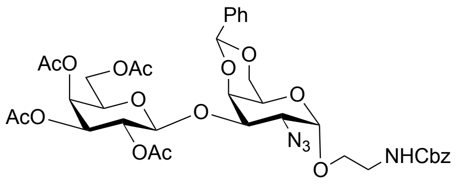

S6

Alcohol **S4** (220 mg, 0.460 mmol) and thioglycoside **S5**[2] (255 mg, 0.560 mmol) were dried under vacuum in the presence of P_2O_5 for 6 h prior to glycosylation. After drying, CH_2Cl_2 (13 mL) was added to it followed by powdered 4 Å molecular sieves (0.310 g) and stirred for 20 minutes. The reaction mixture was then cooled to 0°C and N-iodosuccinimide (152 mg, 0.670 mmol) and silver triflate (29.0 mg, 0.110 mmol) were added. After stirring the mixture for 30 min at 0 °C, the reaction was quenched by the addition of triethylamine until the pH of the solution was slightly basic. The reaction mixture was diluted with CH_2Cl_2 (20 mL) and filtered through Celite. The filtrate was washed with a saturated aq. solution of sodium thiosulphate (2 X 15 mL), water (15 mL) and brine (15 mL). The organic layer was separated, dried (Na_2SO_4), filtered and concentrated to a syrupy

residue which was purified by column chromatography (1:1, hexanes/EtOAc) to yield the title compound **S6** as a thick syrup (248 mg*, that was not completely pure and hence was used directly for the next step). R_f = 0.19 (1:1, *n*-hexane–EtOAc); HRMS (ESI) calcd. for $(M+Na)^+$ $C_{37}H_{44}NaN_4O_{16}$ 823.2645 found 823.2645.

***Note:** A major percentage of the required disaccharide was found to have the -STol group (from the donor compound **S5** used in glycosylation) attached to the nitrogen on the -NHCbz [-N(STol)Cbz; 127 mg, R_f = 0.61 (1:1, *n*-hexane–EtOAc)]. This was successfully converted back to the required compound **S9** in three steps (steps required for the conversion of compound **S7** to **S9**) thus improving the overall yield of the synthetic route.

2-(Benzyloxycarbonylamino)ethyl2-azido-3-*O*-[2,3,4,6-tetra-*O*-acetyl-β-D-galactopyranosyl]-2-deoxy-α-D-galactopyranoside (S7)

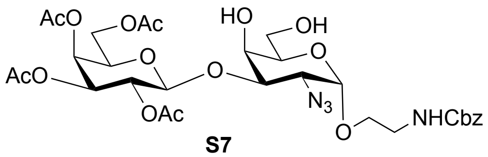

S7

To a solution of compound **S6** (248 mg impure sample from above) in $CH_3CN–H_2O$ (9:1, 10 mL) was added pyridinium *p*-toluenesulfonate (200 mg, 0.800 mmol) and heated at reflux for 22 h, cooled to room temperature and then concentrated to obtain a syrupy residue that was re-dissolved in dichloromethane (25 mL) and washed with water (2 X 10 mL). The organic layer was separated, dried (Na_2SO_4), filtered and concentrated to a syrupy residue which was purified by column chromatography (1:4, hexanes/EtOAc) to afford **S7** (100 mg, 30% over two steps*) as a thick syrup; R_f= 0.31 (1:4, *n*-hexane–EtOAc); ^1H NMR (700 MHz, CD_2Cl_2) δ 7.41 – 7.30 (m, 5H), 5.40 (dd, *J* = 3.5, 1.2 Hz, 1H), 5.35 – 5.32 (m, 2H), 5.24 (dd, *J* = 10.5, 8.0 Hz, 1H), 5.09 (s, 2H), 5.05 (dd, *J* = 10.5, 3.5 Hz, 1H), 4.98 (d, *J* = 3.6 Hz, 1H), 4.74 (d, *J* = 8.0 Hz, 1H), 4.20 – 4.16 (m, 2H), 4.13 – 4.07 (m, 2H), 4.03 (dd, *J* = 10.6, 3.2 Hz, 1H), 3.99 (ddd, *J* = 7.4, 5.3, 1.2 Hz, 1H), 3.88 – 3.84 (m, 2H), 3.82 – 3.77 (m, 1H), 3.77 – 3.72 (m, 1H), 3.66 – 3.58 (m, 2H), 3.48 – 3.38 (m,

2H), 2.86 (br. s, 1H), 2.16 (s, 3H), 2.07 (s, 3H), 2.03 (s, 3H), 1.98 (s, 3H).; ^{13}C NMR (176 MHz, CD_2Cl_2) δ 170.74, 170.49, 170.34, 169.83, 137.27, 128.85, 128.43, 128.30, 102.26, 99.14, 78.66, 71.78, 71.12, 70.25, 69.43, 68.75, 68.24, 67.45, 66.95, 62.92, 62.05, 59.06, 41.25, 20.83, 20.82, 20.80, 20.74.; HRMS (ESI) calcd. for $(M+Na)^+$ $C_{30}H_{40}NaN_4O_{16}$ 735.2332, found 735.2329.

*See the note under compound **S6**

2-(Benzyloxycarbonylamino)ethyl2-azido-3-*O*-[2,3,4,6-tetra-*O*-acetyl-β-D-galactopyranosyl]-4,6-di-*O*-acetyl-2-deoxy-α-D-galactopyranoside (S8)

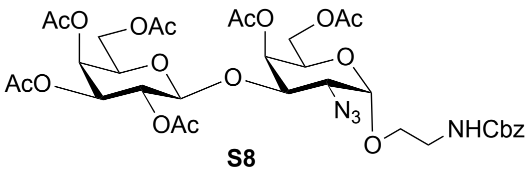

S8

To a solution of compound **S7** (176 mg, 0.250 mmol) in pyridine (5 mL) at < 5 ºC under nitrogen was added acetic anhydride (0.1 mL, 1.06 mmol) dropwise. The reaction mixture was allowed to come to r.t. and stirred for 24 h. The reaction mixture was then cooled to < 5 ºC followed by the addition of methanol (0.3 mL) dropwise, stirred for 30 minutes and concentrated to a syrupy residue which was purified by column chromatography (1:1, hexanes/EtOAc) to yield **S8** (176 mg, 90 %) as a semi solid. R_f = 0.29 (1:1, *n*-hexane–EtOAc); ^{1}H NMR (700 MHz, $CDCl_3$) δ 7.40 – 7.30 (m, 5H), 5.43 (d, *J* = 3.4 Hz, 1H), 5.34 (dd, *J* = 3.5, 1.2 Hz, 1H), 5.28 – 5.22 (m, 1H), 5.15 (dd, *J* = 10.5, 7.8 Hz, 1H), 5.10 (s, 2H), 4.98 (dd, *J* = 10.5, 3.4 Hz, 1H), 4.95 (d, *J* = 3.7 Hz, 1H), 4.67 (d, *J* = 7.9 Hz, 1H), 4.17 – 4.04 (m, 5H), 3.98 – 3.92 (m, 1H), 3.88 (ddd, *J* = 7.3, 6.2, 1.3 Hz, 1H), 3.79 – 3.73 (m, 1H), 3.66 – 3.60 (m, 2H), 3.50 – 3.44 (m, 1H), 3.44 – 3.37 (m, 1H), 2.13 (s, 3H), 2.10 (s, 3H), 2.04 (s, 3H), 2.02 (s, 3H), 2.01 (s, 3H), 1.96 (s, 3H).; ^{13}C NMR (176 MHz, $CDCl_3$) δ 170.48, 170.36, 170.22, 170.06, 169.62, 169.46, 156.34, 136.37, 128.55, 128.22, 128.12, 101.49, 98.34, 74.62, 70.82, 70.78, 69.33, 68.76, 68.24, 67.72, 66.84, 66.76, 62.76, 60.98, 59.61, 40.74,

20.70, 20.68, 20.66, 20.64, 20.61, 20.52.; HRMS (ESI) calcd. for $(M+Na)^+$ $C_{34}H_{44}NaN_4O_{18}$ 819.2543, found 819.2541.

2-(Benzyloxycarbonylamino)ethyl2-acetamido-3-*O*-[2,3,4,6-tetra-*O*-acetyl-β-D-galactopyranosyl]-4,6-di-*O*-acetyl-2-deoxy-α-D-galactopyranoside (S9)

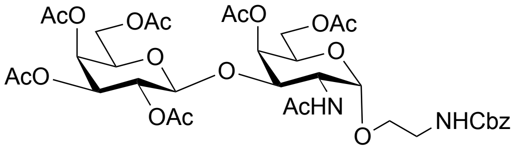

S9

To a solution of azide **S8** (176 mg, 0.220 mmol) in dry pyridine (6 mL) was added CH_3COSH (2.1 mL), and the solution was stirred at r.t. under nitrogen for 7 days. The reaction mixture was concentrated to a syrupy residue which was purified by column chromatography (9:1, DCM/CH_3OH) to yield **S9** (180 mg, quantitative) as a semi solid. R_f = 0.22 (neat EtOAc); ^{1}H NMR (700 MHz, $CDCl_3$) δ 7.38 – 7.28 (m, 5H), 6.03 (d, *J* = 8.8 Hz, 1H), 5.35 – 5.30 (m, 2H), 5.20 – 5.05 (m, 4H), 5.13 – 5.04 (m, 3H), 4.96 – 4.90 (m, 2H), 4.54 (d, *J* = 7.9 Hz, 1H), 4.48 (ddd, *J* = 10.9, 8.7, 3.6 Hz, 1H), 4.15 – 4.05 (m, 4H), 3.97 (dd, *J* = 10.8, 6.7 Hz, 1H), 3.88 – 3.81 (m, 2H), 3.71 (ddd, *J* = 10.9, 7.3, 3.6 Hz, 1H), 3.58 (ddd, *J* = 10.4, 6.1, 3.5 Hz, 1H), 3.47 – 3.41 (m, 1H), 3.39 – 3.32 (m, 1H), 2.13 (s, 3H), 2.11 (s, 3H), 2.06 (s, 3H), 2.02 (s, 3H), 2.01 (s, 3H), 1.97 (s, 3H), 1.95 (s, 3H); ^{13}C NMR (176 MHz, $CDCl_3$) δ 170.57, 170.39, 170.29, 170.13, 169.99, 169.70, 156.70, 136.23, 128.62, 128.55, 128.33, 128.01, 100.64, 98.40, 73.07, 70.83, 70.73, 68.68, 68.58, 68.55, 67.57, 66.86, 66.71, 62.81, 61.00, 48.83, 41.01, 23.23, 20.72, 20.71, 20.70, 20.69, 20.64, 20.52.; HRMS (ESI) calcd. for $(M+Na)^+$ $C_{36}H_{48}NaN_2O_{19}$ 835.2743, found 835.2750.

2-(Benzyloxycarbonylamino)ethyl-2-acetamido-3-*O*-[β-D-galactopyranosyl]-2-deoxy-α-D-galactopyranoside (12)

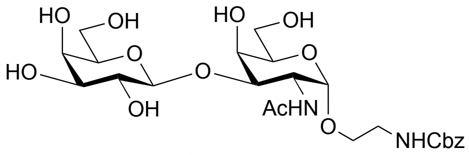

12

To a solution of compound **S9** (102 mg, 0.130 mmol) in CH_2Cl_2–CH_3OH (3:1, 8 mL) was added sodium methoxide in CH_3OH to bring the pH of the reaction mixture to 8–9. The solution was stirred for 48h and was then neutralized by the addition of pre-washed Amberlite IR 120 H^+ resin, filtered and the filtrate was concentrated to a syrupy residue that was purified by a C-18 column (water-methanol, gradient elution) to afford the title compound **12** (63.0 mg, 90 %) as a white fluffy material. R_f = 0.40 (36:9:9:6, EtOAc: CH_3OH: AcOH: water); ^{1}H NMR (700 MHz, D_2O) δ 7.50 – 7.37 (m, 5H), 5.17 (d, J = 12.3 Hz, 1H), 5.11 (d, J = 12.6 Hz, 1H), 4.86 (d, J = 3.8 Hz, 1H), 4.43 (d, J = 7.8 Hz, 1H), 4.33 (dd, J = 11.0, 3.8 Hz, 1H), 4.19 (d, J = 3.1 Hz, 1H), 3.97 (dd, J = 11.0, 3.1 Hz, 1H), 3.94 – 3.90 (m, 2H), 3.78 – 3.71 (m, 4H), 3.69 (dd, J = 11.7, 4.4 Hz, 1H), 3.66 – 3.59 (m, 2H), 3.57 – 3.50 (m, 2H), 3.44 (ddd, J = 14.6, 7.1, 3.7 Hz, 1H), 3.35 – 3.30 (m, 1H), 1.98 (s, 3H).; ^{13}C NMR (176 MHz, D_2O) δ 175.49, 159.48, 137.45, 129.81, 129.42, 128.63, 105.72, 98.31, 78.26, 75.92, 73.50, 71.68, 71.58, 69.70, 69.53, 67.85, 67.78, 62.12, 61.88, 49.51, 41.22, 22.98.; HRMS (ESI) calcd. for $(M+Na)^+$ $C_{24}H_{36}NaN_2O_{13}$ 583.2110, found 583.2107.

Siglec-7 Fc production

Siglec-7 Fc was cloned, stably transfected in CHO Flp-In cells, expressed, and purified exactly as previously described with no modifications.[3, 4]

Siglec-7 Fc binding COIN-CaR-nMS

Protein-ligand affinity measurements were performed as recently described.[5-7] In short, all measurements were performed in negative ion mode using a Q Exactive Ultra-High Mass Range (UHMR) Orbitrap mass spectrometer (Thermo Fisher Scientific, Bremen, Germany) with a nano-ESI source. The nano-ESI emitter was loaded with 2 solutions –solution 1 contained Siglec-7 Fc

and ligands **13** and **14** (0.5 μM), solution 2 contained Siglec-7 (at an identical concentration as in solution 1) and ligands **13** and **14** of interest (30 – 40 μM). To perform nanoESI, a voltage of approximately -0.7 kV was applied to a platinum wire. The solution temperature was 25 °C. Resolution of 25000 was used. Maximum injection time was 200 ms, the S-lens RF level was 200 and DC offset was 21. Collision energy was 120 V. Raw data were processed using the Thermo Xcalibur 4.4 software. Time-resolved mass spectra were averaged over 1 min intervals and the sum of the charge state-normalized abundances of the reactant and the complex ions were calculated automatically using the SWARM software.[2] The K_d values were obtained by fitting with Igor pro (WaveMetrics Inc., Lake Oswego, OR, USA) using Eq1:

$$F_t = DE\frac{[\mathrm{P}]_0 + (0.5 + C_L t) + K_\mathrm{d} - \sqrt{(K_\mathrm{d} - (0.5 + C_L t) + [\mathrm{P}]_0)^2 + 4K_\mathrm{d}(0.5 + \mathrm{C}_L t)}}{2[\mathrm{P}]_0} \quad \text{(Eq1)}$$

where *DE* is the detection efficiency of the released glycan relative to the GBP, $C_L(t)$ is the *t*-dependent function that describes the change in ligand concentration due to diffusion and advection, $[P]_0$ is initial protein concentration, the time-dependent fractional binding site occupancy (fraction bound, F_t) of P, was calculated using the time-dependent abundance (Ab_t) of the released ligand and free protein as shown in eq Eq2,

$$F_t = \frac{Ab_t(\mathrm{L})}{Ab_t(\mathrm{P})} \quad \text{(Eq2)}$$

Supplemental Schemes

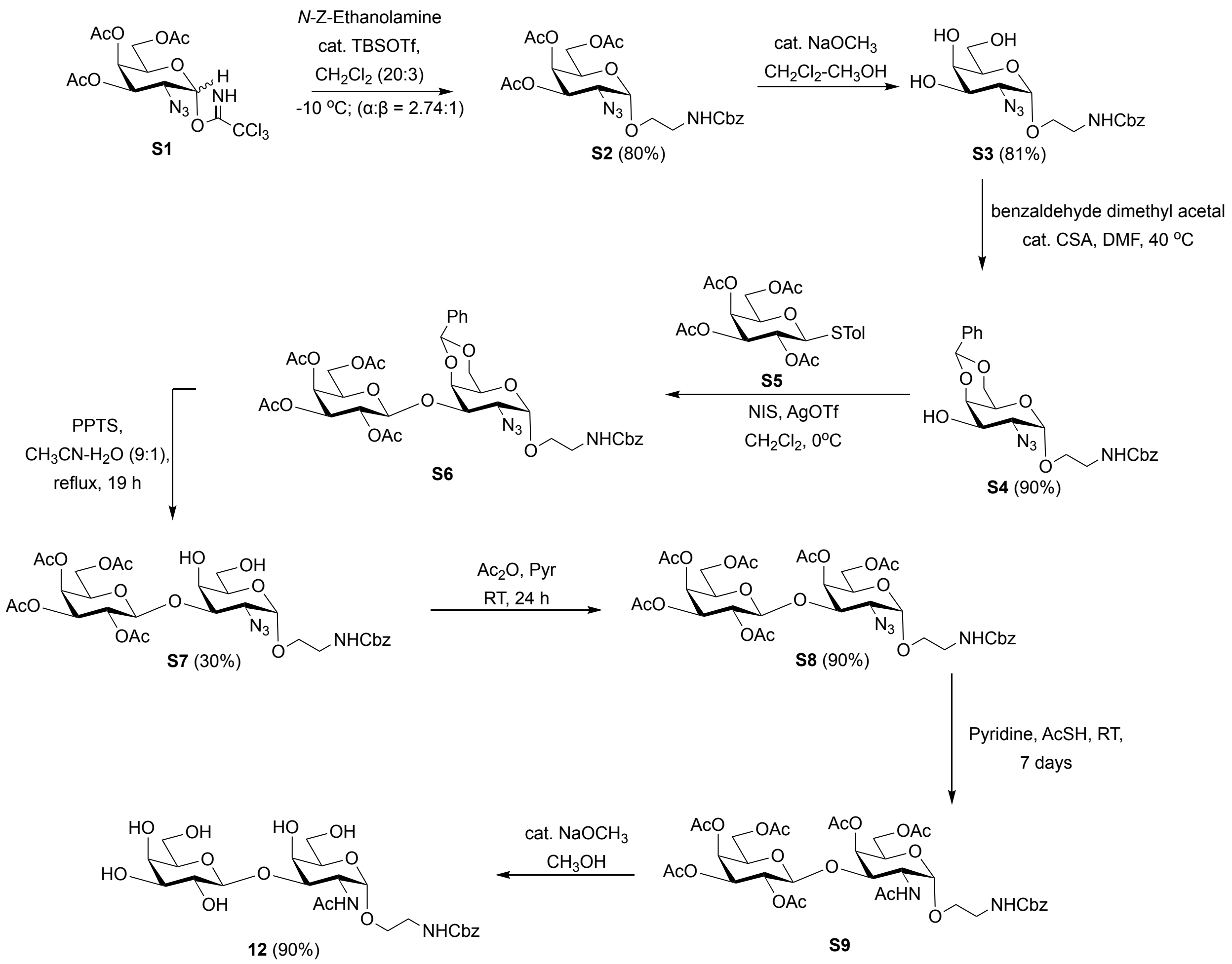

*Scheme S1: Synthetic scheme of β-Galp-(1→3)-α-GalpNAc acceptor **12***

Supplemental Figures

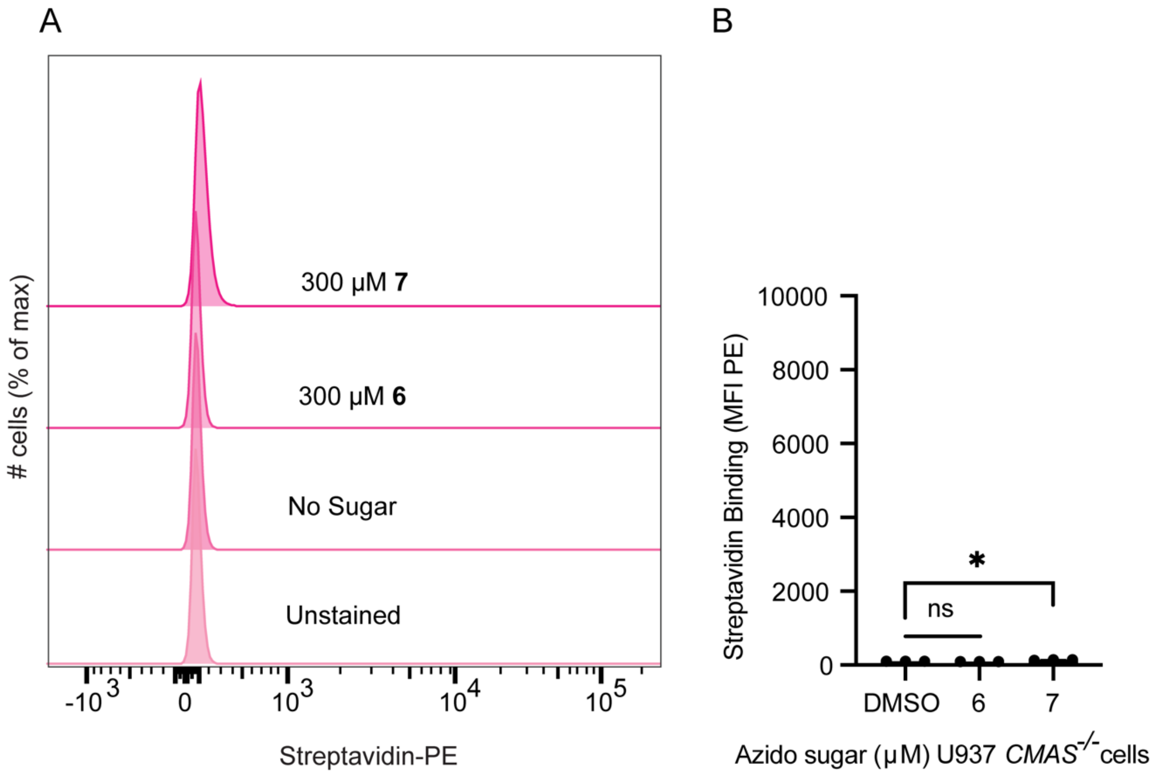

Figure S1: 4Az CMAS flow cytometry.

(A) Representative histograms showing U937 $CMAS^{-/-}$ cells fed **6** and **7** and (B) quantification. Two-tailed Student's paired t-test was used for statistical analysis. Not Significant (ns), P > 0.05, *,P=0.0197.

^{1}H and ^{13}C NMR Spectra

*Compound **2***

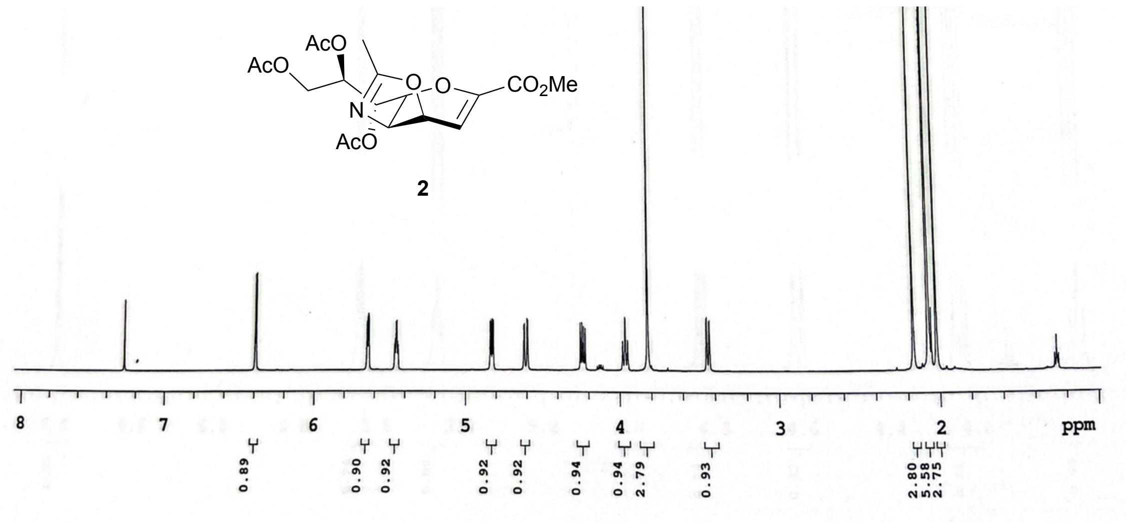

^{1}H NMR spectrum ($CDCl_3$, 600 MHz).

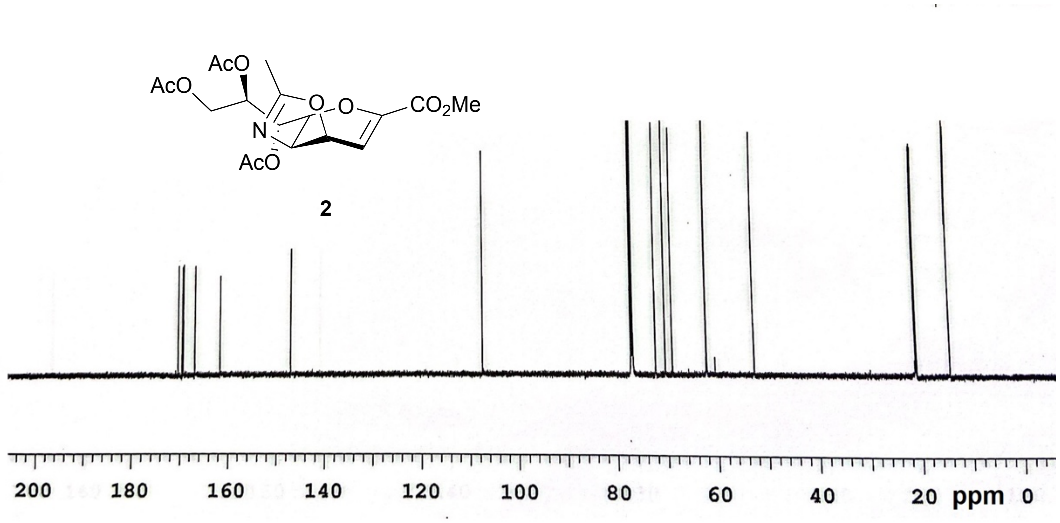

^{13}C NMR spectrum ($CDCl_3$, 176 MHz).

*Compound **3***

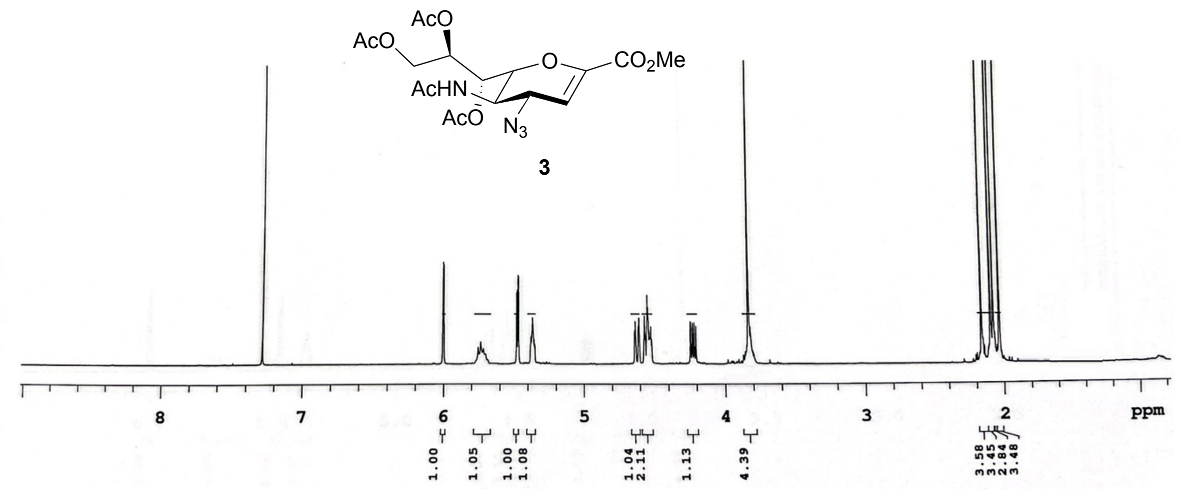

^{1}H NMR spectrum ($CDCl_3$, 500 MHz).

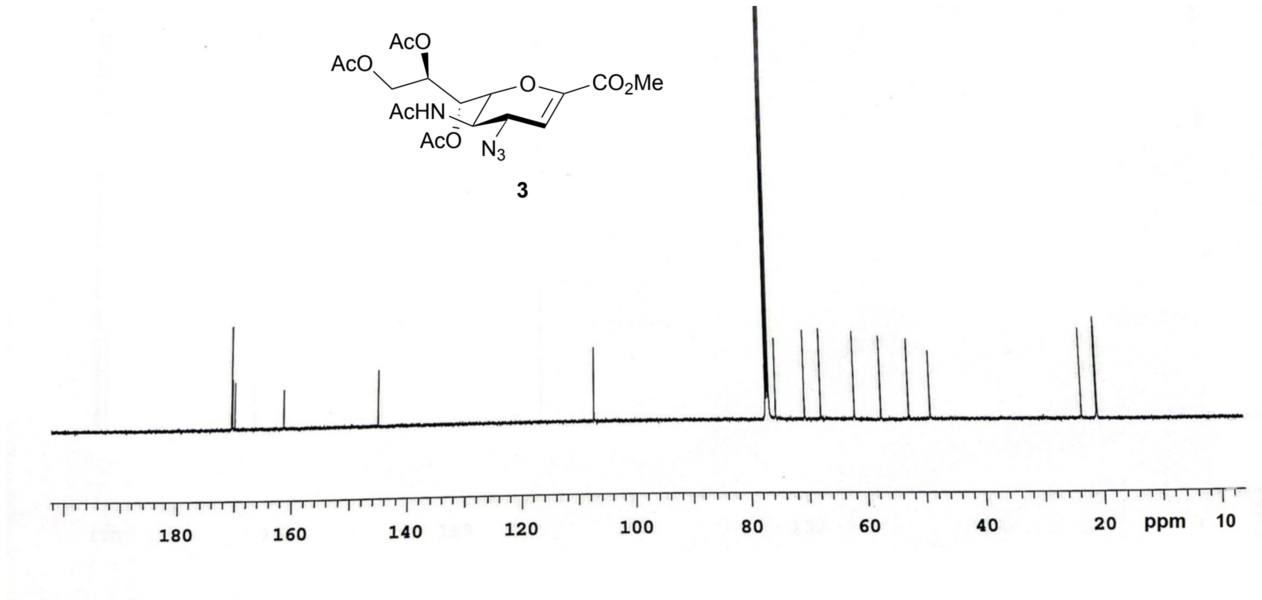

^{13}C NMR spectrum ($CDCl_3$, 176 MHz).

*Compound **4***

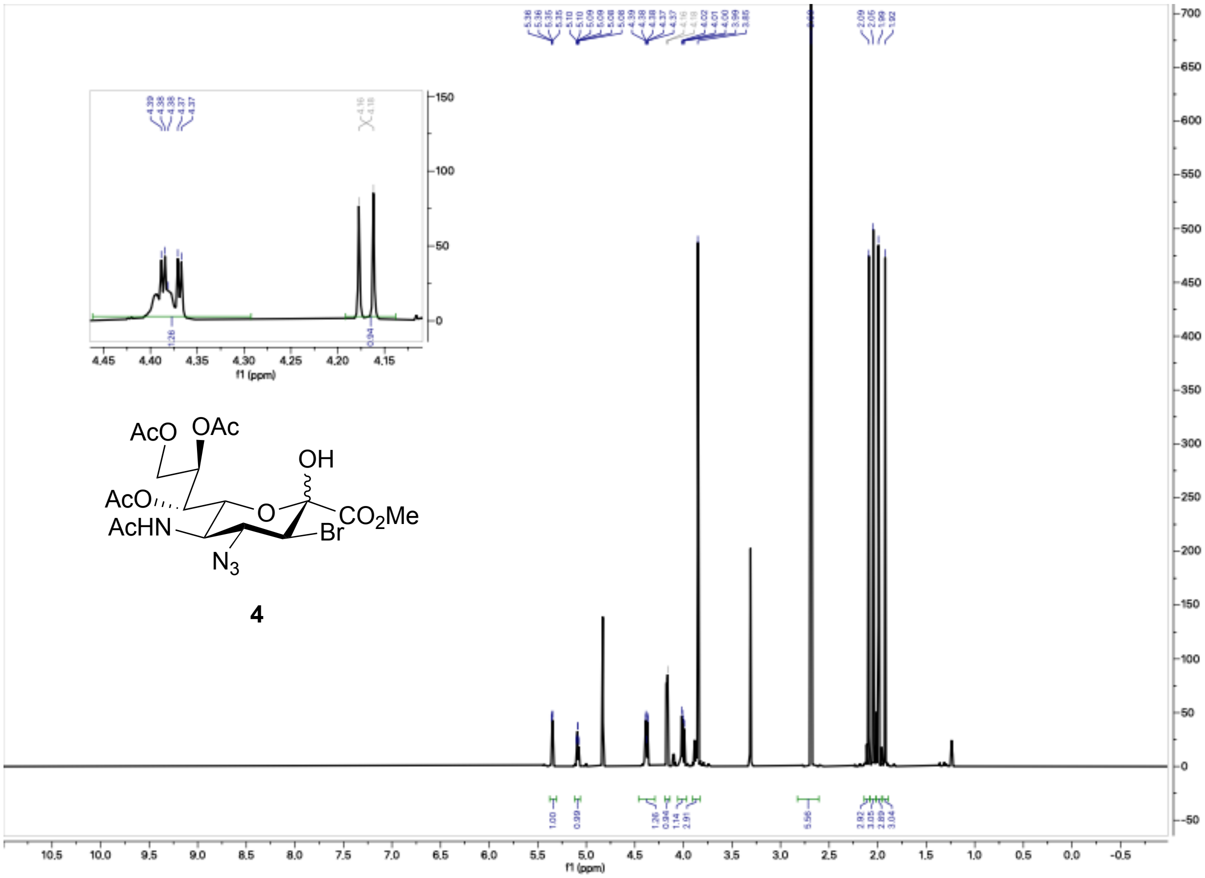

^{1}H NMR spectrum (CD_3OD, 700 MHz).

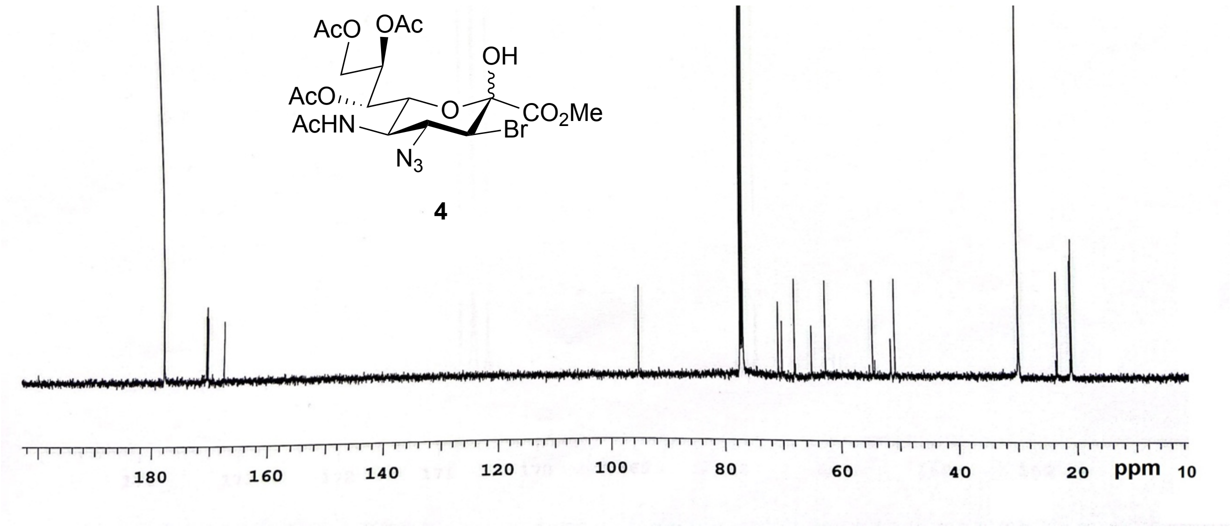

^{13}C NMR spectrum ($CDCl_3$, 125 MHz).

*Compound **5***

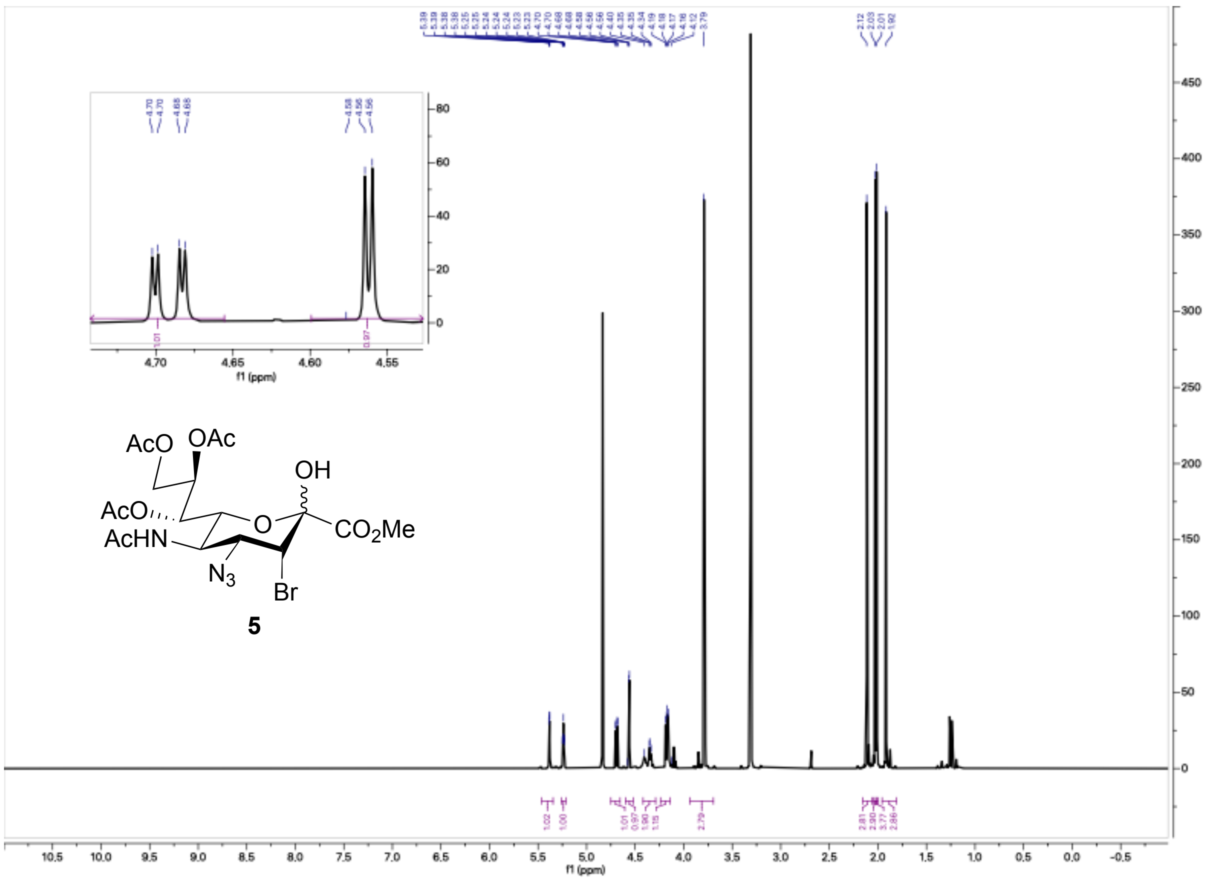

^1H NMR spectrum (CD_3OD, 700 MHz).

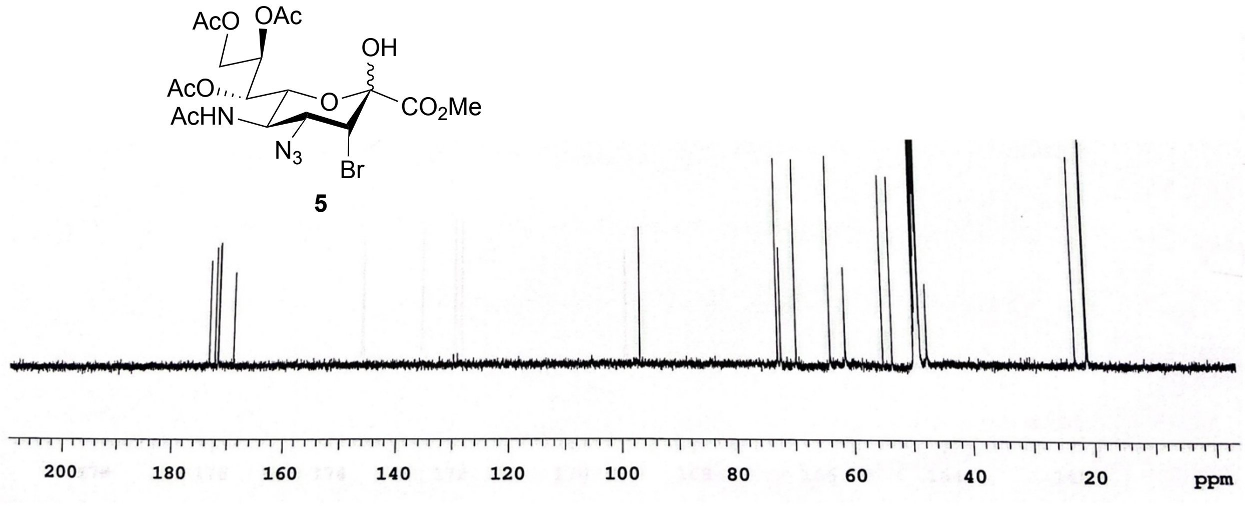

^{13}C NMR spectrum (CD_3OD, 125 MHz).

*Compound **6***

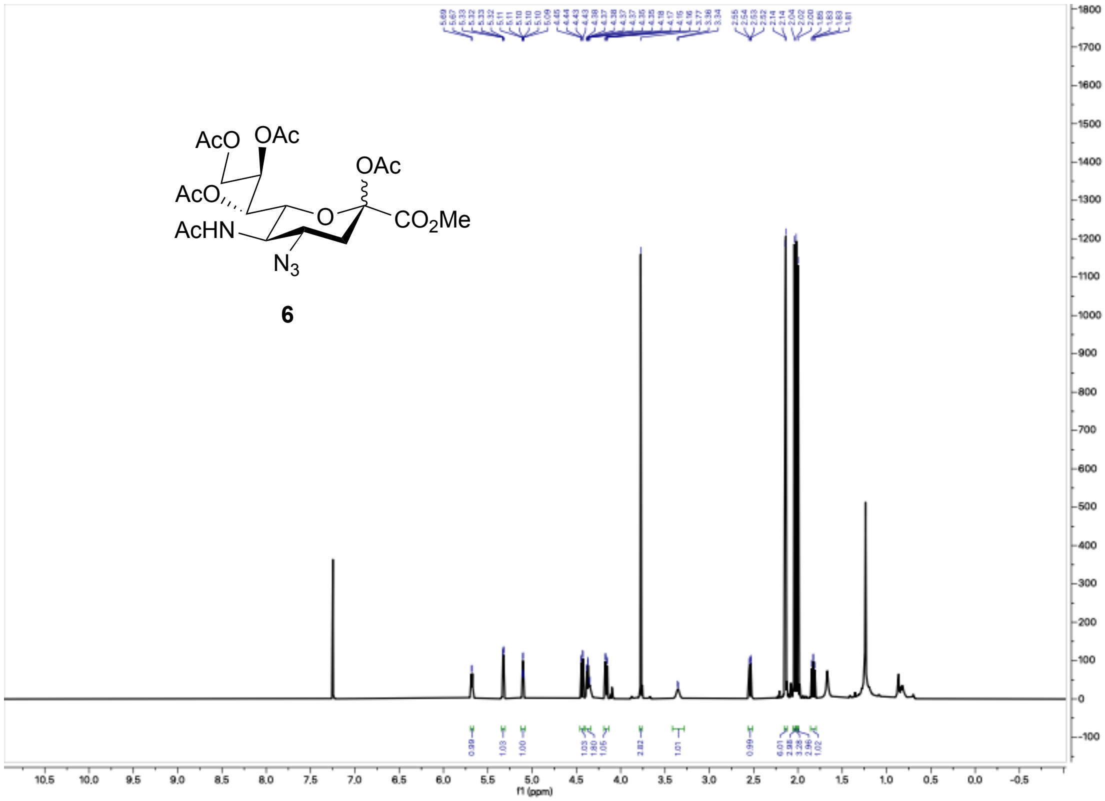

^{1}H NMR spectrum ($CDCl_3$, 700 MHz).

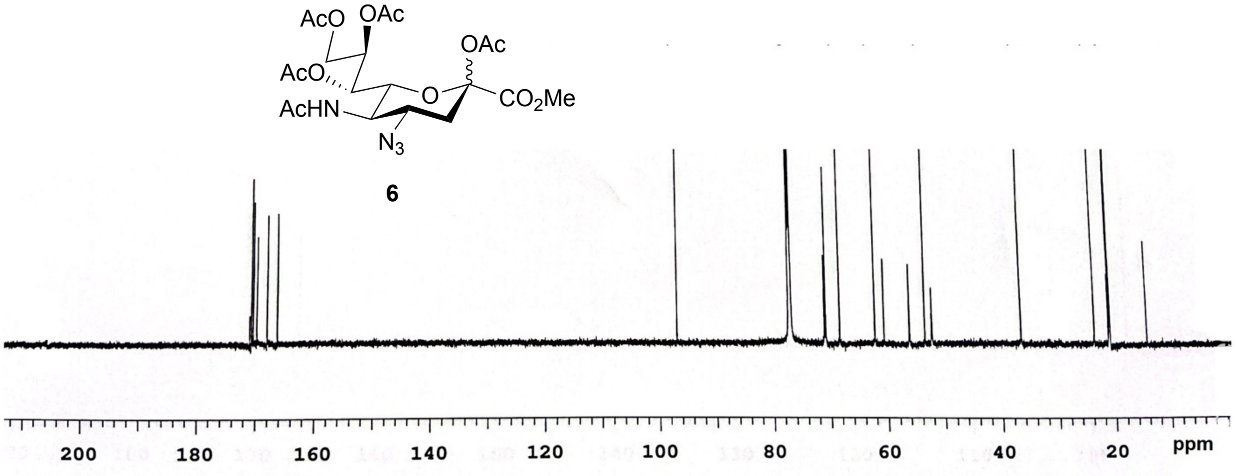

^{13}C NMR spectrum ($CDCl_3$, 176 MHz).

Compound ***7***

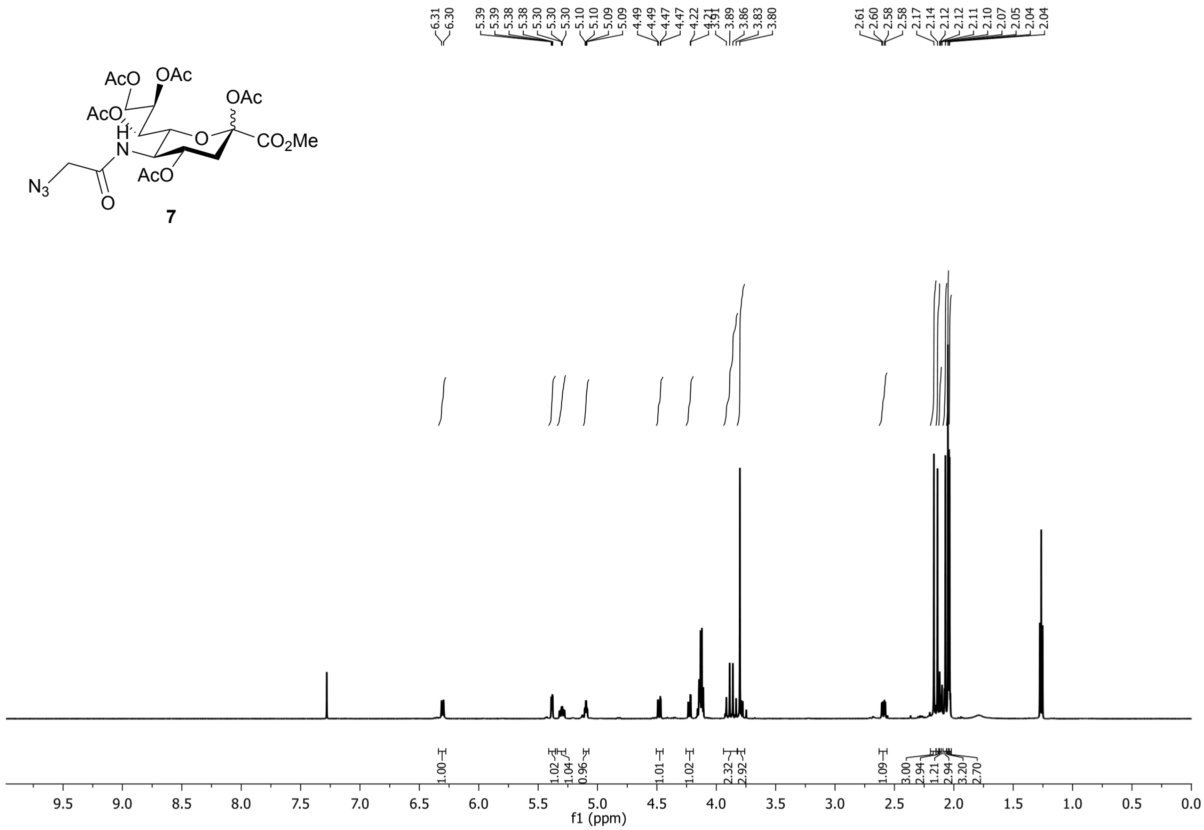

^{1}H NMR spectrum of peracetylated Neu5Az **7** ($CDCl_3$, 600 MHz).

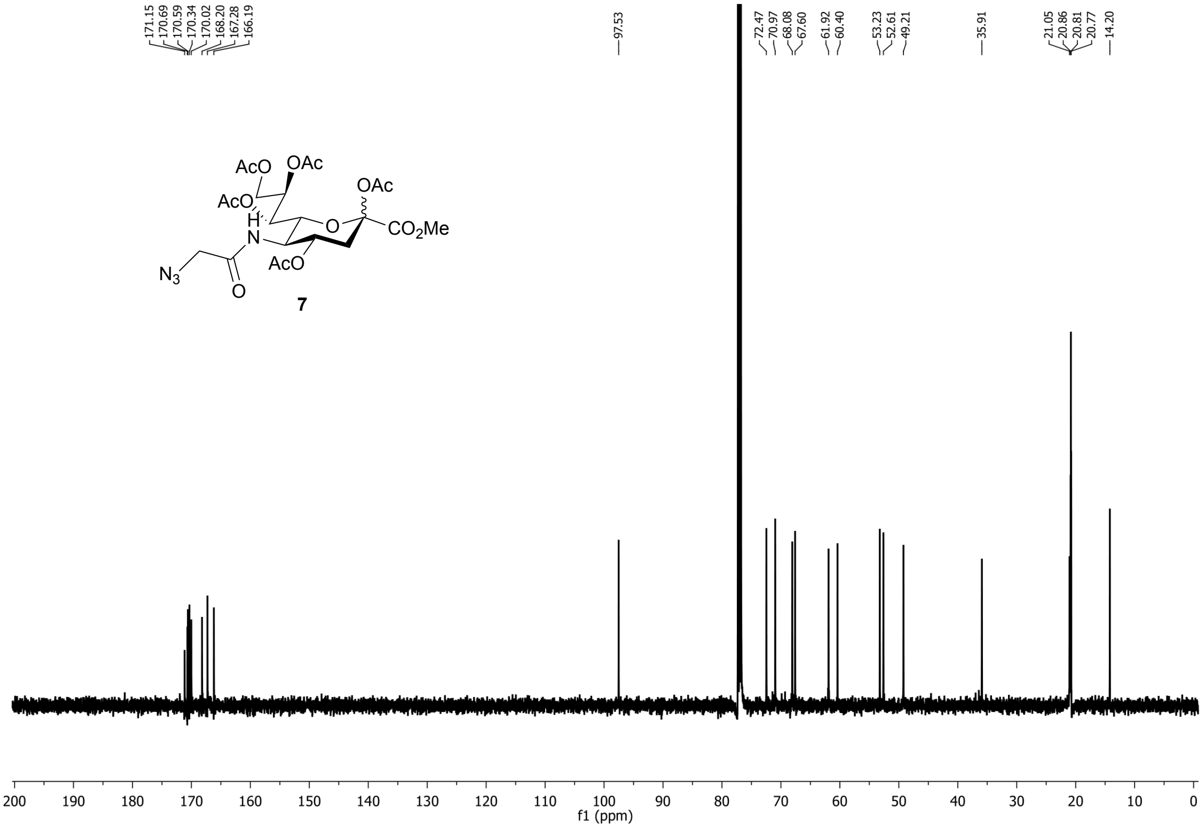

^{13}C NMR spectrum of peracetylated Neu5Az **7** ($CDCl_3$, 125 MHz).

*Compound **8***

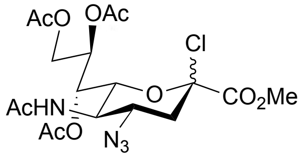

8

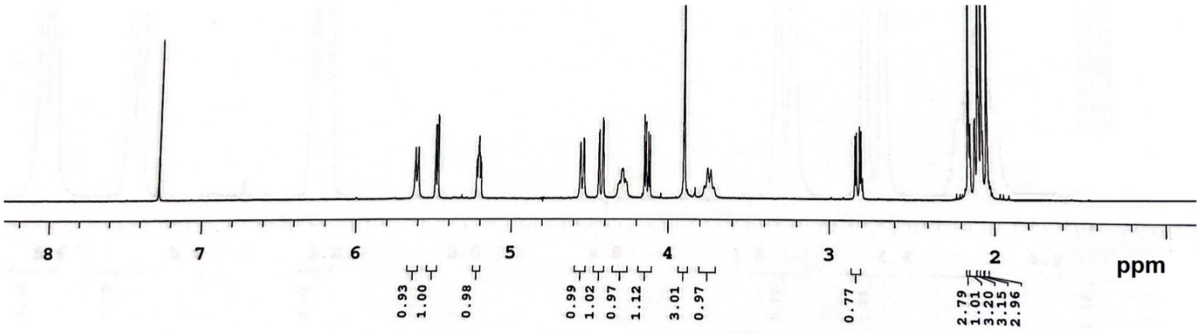

^{1}H NMR spectrum ($CDCl_3$, 500 MHz).

*Compound **9***

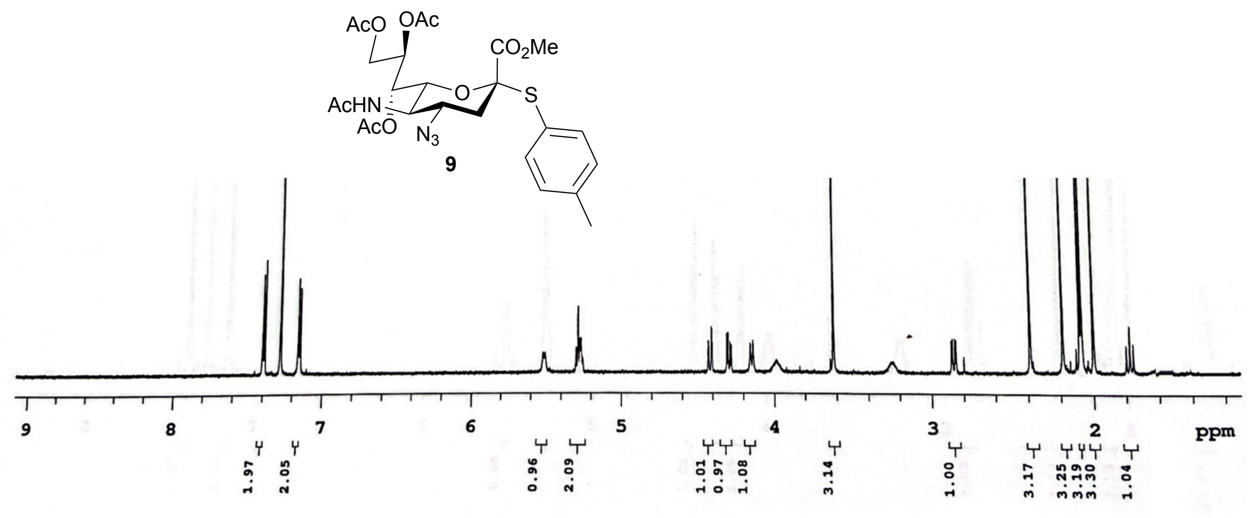

^{1}H NMR spectrum ($CDCl_3$, 600 MHz).

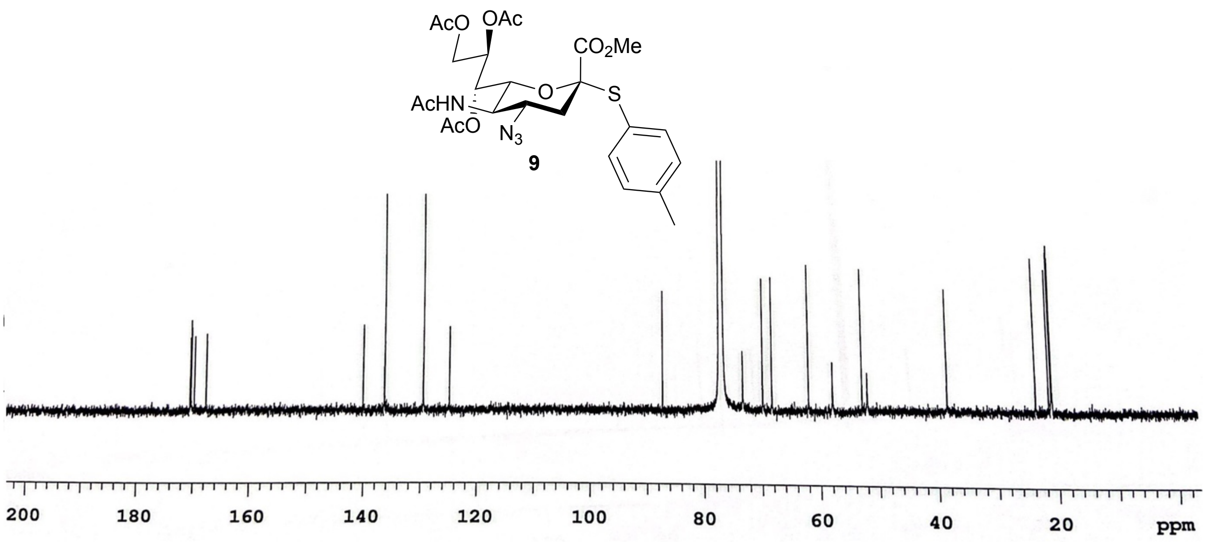

^{13}C NMR spectrum ($CDCl_3$, 125 MHz).

*Compound **10***

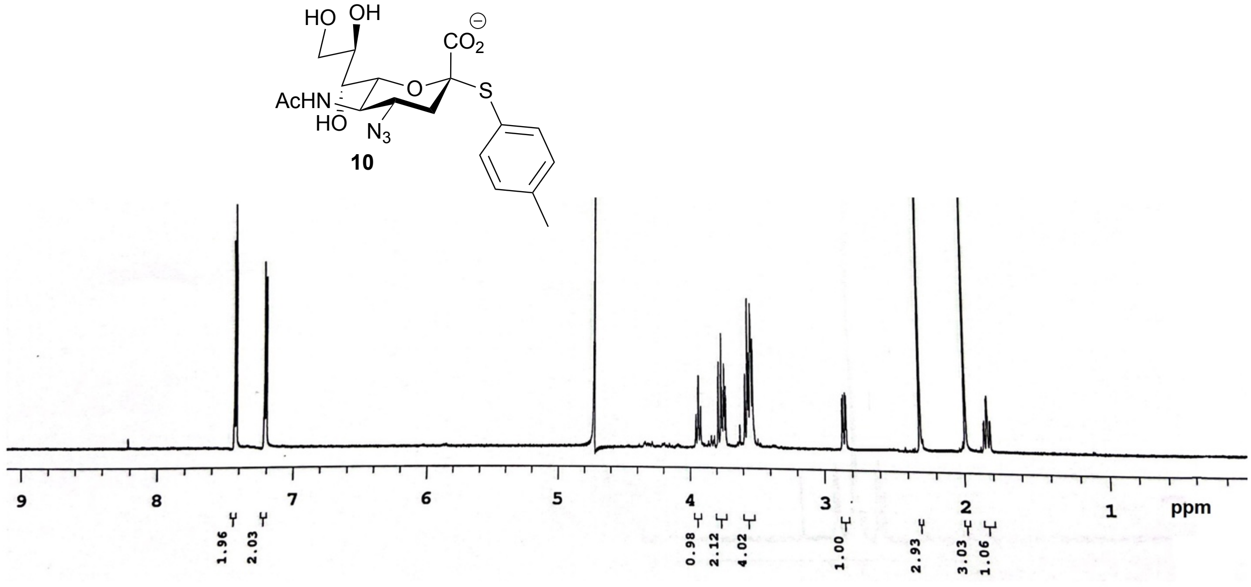

^{1}H NMR spectrum (D_2O, 600 MHz).

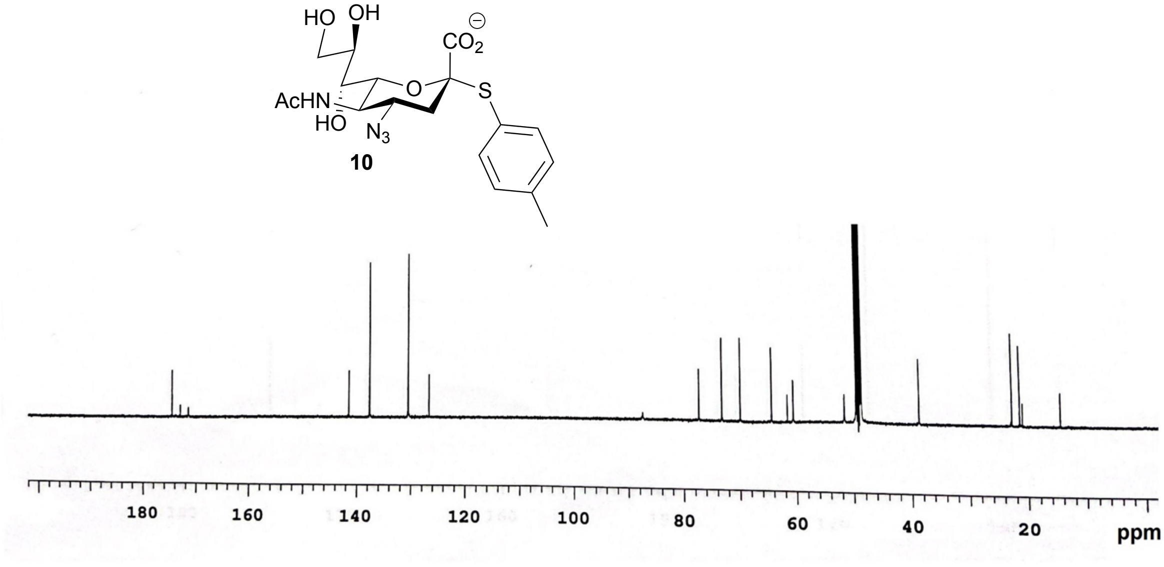

^{13}C NMR spectrum (D_2O, 176 MHz).

*Compound **11***

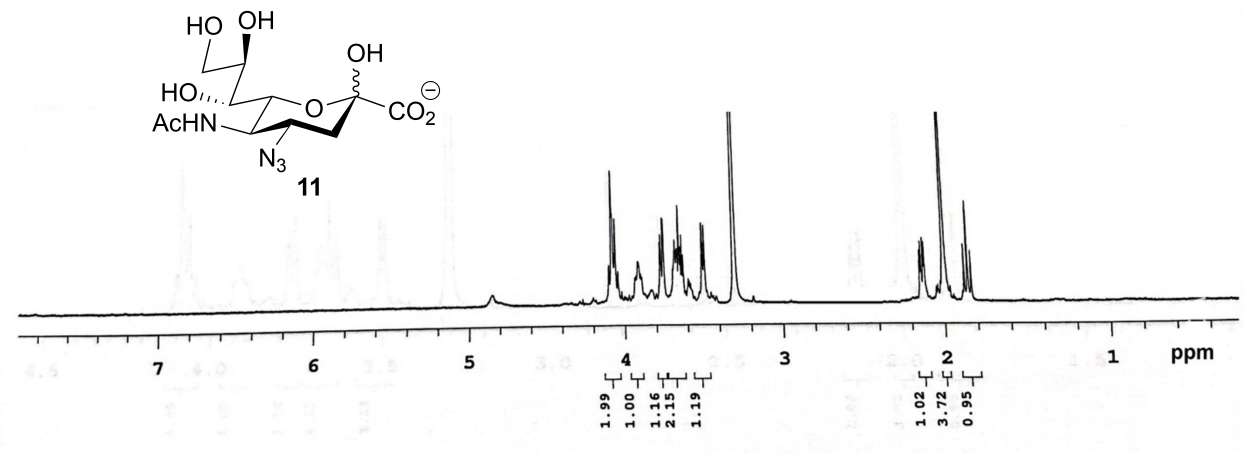

^{1}H NMR spectrum (CD_3OD, 500 MHz).

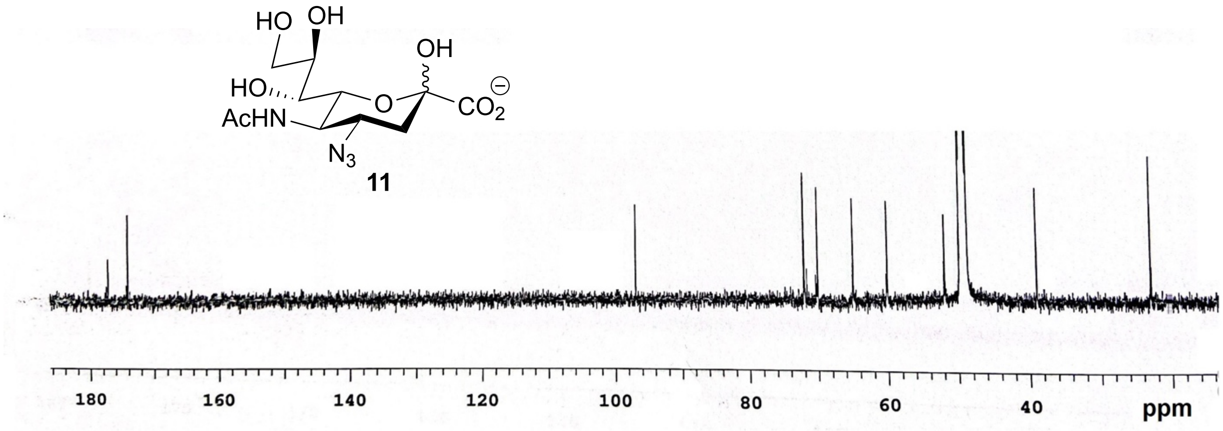

^{13}C NMR spectrum (CD_3OD, 125 MHz).

Compound ***S2***

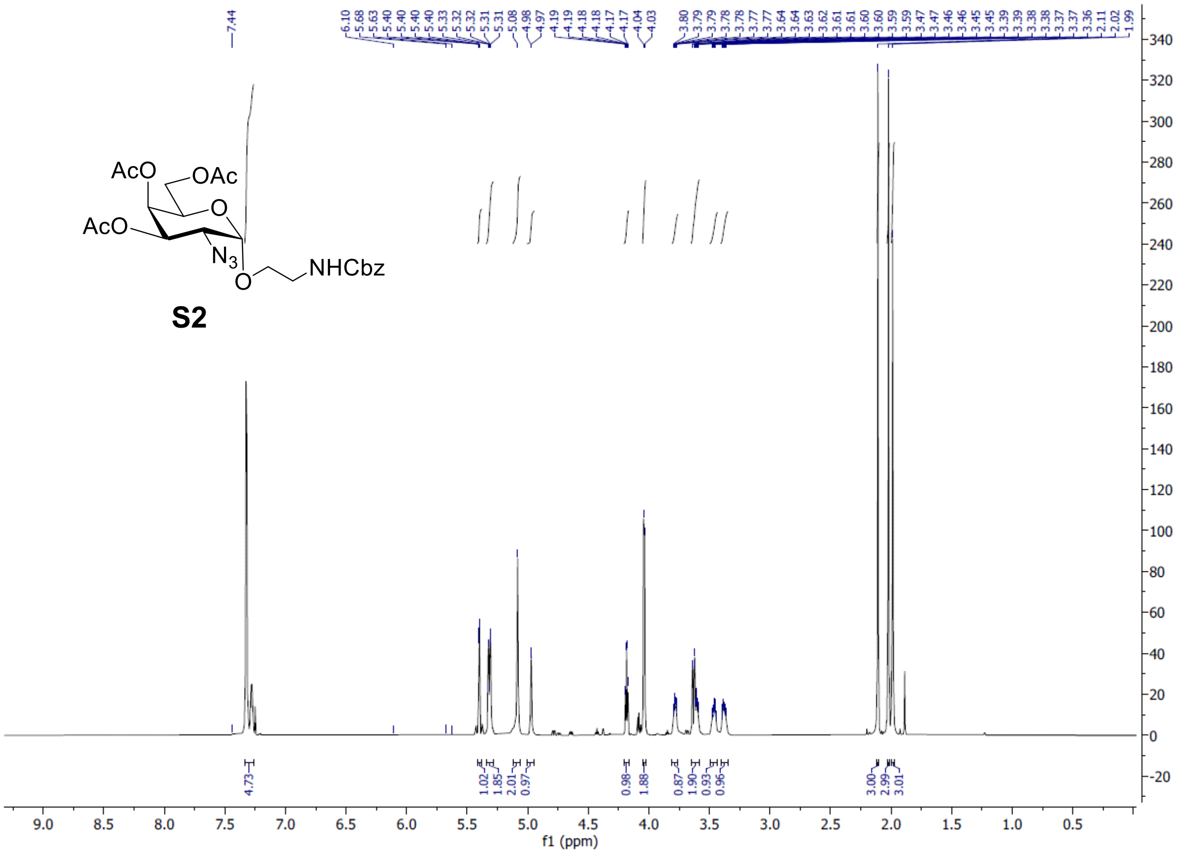

^{1}H NMR spectrum ($CDCl_3$, 700 MHz).

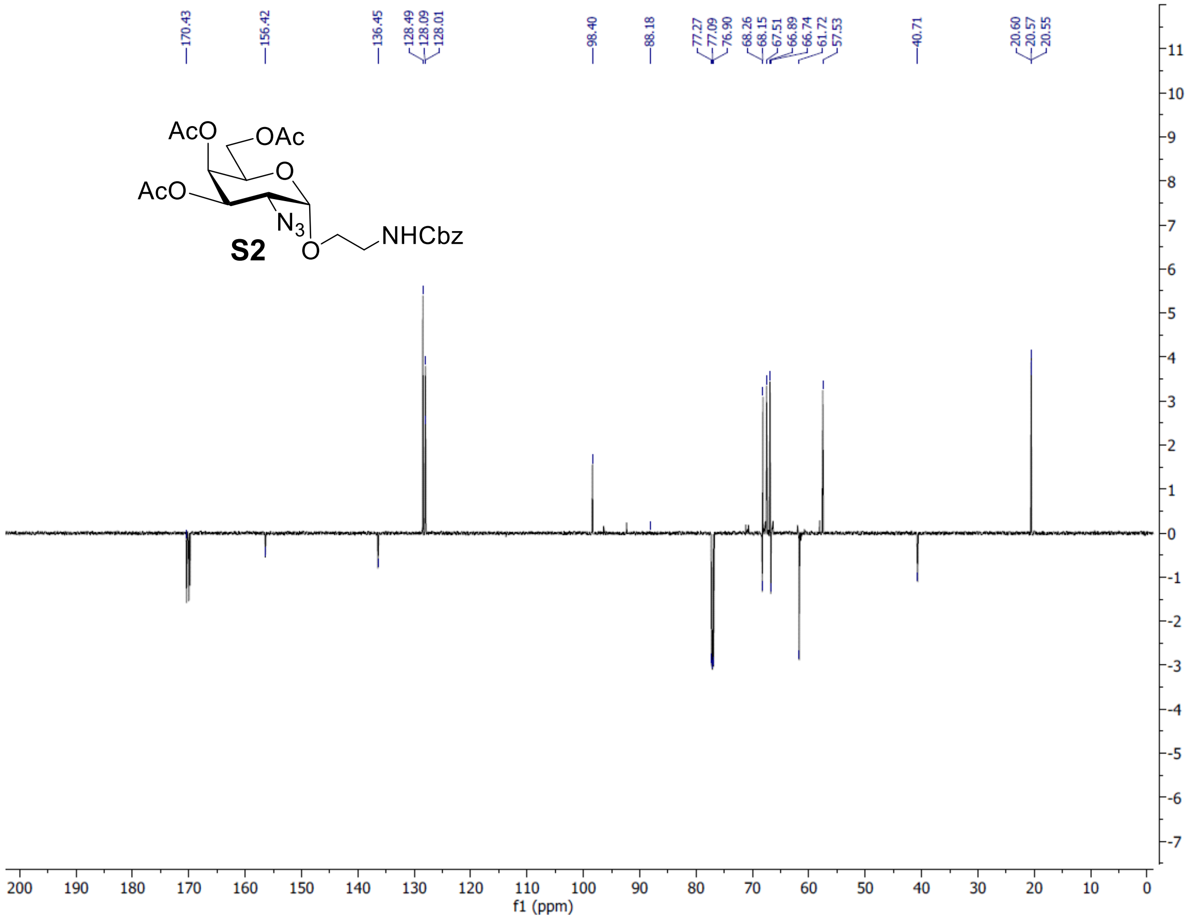

^{13}C NMR spectrum ($CDCl_3$, 176 MHz).

Compound ***S3***

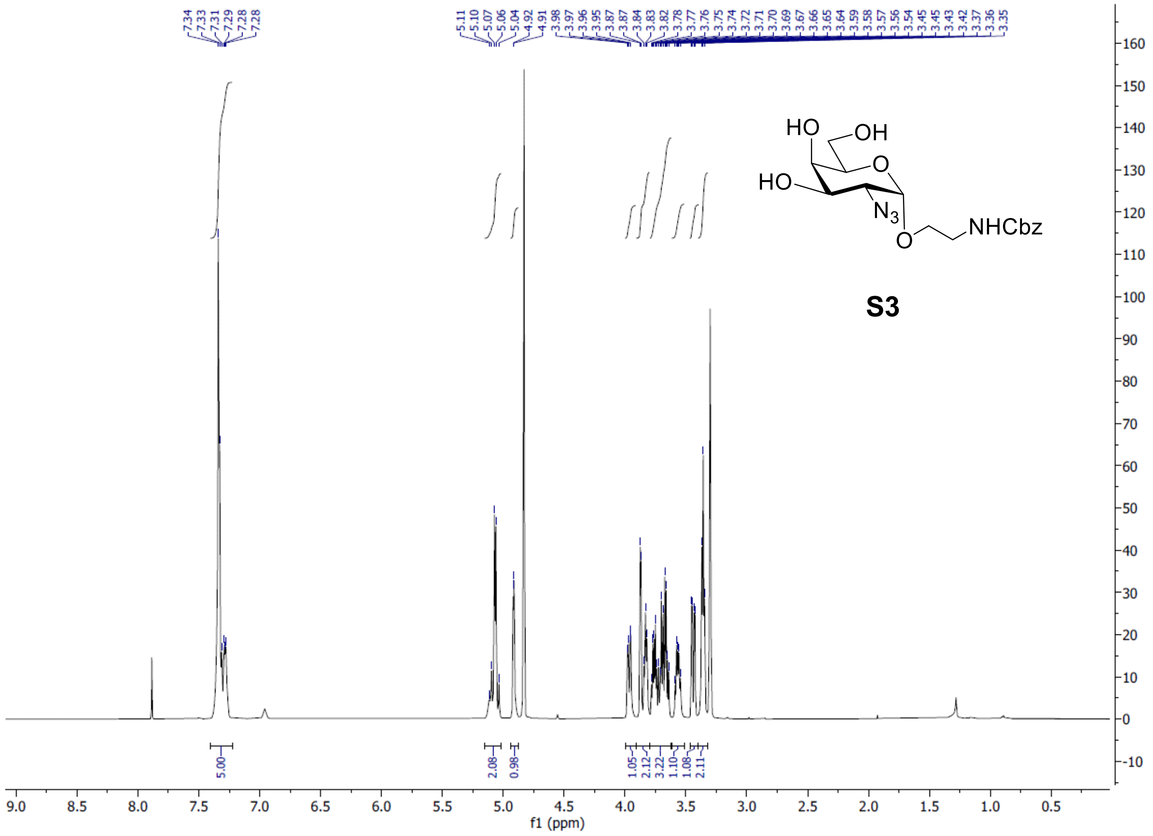

^{1}H NMR spectrum (CD_3OD, 500 MHz).

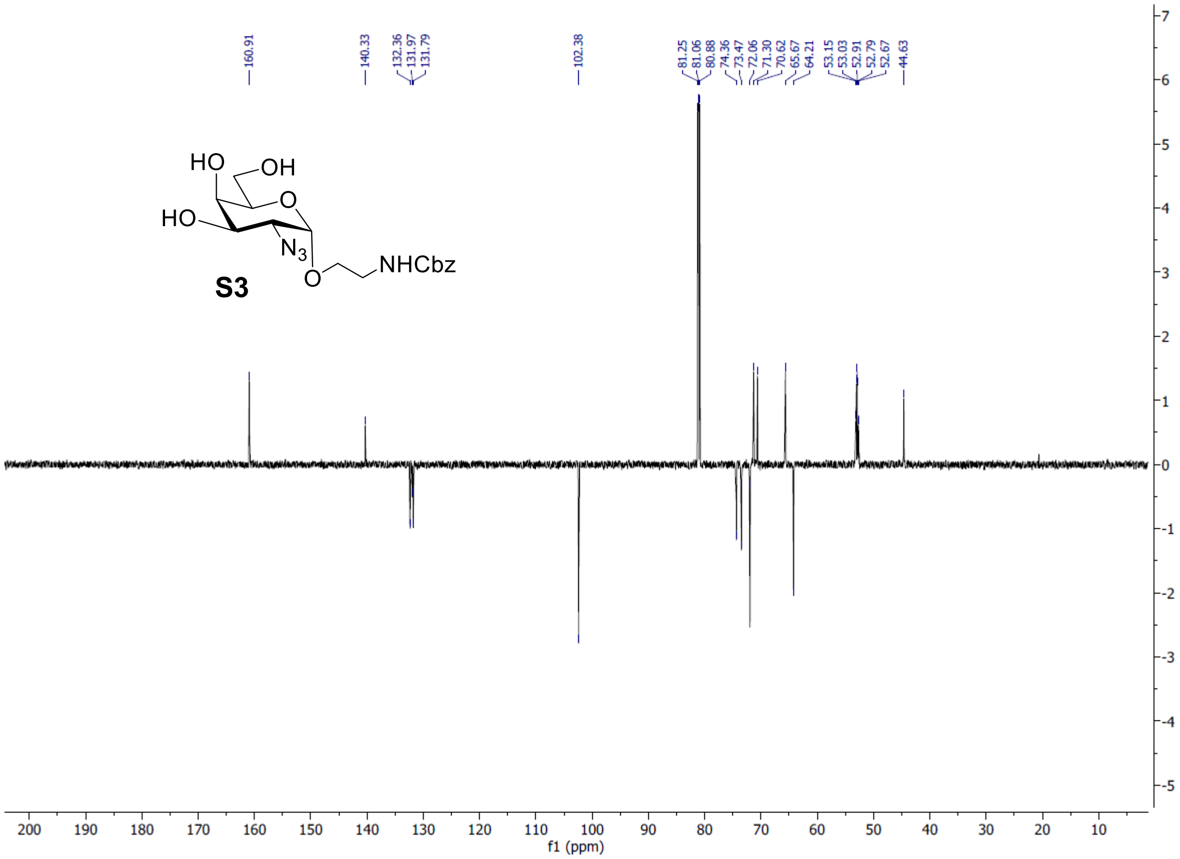

^{13}C NMR spectrum ($CDCl_3$ plus a few drops of CD_3OD, 176 MHz).

Compound **S4**

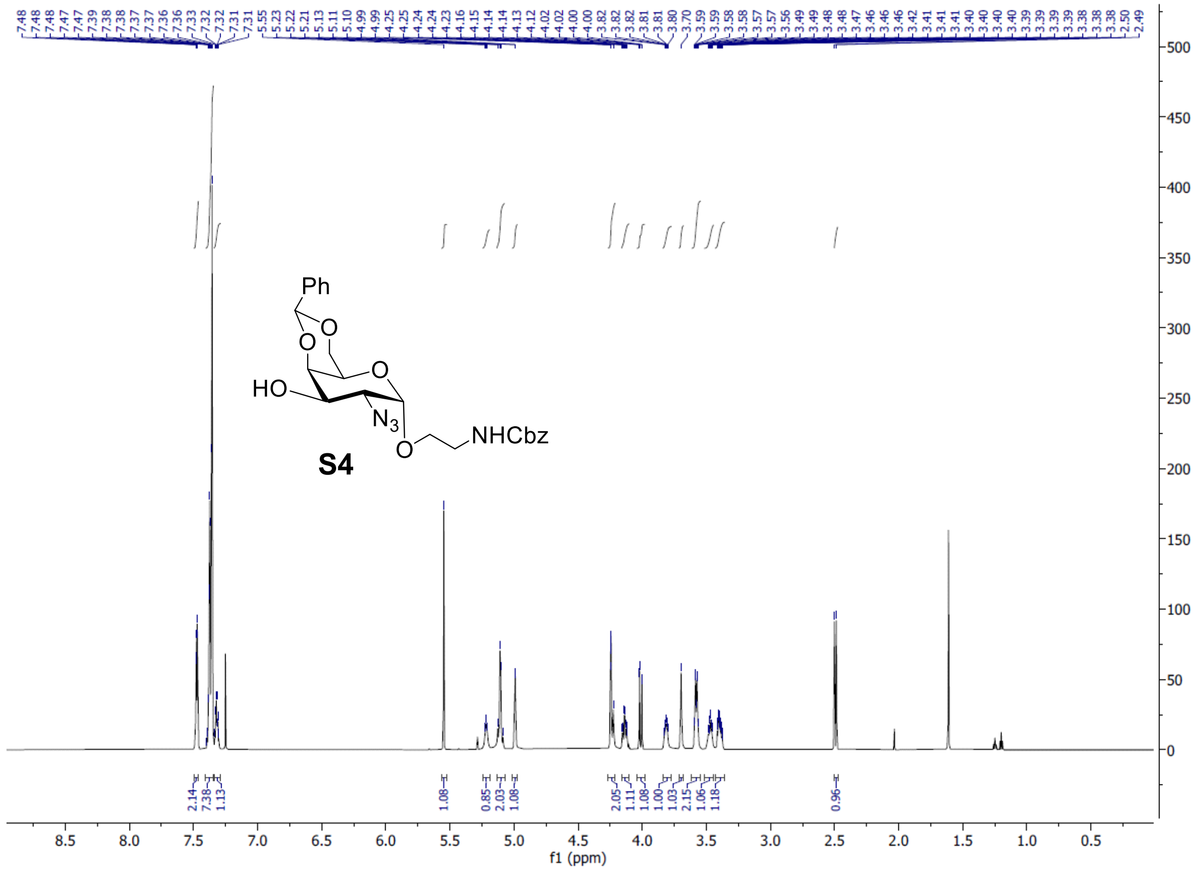

^{1}H NMR spectrum ($CDCl_3$, 700 MHz).

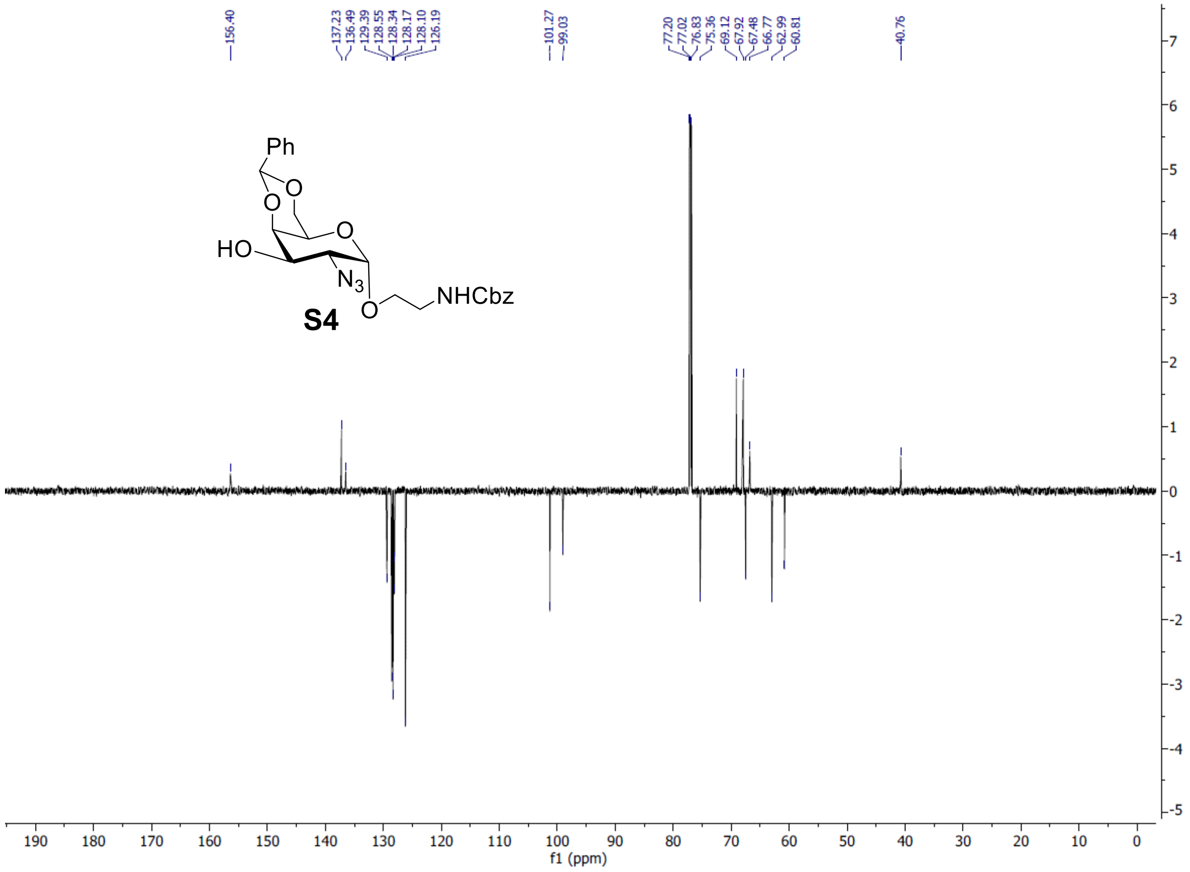

^{13}C NMR spectrum ($CDCl_3$, 176 MHz).

Compound ***S7***

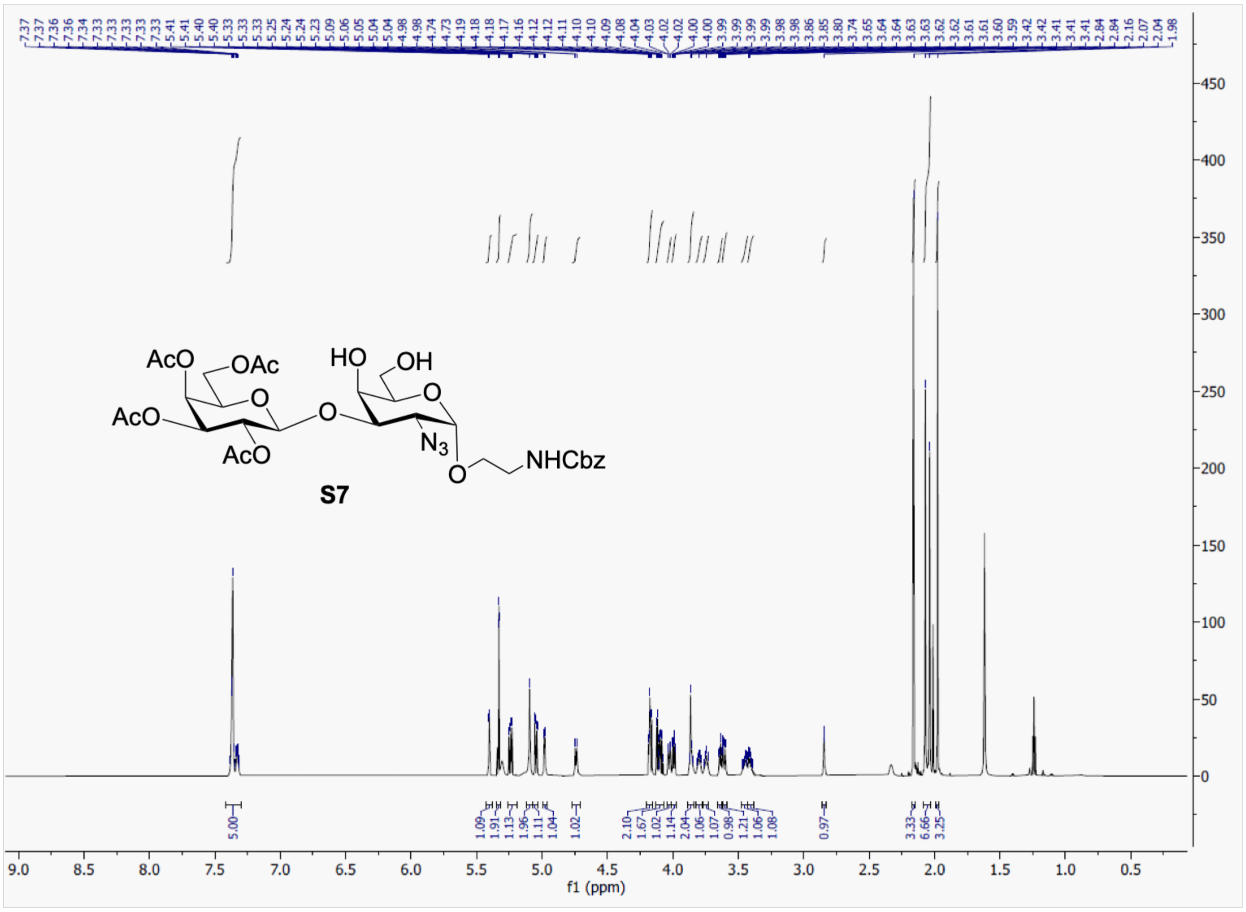

^1H NMR spectrum (CD_2Cl_2, 700 MHz).

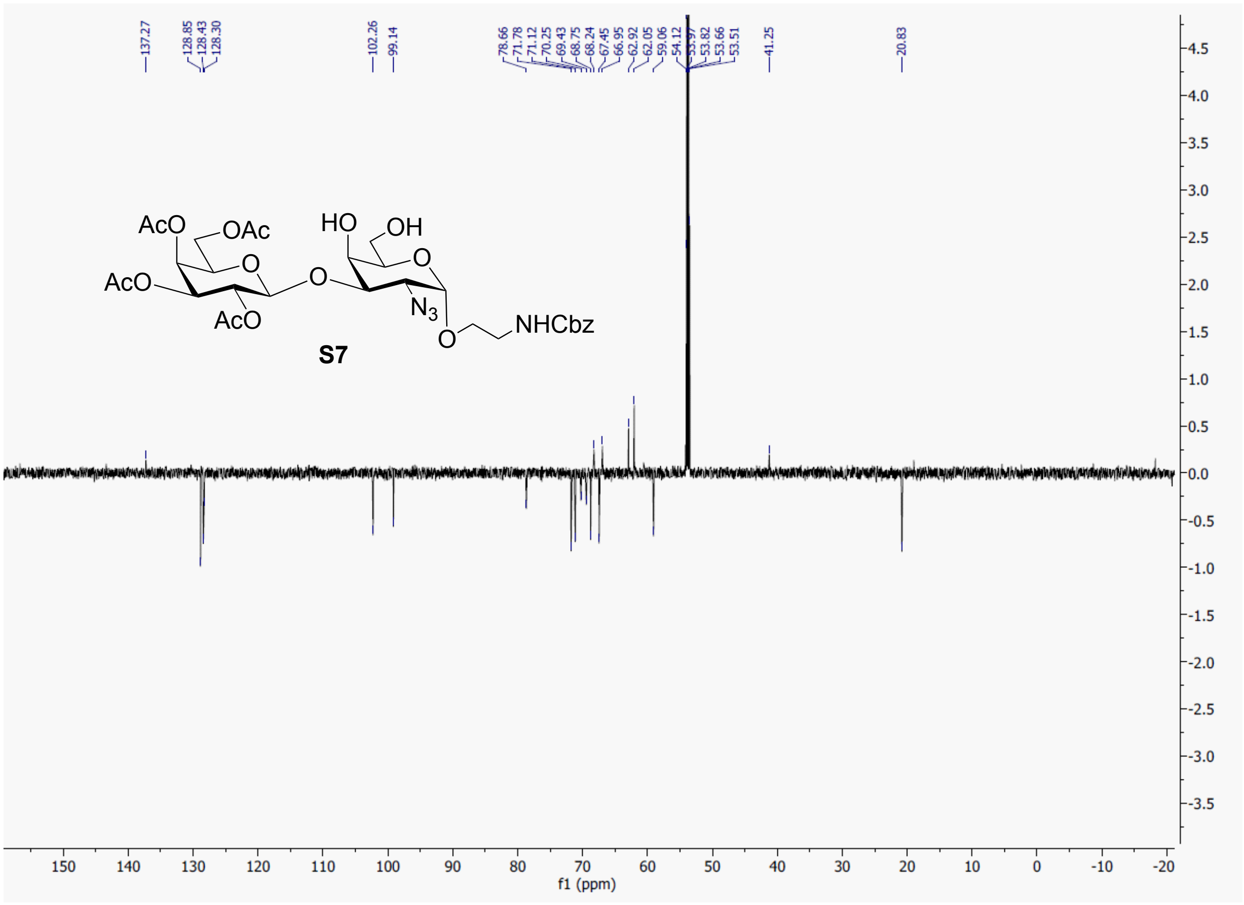

^{13}C NMR spectrum (CD_2Cl_2, 176 MHz).

*Compound **S8***

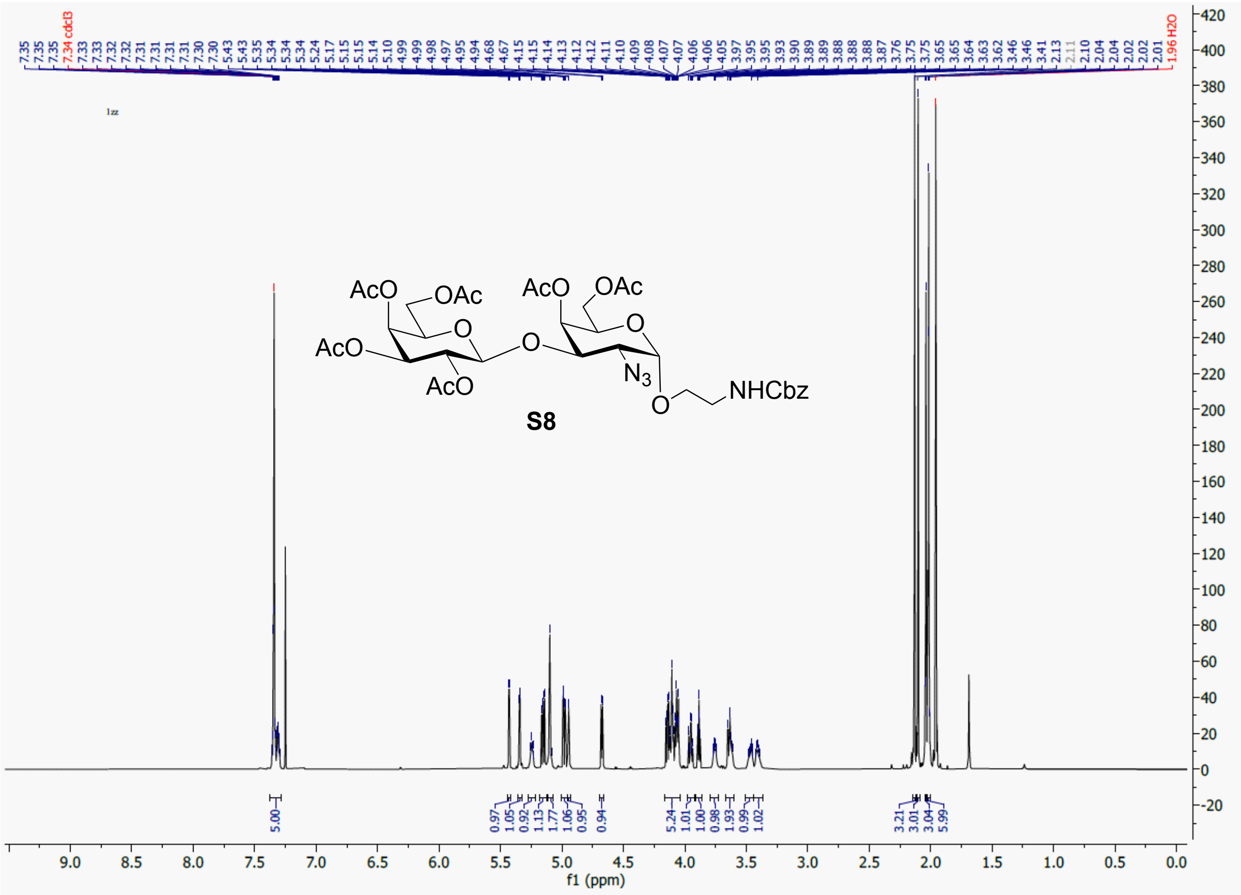

^{1}H NMR spectrum ($CDCl_3$, 700 MHz).

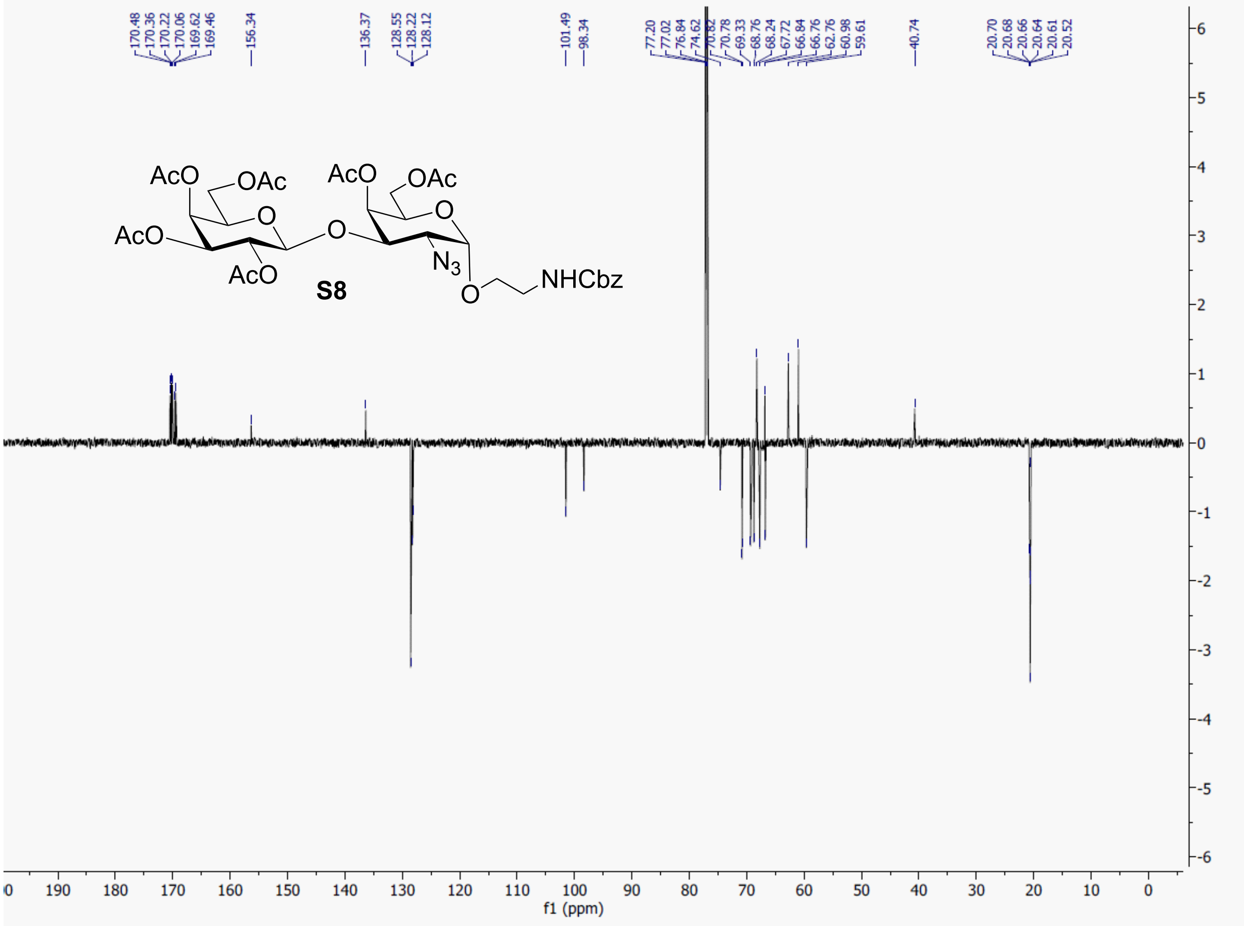

^{13}C NMR spectrum ($CDCl_3$, 176 MHz).

*Compound **S9***

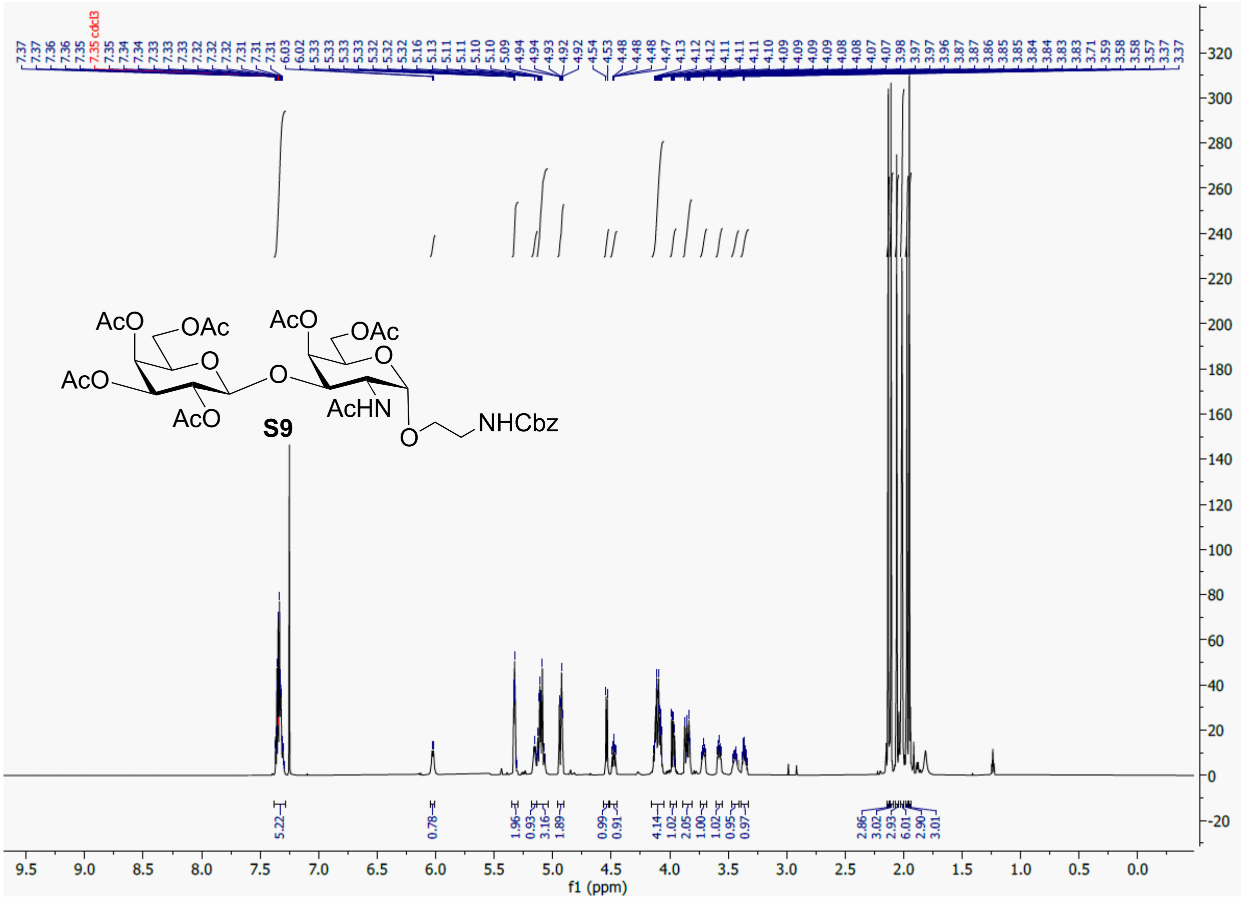

^{1}H NMR spectrum ($CDCl_3$, 700 MHz).

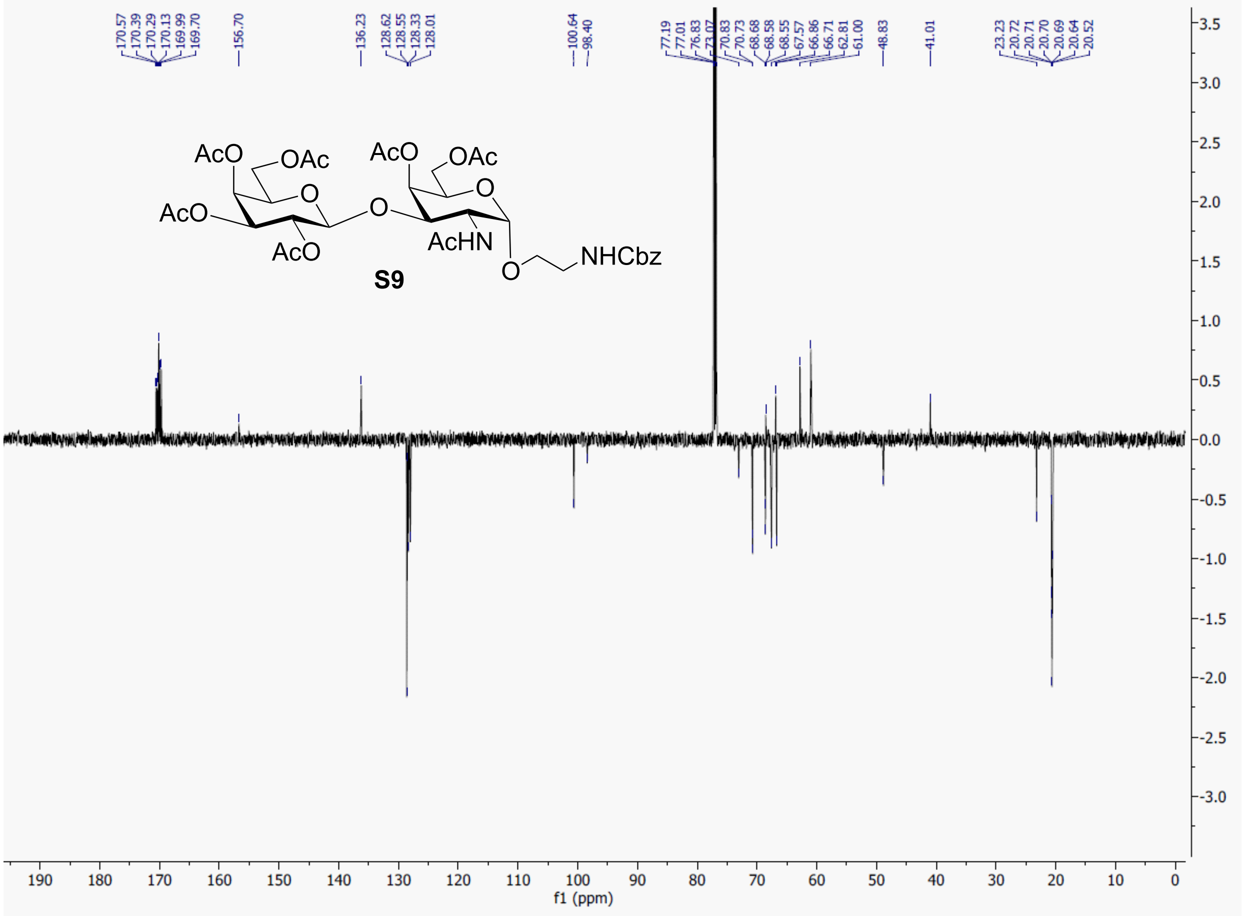

^{13}C NMR spectrum ($CDCl_3$, 176 MHz).

*Compound **12***

^{1}H NMR spectrum (D_2O, 700 MHz).

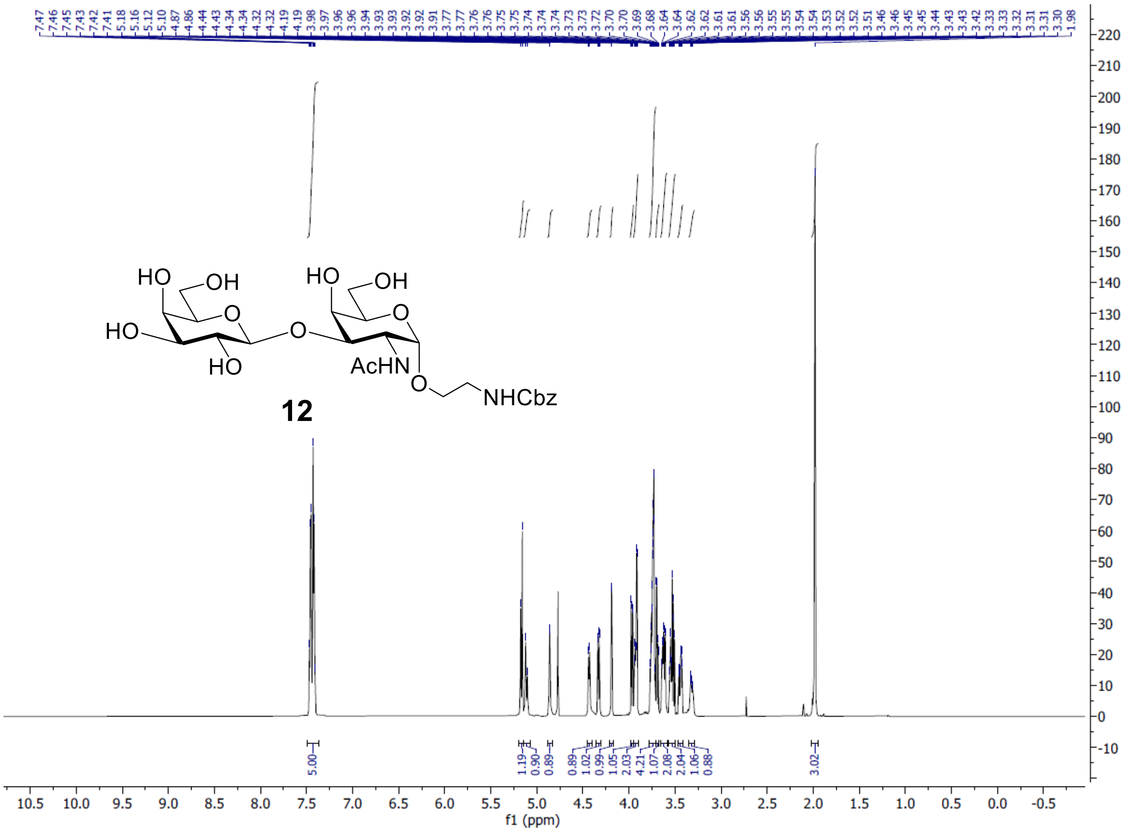

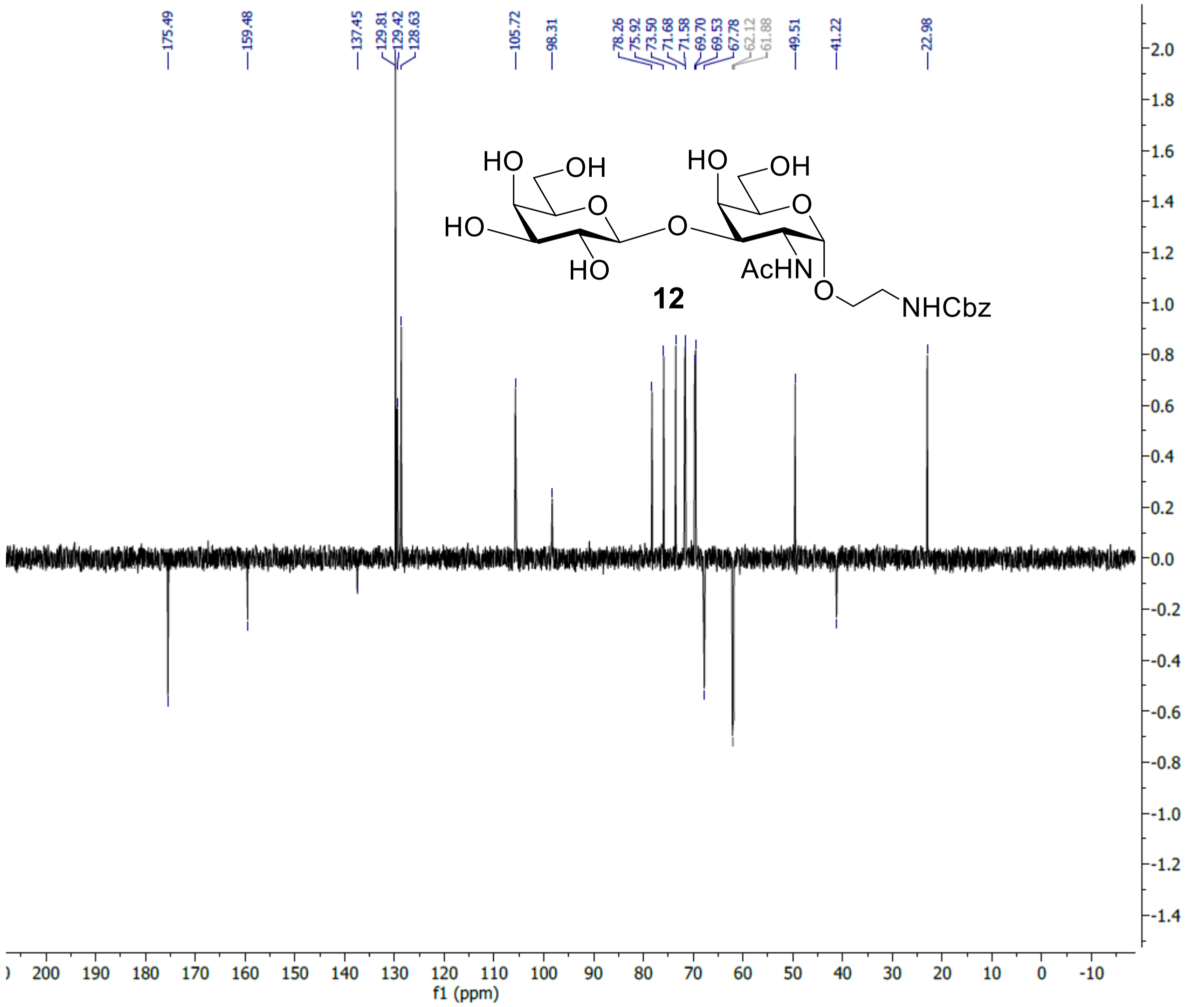

^{13}C NMR spectrum (D_2O, 176 MHz).

*Compound **13***

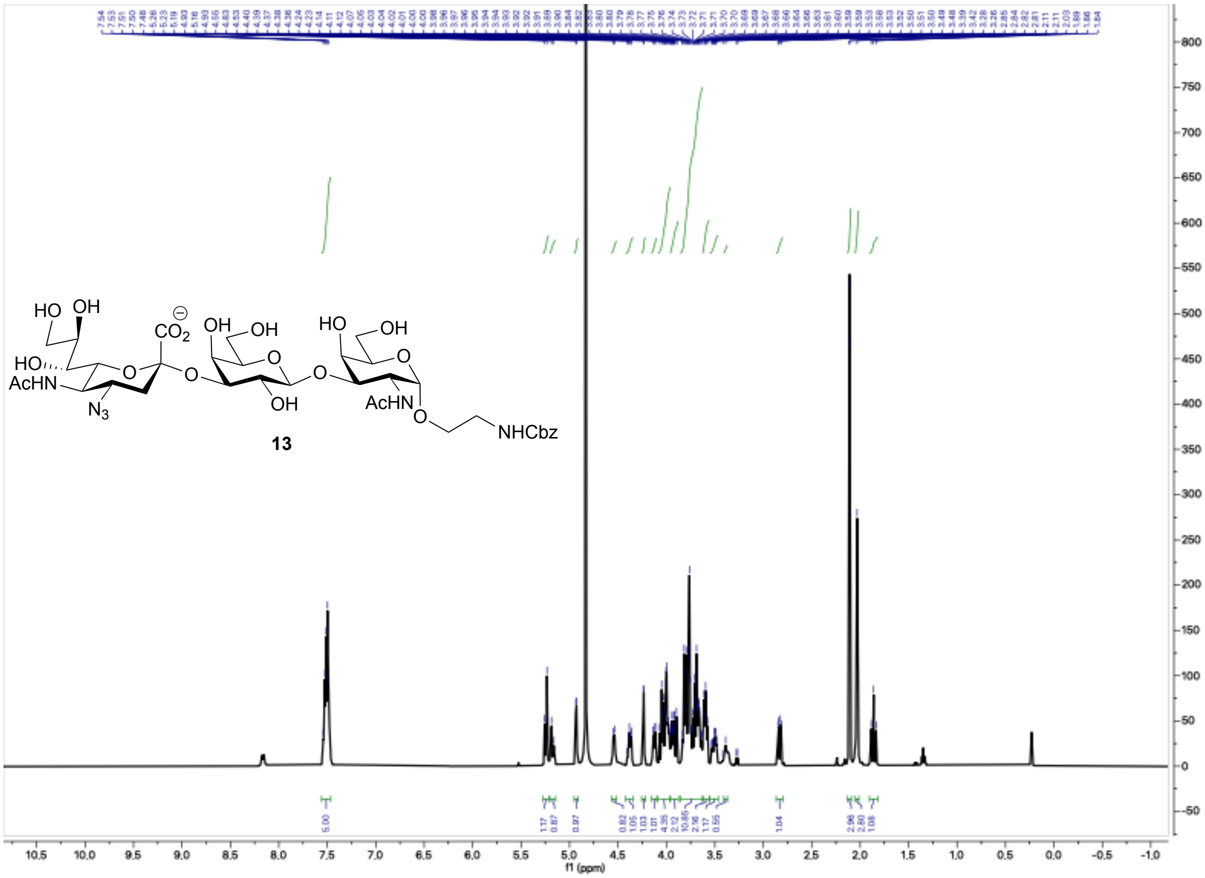

^{1}H NMR spectrum (D_2O, 500 MHz).

408 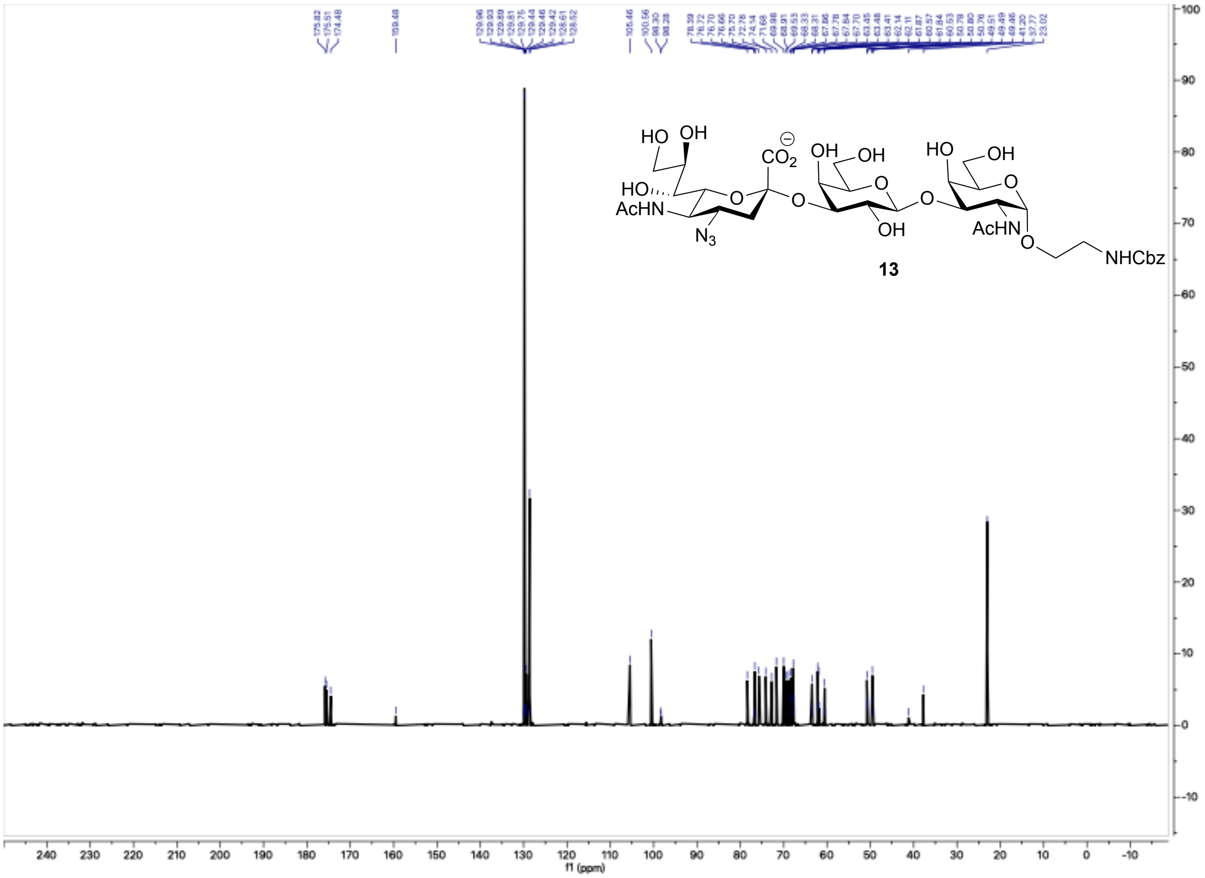

409 ^{13}C NMR spectrum (D_2O, 125 MHz).

*Compound **14***

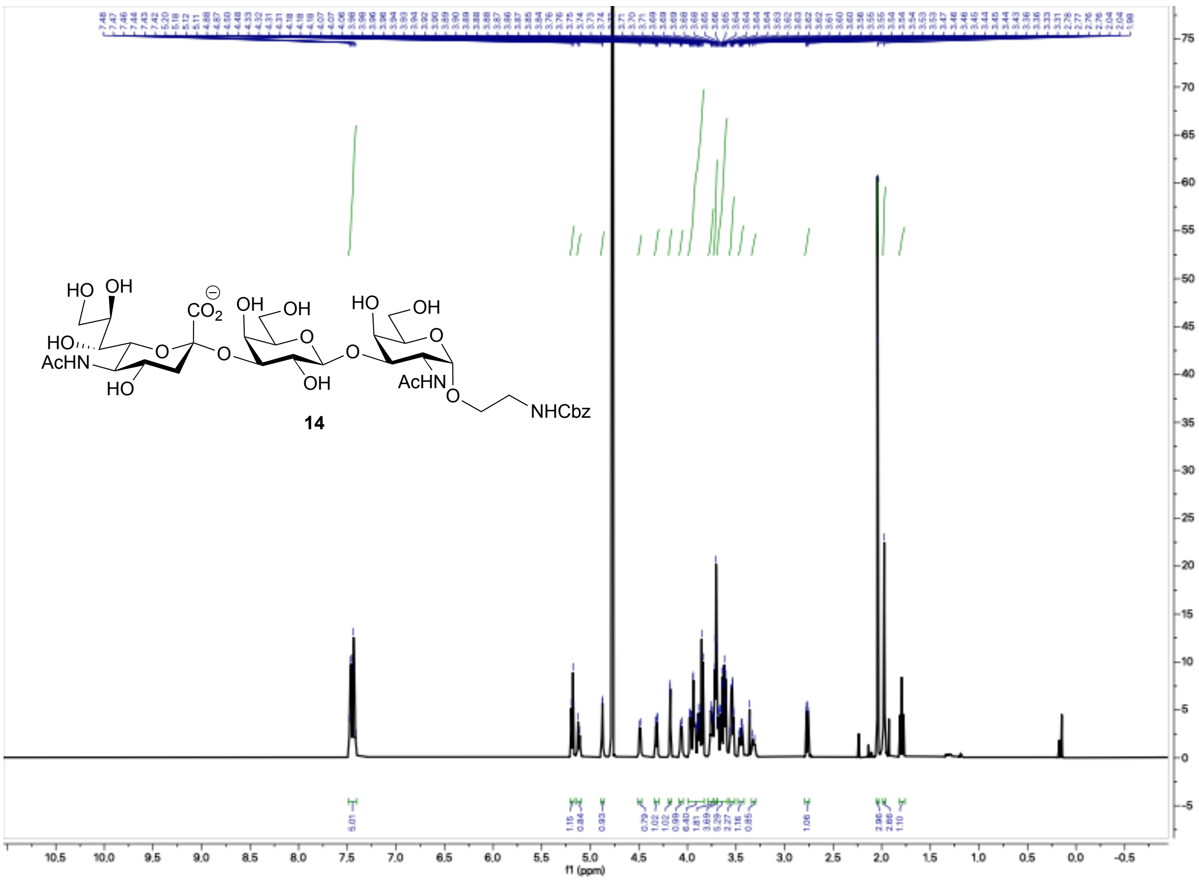

^{1}H NMR spectrum (D_2O, 700 MHz).

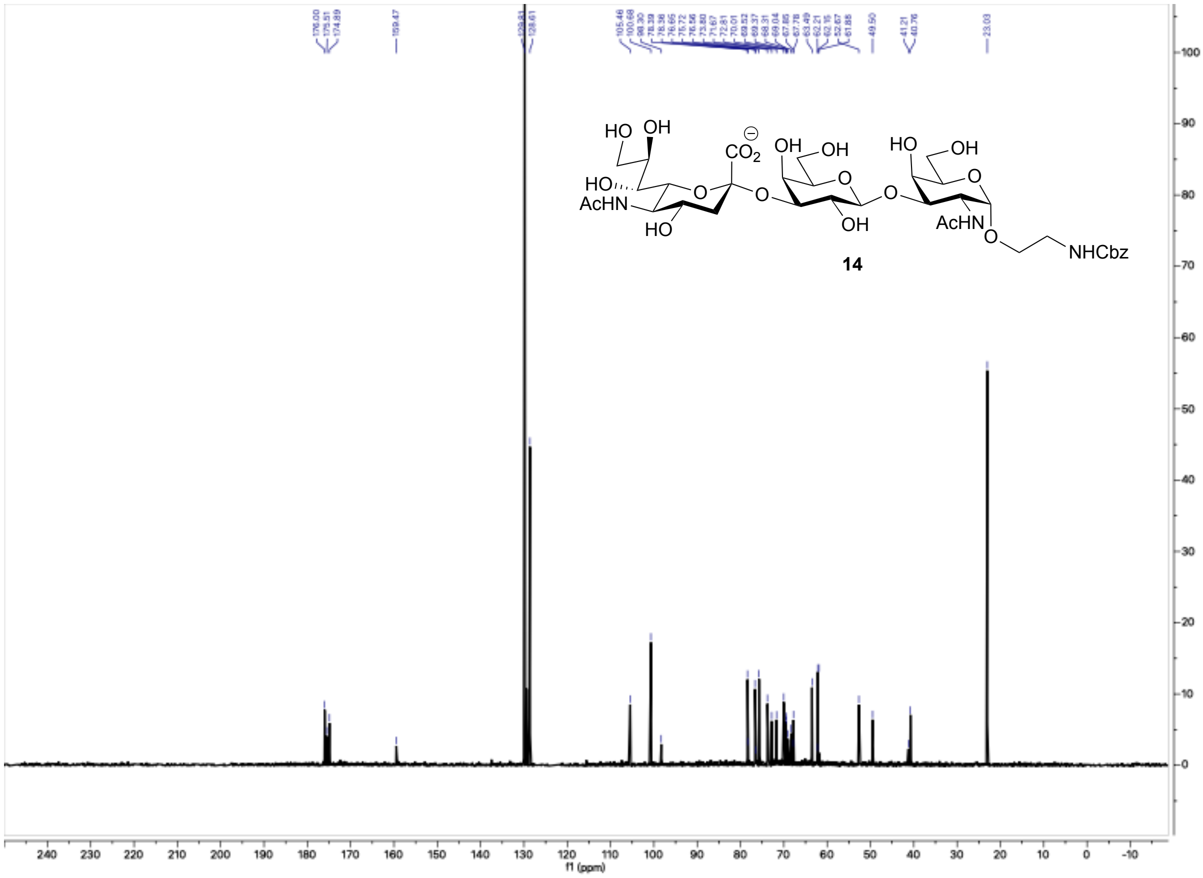

^{13}C NMR spectrum (D_2O, 125 MHz).

References

1. S. Dasgupta, P. I. Kitov, J. M. Sadowska and D. R. Bundle, *Angew Chem Int Ed Engl*, 2014, **53**, 1510-1515.
2. C. Tai, S. Kulkarni and S. Hung, *JOURNAL OF ORGANIC CHEMISTRY*, 2003, **68**, 8719-8722.
3. E. Rodrigues, J. Jung, H. Park, C. Loo, S. Soukhtehzari, E. N. Kitova, F. Mozaneh, G. Daskhan, E. N. Schmidt, V. Aghanya, S. Sarkar, L. Streith, C. D. St Laurent, L. Nguyen, J. P. Julien, L. J. West, K. C. Williams, J. S. Klassen and M. S. Macauley, *Nature Communications*, 2020, **11**, 13.
4. E. N. Schmidt, D. Lamprinaki, K. A. McCord, M. Joe, M. Sojitra, A. Waldow, J. Nguyen, J. Monyror, E. N. Kitova, F. Mozaneh, X. Y. Guo, J. Jung, J. R. Enterina, G. C. Daskhan, L. Han, A. R. Krysler, C. R. Cromwell, B. P. Hubbard, L. J. West, ... and M. S. Macauley, *Nat Commun*, 2023, **14**, 2327.

5. D. Bui, J. Favell, E. Kitova, Z. Li, K. McCord, E. Schmidt, F. Mozaneh, M. Elaish, A. El-Hawiet, Y. St-Pierre, T. Hobman, M. Macauley, L. Mahal, M. Flynn and J. Klassen, *ACS CENTRAL SCIENCE*, 2023, **9**, 1374-1387.
6. P. Kitov, L. Han, E. Kitova and J. Klassen, *JOURNAL OF THE AMERICAN SOCIETY FOR MASS SPECTROMETRY*, 2019, **30**, 1446-1454.
7. D. Kumawat, T. Gray, C. Garnier, D. Bui, Z. Li, Z. Jame-Chenarboo, J. Jerasi, W. Wong, J. Klassen, C. Capicciotti and M. Macauley, *JOURNAL OF THE AMERICAN CHEMICAL SOCIETY*, 2024, **146**, 28630-28634.
